# Systemic Brain Delivery of Oligonucleotide Therapeutics Enhanced by Protein Corona‐Assisted DNA Cubes

**DOI:** 10.1002/smtd.202400902

**Published:** 2024-08-02

**Authors:** Kyoung‐Ran Kim, Ji Hee Kang, Hien Bao Dieu Thai, Ji Hyun Back, Chengde Mao, Ji Eun Lee, Young Tag Ko, Dae‐Ro Ahn

**Affiliations:** ^1^ Chemical and Biological Integrative Research Center Korea Institute of Science and Technology (KIST) Hwarangno 14‐gil 5, Seongbuk‐gu Seoul 02792 Republic of Korea; ^2^ College of Pharmacy Gachon University 191 Hambakmoe‐ro Incheon 21936 Republic of Korea; ^3^ Department of Chemistry Purdue University West Lafayette IN 47907 USA; ^4^ Division of Biomedical Science and Technology KIST School Korea National University of Science and Technology (UST) Hwarangno 14‐gil 5, Seongbuk‐gu Seoul 02792 Republic of Korea

**Keywords:** brain delivery, DNA nanostructures, glioblastoma multiforme, oligonucleotide therapeutics, protein corona

## Abstract

The systemic delivery of oligonucleotide therapeutics to the brain is challenging but highly desirable for the treatment of brain diseases undruggable with traditional small‐molecule drugs. In this study, a set of DNA nanostructures is prepared and screened them to develop a protein corona‐assisted platform for the brain delivery of oligonucleotide therapeutics. The biodistribution analysis of intravenously injected DNA nanostructures reveals that a cube‐shaped DNA nanostructure (D‐Cb) can penetrate the brain‐blood barrier (BBB) and reach the brain tissue. The brain distribution level of D‐Cb is comparable to that of other previous nanoparticles conjugated with brain‐targeting ligands. Proteomic analysis of the protein corona formed on D‐Cb suggests that its brain distribution is driven by endothelial receptor‐targeting ligands in the protein corona, which mediate transcytosis for crossing the BBB. D‐Cb is subsequently used to deliver an antisense oligonucleotide (ASO) to treat glioblastoma multiforme (GBM) in mice. While free ASO is unable to reach the brain, ASO loaded onto D‐Cb is delivered efficiently to the brain tumor region, where it downregulates the target gene and exerts an anti‐tumor effect on GBM. D‐Cb is expected to serve as a viable platform based on protein corona formation for systemic brain delivery of oligonucleotide therapeutics.

## Graphical abstract

In vivo, screening of DNA nanostructures yields a cube‐shaped structure (D‐Cb) as a protein corona‐assisted platform for brain delivery of oligonucleotide therapeutics. D‐Cb effectively delivers polo‐like kinase 1 (PLK‐1) antisense oligonucleotides (ASO) to treat glioblastoma in an orthotopic mouse model, demonstrating its potential as a carrier for brain‐targeted gene therapy.

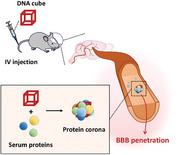

## 1 Introduction

Oligonucleotide therapeutics (OTs) such as small interference RNAs (siRNAs) and antisense oligonucleotides (ASOs) hybridize disease‐related mRNA sequences and downregulate the expression of the downstream protein.^[^ ^1^, ^2^ ^]^ They are a specific and potent therapeutic class to treat diseases undruggable with traditional small molecule drugs. With systemic administration, most of the clinically available OTs are delivered to the liver by lipid nanoparticles (LNPs) or triantennary or *N*‐acetylgalactosamine (GalNac), a hepatocyte‐specific ligand to treat various liver diseases.^[^ ^3^, ^4^ ^]^ However, systemic delivery of OTs into extrahepatic tissues is a challenging task due to the lack of proper delivery methods and highly demanded to expand the therapeutic potential of OTs.^[^ ^5^ ^]^

The blood‐brain barrier (BBB) presents a significant challenge for the delivery of systemically injected therapeutics to the brain. This protective layer of the central nervous system (CNS) blocks the entry of most circulating small molecules and macromolecules into CNS.^[^ ^6^ ^]^ The BBB is composed of brain microvascular endothelial cells (BMEC), a component of the brain capillary and other brain tissue components such as pericytes, smooth muscle cells, and astrocyte projections.^[^ ^7^ ^]^ Only a limited number of hydrophobic substances with low molecular weight (<500 Da) and fewer than 10 hydrogen bonds with solvent water can passively diffuse through the BBB.^[^ ^8^ ^]^ While the highly ionic and hydrophilic small molecules unable to diffuse through the BBB can reach the brain parenchyma via the paracellular pathway, the tight junctions, the gaps between endothelial cells filled with multiprotein junctional complexes maintain the low paracellular permeability of the BBB.^[^ ^9^ ^]^ Nutrients and proteins required for brain function and homeostasis can pass through the BBB by transcytosis mediated by specific transporters and receptors expressed on BMEC, respectively.^[^ ^10^ ^]^ Another transcytosis pathway for BBB penetration is adsorptive‐mediated transcytosis, triggered by non‐specific interactions between positively charged molecules and the negatively charged BMEC membrane.^[^ ^11^ ^]^ Polyanionic macromolecules such as OTs are intrinsically unable to diffuse through the BBB or undergo transcytosis. OTs can be administered into the brain parenchyma via BBB‐bypassing routes such as intraventricular and intrathecal injections.^[^ ^12^ ^]^ However, the invasive nature of these methods may limit their applications. If a systemic route such as intravenous administration through the BBB is available, OTs can be delivered to the brain parenchyma in a relatively less invasive manner.

A prevalent approach to enhancing systemic brain delivery of OTs involves the conjugation of OT‐loaded nanocarriers with ligands that target endocytic receptors in BMEC and promote transcytosis through the BBB.^[^ ^13^ ^]^ Various receptor‐targeting ligands have been conjugated with nanocarriers to improve their distribution within the brain.^[^ ^14^, ^15^, ^16^ ^]^ However, the seemingly straightforward mechanism of this delivery method does not always guarantee an increase in brain distribution when designing a nanocarrier for brain delivery via ligand conjugation. In certain cases, there is no considerable improvement,^[^ ^17^, ^18^ ^]^ or even a decrease in brain delivery compared to unconjugated nanocarriers.^[^ ^19^ ^]^ The formation of a protein corona, a layer of serum proteins adsorbed onto the surface of the nanocarriers, is suspected to hinder the ligand‐based enhancement of brain delivery,^[^ ^19^, ^20^, ^21^ ^]^ while the transcytosis efficiency may also be limited by receptor saturation with high levels of endogenous ligands and/or insufficient stability of the targeting ligands.^[^ ^18^, ^22^ ^]^ Given that the protein corona inevitably forms in situ and unpredictably influences the in vivo biodistribution of nanocarriers, the rational design of nanocarriers for enhanced systemic brain delivery of OTs presents a significant challenge. An alternative strategy to address this issue could involve screening various nanostructures in an in vivo environment to develop a nanocarrier with structural features that enhance brain distribution in a protein‐corona‐assisted manner.

In this context, we have recently demonstrated that the screening of a library consisting of various nucleic acid nanostructures could serve as an effective strategy to develop nanocarriers for the delivery of therapeutics to target tissues.^[^ ^23^, ^24^ ^]^ Additionally, we discovered that their tissue specificity could indeed be affected by the protein corona formed on nanocarriers.^[^ ^23^ ^]^ Moreover, when compared to other nanomaterials, DNA nanostructures serve as highly effective carrier platforms for delivering OTs. These OTs can be loaded with precise control in terms of both ratio and position by incorporating the OT sequences as extensions within the strands forming the structures. As a result, loading occurs seamlessly during the self‐assembly process of the DNA nanostructures, eliminating the need for any additional conjugation step.

**Figure 1:**
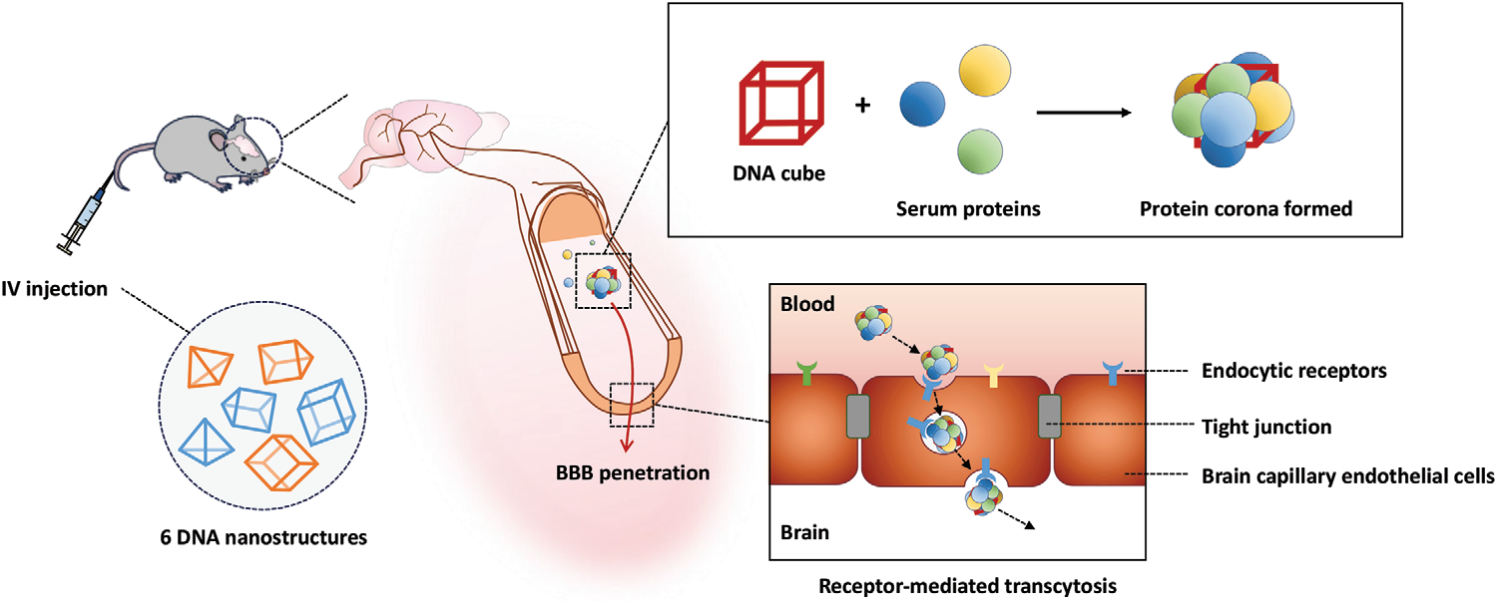
Illustration of the formation of a protein corona on the DNA cube following intravenous (IV) injection, and its contribution to receptor‐mediated transcytosis for BBB penetration.

In this study, we aimed to discover a DNA construct with a protein corona that could enhance brain distribution through in vivo screening of various DNA nanostructures (**Figure** **1**). We prepared six different wireframe DNA nanostructures and examined their biodistribution upon intravenous injection. Our results showed that a cube‐shaped DNA nanostructure had brain distribution properties. We subsequently investigated the role of the protein corona in the brain distribution of this DNA nanostructure. Finally, we employed the DNA nanostructure as a carrier for delivering ASOs to treat glioblastoma multiforme (GBM) in an orthotopic GBM mouse model, thereby evaluating its practical potential as a platform for enhanced systemic brain delivery of OTs.

## 2 Results and Discussion

### 2.1 Preparation and Characterization of Self‐Assembled DNA Nanostructures

**Figure 2:**
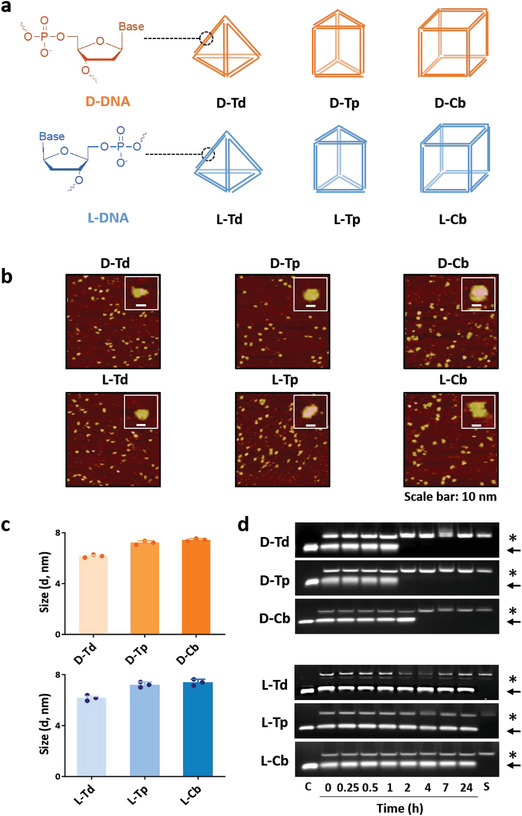
Self‐assembled wireframe DNA nanostructures. a) Schematic structures of 6 DNA nanostructures prepared in this study. b) AFM analysis of DNA nanostructures. Schematic figures of DNA nanostructures shown in the inset images were presented beside AFM images. Scale bar: 10 nm. c) Hydrodynamic sizes of DNA nanostructures estimated on DLS (*n* = 3, mean ± SEM, SEM denotes the standard error of the mean). d) Agarose gel (1%) electrophoresis of DNA nanostructures after incubation in 50% mouse serum. C denotes the control, the structure in absence of serum. S denotes serum only. Asterisks and arrows indicate bands of serum and undamaged structures, respectively.

As smaller nanocarriers have higher possibility of being taken up by cerebral microvessel endothelial cells and crossing BBB than larger ones,^[^ ^24^ ^]^ wireframe DNA nanostructures with a 10 base‐paired duplex per side were designed based on three shapes: tetrahedron (Td), triangular prism (Tp), and cube (Cb), and two types of the deoxyribose backbone: D‐DNA and L‐DNA (**Figure** **2**a). The oligonucleotides for construction of the DNA nanostructures were synthesized by using a DNA synthesizer (Table S1, Supporting Information) and characterized by electrospray ionization mass spectrometry (ESI‐MS) (Table S2, Supporting Information). The DNA nanostructures were prepared via self‐assembly of oligonucleotides through a heating and annealing process, as described in a previous study.^[^ ^25^ ^]^ The self‐assembly of the oligonucleotides was evaluated by polyacrylamide gel electrophoresis (PAGE) which showed bands for the self‐assembled structures with retarded mobility (Figure S1, Supporting Information). DNA nanostructures were imaged by atomic force microscopy (AFM), which roughly revealed the shapes of the DNA nanostructures as designed (Figure 2b). The hydrodynamic sizes of the DNA nanostructures measured by dynamic light scattering were 6.17 ± 0.12 nm for Tds, 7.22 ± 0.15 nm for Tps, and 7.45 ± 0.11 nm for Cbs (Figure 2c). The sizes of DNA nanostructures based on the D‐DNA backbone (D‐Td, D‐Tp, and D‐Cb) were nearly identical to those based on the L‐DNA backbone (6.18 ± 0.20 nm for L‐Td, 7.22 ± 0.20 nm for L‐Tp, and 7.40 ± 0.21 nm for L‐Cb). Serum stability of the DNA nanostructures was also tested. The D‐DNA nanostructures degraded in 50% mouse serum after 2–4 h, while all the L‐DNA nanostructures were highly stable in serum as expected (Figure 2d).

### 2.2 Biodistribution of DNA Nanostructures

**Figure 3:**
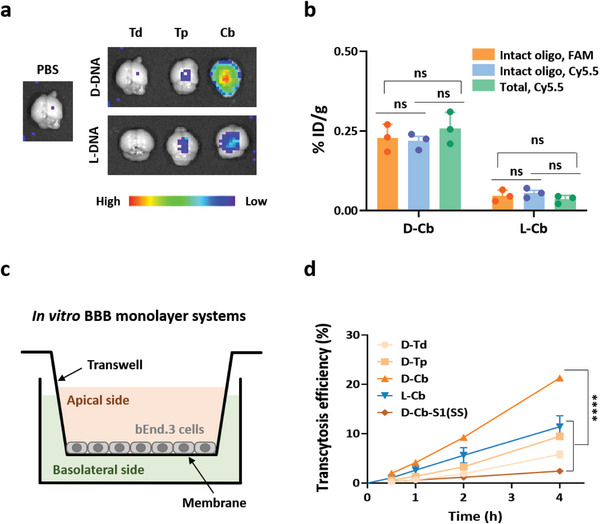
Brain distribution of DNA nanostructures. a) Ex vivo images of mouse brains harvested at 2 h after intravenous injection of DNA nanostructures. Distribution levels of intact D‐Cb and L‐Cb (%ID g^−1^) in the brain estimated by the total Cy5.5 intensity of homogenized brain lysates (total, Cy5.5) and the band intensity of intact oligonucleotides of D‐Cb and L‐Cb labeled with Cy5.5 (intact, Cy5.5) or FAM (intact, FAM) in PAGE of the lysates (*n* = 3, mean ± SEM, SEM denotes the standard error of the mean, ns; no statistically significant). c) Schematic illustration of an in vitro BBB monolayer model. d) Transcytosis efficiency of DNA nanostructures and a single‐stranded (SS) D‐DNA (D‐Cb‐S1) across the BBB model at different time points (*n* = 4, mean ± SEM, ^****^ *p* < 0.0001 vs SS, D‐Tp, D‐Td, and L‐Cb).

After preparation and characterization of the DNA nanostructures, we examined their in vivo biodistribution in mice. Mice were intravenously injected with the Cy5.5‐labeled DNA nanostructures and monitored for 24 h (Figure S2, Supporting Information). In vivo imaging showed that all the DNA nanostructures except D‐Tp were distributed throughout the body within 1 h after injection. At 2 h post‐injection, major organs were harvested from the mice and imaged to estimate the distribution level of the nanostructures in each organ using Cy5.5 intensity (Figure S3, Supporting Information). Most of the DNA nanostructures were distributed to the liver and kidneys. Focusing on brain distribution, D‐Cb showed the highest distribution level, followed by L‐Cb and L‐Tp (**Figure** **3**a). Although the three nanostructures (Td, Tp, and Cb) are similar in size, their shapes and sequence compositions differ significantly. For instance, Cb has 12 duplex sides, whereas Td and Tp have 6 and 9, respectively. The geometrical features of Cb create a spatial interface distinct from that of Td or Tp, potentially leading to different interactions with biological factors such as serum proteins responsible for brain distribution. Compared to the structures of Td and Tp, the Cb structure appears to facilitate brain delivery within the same sugar backbone type (Figure 3a).

The brain distribution level of D‐Cb was ≈0.25%ID/g as quantified by measuring the Cy5.5 intensity of brain lysate (Figure 3b). Since Cy5.5 intensity reports the sum of intact and fragmented structures, we also attempted to estimate only the amount of intact D‐Cb distributed in the brain by quantifying the band of the intact strand (Cy5.5‐S1) in D‐Cb on denaturing PAGE of the homogenized brain tissue lysates (Figure S4, Supporting Information). The brain distribution level of intact D‐Cb was 0.22%ID/g at 2 h post injection, which was approximately fourfold higher than that of intact L‐Cb although D‐Cb is less stable in serum than L‐Cb (Figure 3b and Figure 2d). Even when the DNA nanostructures were labeled with a negatively charged dye (fluorescein, FAM) instead of a positively charged dye (Cy5.5), their brain distribution levels did not change significantly (Figure 3b). This indicates that the brain distribution property of D‐Cb was not driven by the fluorescence label. When biotinylated D‐Cb was injected, the brain distribution of D‐Cb could also be detected by mass analysis of the biotinylated strand (S5) in D‐Cb, which was pulled down with streptavidin‐coated magnetic beads from brain lysate (Figure S5, Supporting Information). Interestingly, the brain distribution level of D‐Cb, even without brain‐targeting ligands, was comparable to that of some of the other nanoparticles conjugated with brain‐targeting ligands such as ANG, bovine serum albumin (BSA), and transferrin.^[^ ^26^, ^27^, ^28^, ^29^ ^]^ This demonstrates that the protein corona‐assisted brain delivery can similarly be efficient as the targeted delivery based on ligand conjugation.

To investigate whether the brain‐reaching property of D‐Cb is based on BBB penetration, we estimated the BBB‐penetration potential of D‐Cb using an in vitro BBB monolayer model (Figure 3c). FAM‐labeled D‐Cb was added to the apical side of the in vitro BBB model. The amount of the transcytosed DNA nanostructures was estimated by measuring FAM intensity in the basolateral side (Figure 3c; Figure S6a, Supporting Information). The transcytosis efficiency of D‐Cb increased with the incubation time (Figure 3d). The same cube structure with the mirrored sugar backbone (L‐Cb) or different shapes with the same D‐backbone (D‐Td and D‐Tp) showed lower transcytosis efficiency than D‐Cb (Figure 3d), indicating that the proper combination of the backbone and the shape is critical for crossing BBB. Transepithelial/transendothelial electrical resistance (TEER) did not vary after treatment with the DNA nanostructures, indicating that the integrity of the tight junction was maintained during the transcytosis and not disrupted by treatment (Figure S6b, Supporting Information). The apparent permeability coefficient (*P_app_*) of D‐Cb was also higher than that of any structures sharing either the backbone or the shape with D‐Cb, consistently evidencing the potential of the D‐Cb structure for BBB penetration for brain delivery (Figure S6c, Supporting Information). Unassembled single‐stranded DNA strands were hardly able to penetrate the monolayer model, suggesting that the 3D D‐Cb structure is critical for transcytosis (Figure 3d; Figure S6a, Supporting Information).

### 2.3 Proteomic Analysis of the Protein Corona on D‐Cb and L‐Cb

**Figure 4:**
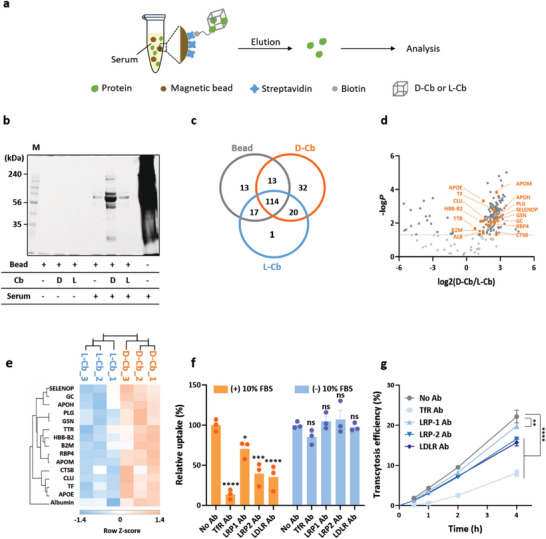
The effect of protein corona on D‐Cb for brain delivery. a) Schematic presentation on the preparation of the protein corona by incubating biotinylated D‐Cb and L‐Cb immobilized on streptavidin coated‐magnetic beads in mouse serum. b) SDS‐PAGE (12%) analysis of proteins pulled down with D‐Cb and L‐Cb from mouse serum. The protein bands were stained by Coomassie Blue. M denotes the size marker. c) Venn diagrams showing the number of identified proteins from three replicates of LC‐MS/MS runs of each sample group. d) The volcano plot of 210 proteins identified in the pull‐down proteins with D‐Cb and L‐Cb. Those above a gray horizontal line are 165 statistically significant proteins (*p*‐value < 0.05). Orange spots indicate 15 protein ligands of TfR, LRP1, and LRP2: selenoprotein P (SELENOP), vitamin D‐binding protein (GC), β−2‐glycoprotein 1 (APOH), plasminogen (PLG), gelsolin (GSN), transthyretin (TTR), hemoglobin subunit β−2 (HBB‐B2), β−2‐microglobulin (B2M), retinol‐binding protein 4 (RBP4), apolipoprotein M (APOM), cathepsin B (CTSB), clusterin (CLU), serotransferrin (TF), and apolipoprotein E (APOE). e) The heat map displaying hierarchical clustering of the 15 proteins with statistically significant changes (*p*‐value < 0.05) between two types of samples pulled down with D‐Cb and L‐Cb. The rows represent each protein and the columns show three LC‐MS/MS runs of samples pulled down with bead, L‐Cb, and D‐Cb. Hierarchical clustering of the 15 proteins was performed in Perseus software (1.6.14.0) on log‐transformed normalized abundance values after z‐score normalization of the data. f) Flow cytometric analysis of bEnd.3 cells after treatment with D‐Cb in 10% FBS in the presence or absence of anti‐TfR, ‐LRP1, ‐LRP2, or ‐LDLR antibodies (*n* = 3, mean ± SEM, ns; no statistically significant, ^*^ *p* < 0.05. ^***^ *p* < 0.001, ^****^ *p* < 0.0001 vs No Ab groups. Ab denotes antibody. g) Transcytosis efficiency of D‐Cb in 10% FBS‐containing medium in the presence or absence of antibodies (*n* = 4, mean ± SEM) ^**^ *p* < 0.01 vs LRP1 Ab, ^****^ *p* < 0.0001 vs TfR Ab, LRP2 Ab, and LDLR Ab.

After observing significant levels of D‐Cb distribution within the brain, we sought to identify the factors responsible for directing D‐Cb to this organ. The serum half‐life of D‐Cb, exceeding 2 h, might allow a significant quantity of intact D‐Cb in the circulation to be distributed to the brain at 2 h post injection. However, given that L‐Cb, which is much more stable than D‐Cb in serum, showed a lower level of brain distribution, there could be other factors responsible for the brain distribution of D‐Cb. Recent studies have demonstrated the critical role of the protein corona in the biodistribution of DNA nanostructures.^[^ ^23^, ^30^ ^]^ Based on these findings, we hypothesized that the in situ formation of a protein corona on D‐Cb following intravenous injection may contribute to its distribution within the brain. To investigate the formation of a protein corona by serum proteins, we used mass spectrometry (MS)‐based proteomic analysis to identify the proteins in mouse serum that could form a corona on D‐Cb (**Figure** **4**a). Sodium dodecyl sulfate (SDS)‐PAGE analysis revealed significantly higher protein adsorption on D‐Cb compared to L‐Cb (Figure 4b). The amount of proteins pulled down with L‐Cb was similar to the background level observed with naked magnetic beads. Mass spectral analysis identified 210 proteins bound to the three types of samples, with two or more unique peptides assigned (Table S3, Supporting Information). Of these, 157, 179, and 152 proteins were identified in samples pulled down with beads, D‐Cb, and L‐Cb, respectively, with 114 shared proteins found among the three types of samples (Figure 4c). Label‐free quantitative analysis based on peak areas among the three sample groups revealed 165 statistically significant proteins (*p*‐value < 0.05) using one‐way analysis of variance (ANOVA) from comparison of the log_2_(normalized abundance) values (Figure 4d; Table S4, Supporting Information).

Hierarchical clustering analysis of the 165 proteins exhibiting statistically significant changes among the three types of samples pulled down with beads, D‐Cb, and L‐Cb revealed that ≈80% of the proteins pulled down with D‐Cb showed increased abundance levels (>1.50‐fold increases) compared to those pulled down with beads and L‐Cb (Figure S7, Supporting Information). These 165 proteins, with a *p*‐value < 0.05 from ANOVA analysis, were further subjected to Student's *t*‐test analysis to identify proteins exhibiting statistically significant changes between those pulled down with D‐Cb and L‐Cb. Of the 165 proteins, 133 showed statistically significant changes (*p*‐value < 0.05) between those pulled down with D‐Cb and L‐Cb. All 133 proteins exhibited increased protein abundance when pulled down with D‐Cb compared with L‐Cb (Table S4; Figure S8, Supporting Information for hierarchical clustering analysis of the 133 proteins). Among these 133 proteins, 15 were ligands of cell surface endocytic receptors known to mediate endothelial transcytosis for BBB penetration, such as transferrin receptor 1 (TfR1), lipoprotein receptor‐related protein 1 (LRP1), and LRP2 (Table S5, Supporting Information; Figure 4d).^[^ ^31^ ^]^ ApoE, the previously known ligand for brain delivery via low‐density lipoprotein receptor (LDLR)‐mediated transcytosis, was also more abundantly found in the protein corona of D‐Cb.^[^ ^32^ ^]^ A heat map showing the hierarchical clustering of these 15 proteins is presented in Figure 4e.

To determine whether brain distribution of D‐Cb was based on TfR1, LRP1, LRP2, and LDLR‐mediated endothelial transcytosis, we examined the uptake of D‐Cb with a protein corona into mouse endothelial cells (bEnd.3) following inhibition of the major receptors involved in the transport of various ligands across BBB (Figure 4f). In the presence of serum, the cellular uptake level of D‐Cb was significantly inhibited by antibodies against these receptors. However, in the absence of serum, the cellular uptake efficiency of D‐Cb was not significantly affected by antibodies against TfR1, LRP1, LRP2, and LDLR. The uptake level of D‐Cb was reduced by 80% with TfR1 Ab, while inhibition of the uptake by LRP1 Ab was less than 30%. The uptake level was moderately decreased by both LRP2 Ab and LDLR Ab. These results suggest that the BBB penetration of D‐Cb is mainly mediated by TfR1 via interaction with transferrin in the protein corona, although all the receptors examined play a part in transcytosis. Similarly, transcytosis of D‐Cb through an in vitro BBB monolayer was also significantly reduced by the antibodies in the presence of serum (Figure 4g; Figure S9a, Supporting Information), but the antibody effect on transcytosis of D‐Cb in the monolayer model was negligible without serum. These results indicate that endothelial transcytosis of D‐Cb for BBB penetration is dependent on the protein corona‐containing ligands for endothelial receptors. In contrast, the cellular uptake efficiency of L‐Cb in the endothelial cells was not affected by the presence of antibodies regardless of the serum (Figure S9b, Supporting Information). This suggests that the protein corona‐assisted transcytosis of L‐Cb is unlikely, possibly due to its low protein adsorption property (Figure 4b). The tight junction was not disrupted by the treatment of antibodies and Cbs, as evaluated by TEER levels (Figure S9c, Supporting Information).

### 2.4 D‐Cb as a Platform for Targeting GBM

**Figure 5:**
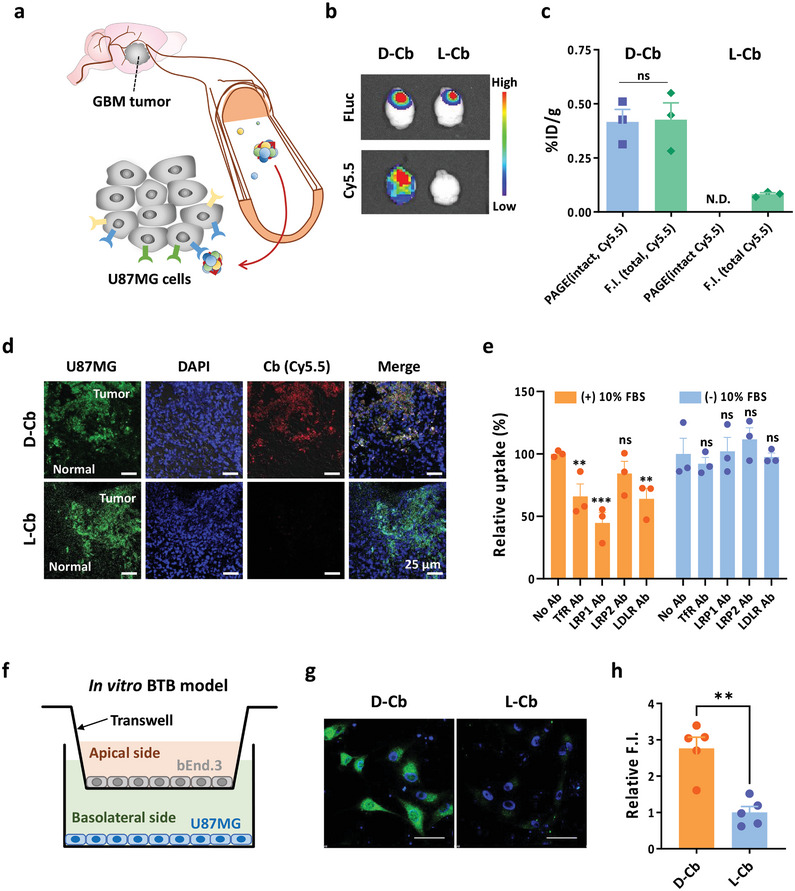
Protein corona‐assisted distribution of D‐Cb to GBM tumor. a) Schematic figure showing protein corona‐assisted distribution of D‐Cb to GBM tumor. b) Ex vivo images of brains harvested from GBM mice at 2 h after intravenous injection of Cy5.5‐labeled DNA nanostructures. The tumor region was imaged with luminescence (FLuc). c) Distribution levels of D‐Cb and L‐Cb (%ID g^−1^) in the brain estimated by the total Cy5.5 intensity (F.I.) of homogenized brain lysates (total, Cy5.5) and the band intensity of intact oligonucleotides of D‐Cb and L‐Cb labeled with Cy5.5 (intact, Cy5.5) in PAGE of the lysates (*n* = 3, mean ± SEM, SEM denotes the standard error of the mean, ns; no statistically significant). d) Representative fluorescence images of brain tissue sections. Green: tumor (U87MG‐FLuc‐GFP), blue: nuclei, red: D‐Cb or L‐Cb, scale bar: 25 µm. e) Flow cytometric analysis of bEnd.3 cells after treatment with D‐Cb in 10% FBS in the presence or absence of anti‐TfR, ‐LRP1, ‐LRP2, or ‐LDLR antibodies (*n* = 3, mean ± SEM, ns; no statistically significant, ^*^ *p* < 0.05. ^***^ *p* < 0.001, ^****^ *p* < 0.0001 versus No Ab groups. Ab denotes antibody. f) Schematic illustration of an in vitro BTB monolayer model. g) Fluorescence microscopic images of U87MG cell following transcytosis of FAM‐labeled D‐Cb or L‐Cb (green) through endothelial cells in the in vitro BTB model. Nuclei were stained with DAPI (blue). Scale bar: 50 µm. h) Relative FAM fluorescence intensity (F.I.) of U87MG cells in (g) (*n* = 5, mean ± SEM, ^**^ *p* < 0.01).

After observing the potential of protein corona on D‐Cb for penetrating BBB, we hypothesized that D‐Cb, when coated with a protein corona containing ligands of endocytic receptors, could serve as a platform for targeting GBM in the brain, as these receptors are also abundantly expressed in GBM cells such as U87MG^[^ ^33^, ^34^, ^35^ ^]^ (**Figure** **5**a). To examine the GBM‐targeting potential in vivo, we injected D‐Cb intravenously into mice bearing orthotopic GBM, generated by intracranial injection of firefly luciferase (FLuc)‐ and green fluorescence protein (GFP)‐expressing U87MG cells (U87MG‐FLuc‐GFP), and analyzed its distribution in the brain tissue. Representative ex vivo imaging of the brain at 2 h post injection revealed a clear brain distribution of D‐Cb in the brain of GBM‐bearing mice (Figure 5b). The brain distribution level of D‐Cb in GBM‐bearing mice was ≈0.42%ID g^−1^, as quantified by measuring the intensity of Cy5.5 in brain lysate, and 0.43%ID g^−1^, as quantified by PAGE analysis to estimate the amount of intact form of the structure. This was higher than the brain distribution level of D‐Cb in healthy mice (Figure 5c; Figure S10, Supporting Information). Confocal fluorescence microscopic images of brain sections showed D‐Cb accumulated in the tumor region (Figure 5d). The enhanced brain distribution and tumor accumulation of D‐Cb could be due to the endocytic receptors expressed in U87MG cells as well as in the endothelial cells, which interact with the ligands in the protein corona on D‐Cb. Indeed, cellular uptake of D‐Cb into U87MG cells was significantly inhibited by antibodies against these receptors in the presence of serum (Figure 5e), which can promote the formation of a protein corona on D‐Cb. However, the uptake level was not significantly affected by the antibodies in the absence of serum. Internalization of D‐Cb into U87MG cells following transcytosis through endothelial cells was examined using an in vitro blood‐tumor barrier (BTB) model (Figure 5f). D‐Cb added in the apical side were transcytosed through endothelial cells (bEnd.3) to reach the basolateral side and subsequently internalized into U87MG cells (Figure 5g). These results support the in vivo tumor distribution of D‐Cb in the brain.

For comparison, we intravenously injected L‐Cb into GBM‐bearing mice. Neither significant brain distribution nor accumulation of L‐Cb in the tumor was observed (Figure 5b–d). In addition, the cellular uptake efficiency into U87MG cells was unaffected by the presence of the antibodies irrespective of the serum (Figure S11, Supporting Information). This is in line with the cellular uptake pattern observed in bEnd.3 cells (Figure 4f). In the in vitro BTB model, the internalization of L‐Cb into U87MG cells was found to be approximately threefold lower than that of D‐Cb (Figure 5g,h). This could be attributed to the lack of the protein corona on L‐Cb required for interaction with the endocytic receptors, which may restrict its penetration through the BBB and BTB prior to reaching U87MG cells and its uptake into the glioblastoma cells even after penetration. Overall, these results suggest that the ligands of the endocytic receptors in protein corona are responsible for the accumulation of D‐Cb in U87MG tumors in the brain.

### 2.5 Systemic Brain Delivery of ASO Using D‐Cb for Treatment of GBM

**Figure 6:**
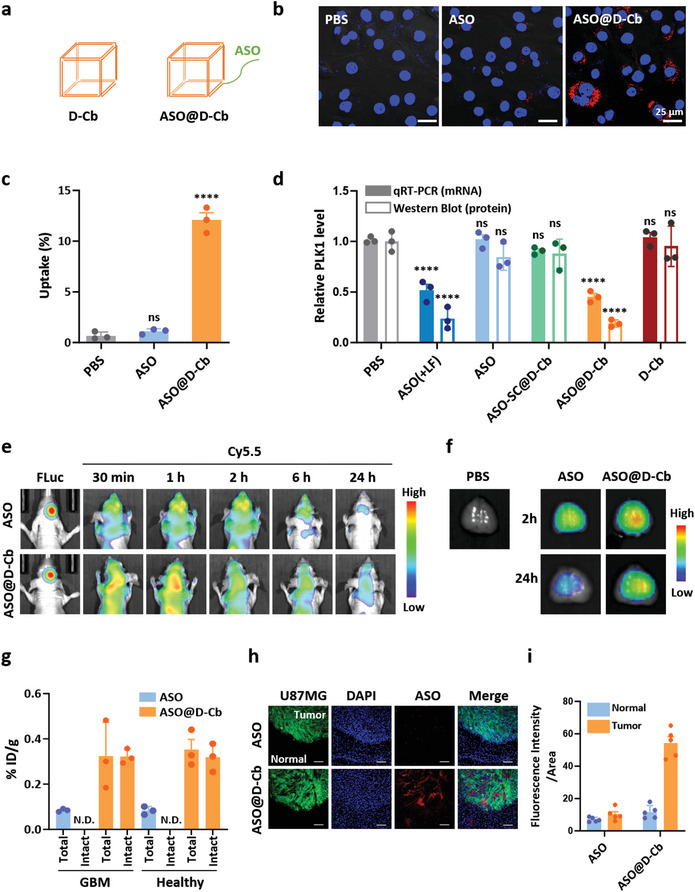
Brain delivery of PLK1 ASO using D‐Cb. a) Schematic structures of PLK1 ASO‐loaded D‐Cb (ASO@D‐Cb). b) Confocal microscopic images of U87MG cells after treatment of PBS, ASO, and ASO@‐Cb. Magnification 400X, scale bar: 25 µm, blue: nuclei, red: ASO. c) Cellular uptake levels of ASO and ASO@D‐Cb in U87MG cells (ns; no statistically significant, ^****^ *p* < 0.0001 vs PBS group). d) Relative PLK1 mRNA and protein levels in U87MG cells after treatment with ASO@D‐Cb were analyzed by qRT‐PCR and western blotting, respectively. LF denotes lipofectamine RNAiMax (*n* = 3, mean ± SEM, ns; no statistically significant, ^****^ *p* < 0.0001 vs PBS‐treated group). e) Time‐dependent brain distribution of ASO or ASO@D‐Cb in GBM mice (U87MG‐FLuc‐GFP orthotopic mouse model). f) Ex vivo images of brains showing the distribution of ASO and ASO@D‐Cb at 2 or 24 h post injection (2 µM, 200 µL). g) Brain distribution levels (%ID/g) of ASO or ASO@D‐Cb estimated by the total Cy5.5 intensity of homogenized brain lysates from healthy or GBM mice (total) and the band intensity of Cy5.5‐labeled intact oligonucleotides in PAGE of the lysates (intact) (*n* = 3, mean ± SEM). N.D. denotes not detected. h) Representative fluorescence images of brain tissue sections showing brain penetration of ASO@D‐Cb. Green: tumor (U87MG‐FLuc‐GFP), blue: nuclei, red: ASO or ASO@D‐Cb, magnification: 200×, scale bar: 100 µm. i) Relative distribution levels of ASO and ASO@D‐Cb in tumor and normal regions estimated by image analysis of the sectioned‐brain tissue using ImageJ) (*n* = 5, mean ± SEM).

Having determined that the protein corona recognizable by receptors mediating BBB penetration is a key factor in the brain distribution and GBM tumor accumulation of D‐Cb, we investigated the potential of D‐Cb as a carrier for brain delivery of ASO to treat GBM. As a model therapeutic ASO, we selected an ASO sequence targeting polo‐like kinase 1 (PLK1) mRNA, a potential target for the treatment of GBM.^[^ ^36^, ^37^ ^]^ The loading ratio between ASO and D‐Cb was designed to be 1:1. ASO was loaded in D‐Cb by adding the ASO sequence to the 3′‐end of the S6 strand (**Figure** **6**a; Table S6, Supporting Information). According to gel analysis (Figure S12, Supporting Information), the loading efficiency of ASO exceeds 90%. The ASO used is a gapmer with 5′‐ and 3′‐ends modified with phosphorothioates, providing significant nuclease resistance in plasma. The Cb structure demonstrated stability in mouse serum for the circulation time (2 h) used for brain biodistribution (Figures 3a and 5b). Given the stability of both ASO and D‐Cb, ASO@D‐Cb is expected to remain stable in plasma for the time required for brain distribution. The hydrodynamic size of ASO@D‐Cb was 8.32 ± 0.19 nm, slightly larger than that of D‐Cb (Figure S13, Supporting Information).

The gene silencing effect of ASO@D‐Cb was initially evaluated in glioblastoma cells (U87MG), where D‐Cb enhanced cellular uptake of ASO approximately by 10‐fold, as estimated by fluorescence microscopy and flow cytometry (Figure 6b,c). Treatment with ASO@D‐Cb reduced PLK1 mRNA in U87MG by ≈50% compared to phosphate buffered saline (PBS)‐treated cells (negative control), as determined by quantitative reverse‐transcriptase PCR (qRT‐PCR) (Figure 6d). The gene silencing effect by ASO@D‐Cb was comparable to that of the positive control, lipofectamine‐delivered ASO. D‐Cb loaded with a scrambled ASO sequence (ASO‐SC@D‐Cb) did not exhibit significant target gene silencing activity, indicating that gene silencing by ASO@D‐Cb was achieved in a target sequence‐specific manner. The downregulation of PLK1 mRNA subsequently led to PLK1 protein level in the cells, as observed by western blot analysis (Figure 6d; Figure S14, Supporting Information).

After confirming the cellular activity of ASO@D‐Cb, we intravenously administered Cy5.5‐labeled ASO@D‐Cb into orthotopic GBM‐bearing mice and monitored fluorescence intensity around the head (Figure 6e). Both ASO and ASO@D‐Cb were detected in the head region 10 min after injection. After 1–2 h, the Cy5.5 intensity in the head area peaked and then slowly decreased. The brain was harvested and imaged using the Cy5.5 label on ASO at 2 and 24 h post injection (Figure 6f; Figure S15, Supporting Information). ASO@D‐Cb showed a higher brain distribution level than ASO alone. PAGE analysis of brain lysates revealed that ASO delivered by D‐Cb was mostly in its intact form (Figure 6g; Figure S16, Supporting Information), whereas no intact ASO was detected when delivered without D‐Cb, indicating that ASO alone was unable to reach the brain. The brain distribution of ASO@D‐Cb in GBM mice was not significantly different from that in healthy BALB/c mice (Figure 6g; Figures S17 and S18, Supporting Information). Fluorescence microscopy of brain sections showed that the majority of ASO@D‐Cb was distributed within the U87MG‐FLuc‐GFP tumor region, which was indicated by the green fluorescence signal by GFP expression (Figure 6h,i). ASO uptake in the brain tissue section was negligible. This indicates that tumor distribution of D‐Cb was not significantly affected by the loading of ASO.

### 2.6 Therapeutic Efficacy of Systemically Administered ASO@D‐Cb for GBM Treatment

**Figure 7:**
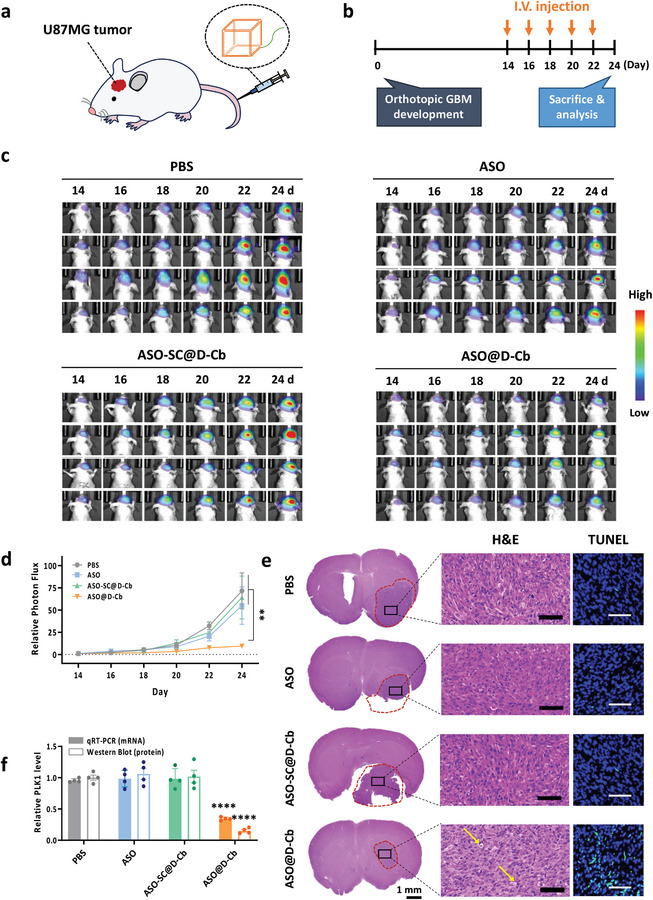
Therapeutic efficacy of ASO@D‐Cb for in vivo treatment of GBM. a) Schematic illustration showing ASO@D‐Cb intravenously injected into U87MG tumor‐bearing mice to treat GBM. b) The schedule for preparation of the GBM mouse model and ASO treatments for downregulation of the in vivo PLK1 level. c) Luminescence images of BALB/c nude mice bearing orthotopic U87MG‐FLuc‐GFP tumor after treatment with PBS, free ASO, ASO‐SC@D‐Cb or ASO@D‐Cb. The mice were intravenously injected at a dose of 400 pmol ASO per mouse (20 nmol kg^−1^) on days 14, 16, 18, 20, and 22 post‐tumor implantation. d) Luminescence levels of mice estimated in the IVIS system (*n* = 4, mean ± SEM, ^**^ *p* < 0.01). e) Histological images of H&E‐stained brain tissue sections from PBS, ASO, ASO‐SC@D‐Cb or ASO@D‐Cb treated mice. The tumor regions of interest in the brain sections, indicated by black boxes, were magnified. Scale bars of the magnified images indicate 100 µm. Red dashed lines indicate the tumor region and yellow arrows indicate chromatin shrinkage in nuclei damage. The apoptotic tumor cells (green) in brain sections were analyzed by TUNEL assay (magnification: 200×, scale bar: 100 µm). f) In vivo potency of ASO in GBM mice estimated using qRT‐PCR analysis of PLK1 mRNA levels and western blotting of PLK1 protein levels in brain tissue lysates. (*n* = 4, mean ± SEM, ^****^ *p* < 0.0001 vs PBS‐treated group).

To evaluate the in vivo therapeutic efficacy of ASO@D‐Cb, mice were serially administered with 5 doses at 2‐day intervals (**Figure** **7**a,b). Using U87MG‐FLuc‐GFP cells to generate the GBM model allowed us to monitor in vivo tumor size using luminescence (Figure 7c). We also prepared three control groups to evaluate the therapeutic efficacy of ASO@D‐Cb. One group of mice was injected with PBS as a negative control. Another group was injected with ASO to determine if the delivery potential of D‐Cb is critical for the in vivo efficacy of ASO. The third group was injected with ASO‐SC@D‐Cb to assess whether the therapeutic effect was due to the ASO sequence or the D‐Cb in ASO@D‐Cb. Tumor growth in mice treated with ASO@D‐Cb was inhibited by ≈85% compared to untreated control mice (Figure 7d). In contrast, free ASO failed to inhibit tumor growth, demonstrating the need for delivery with D‐Cb to achieve significant therapeutic potency for GBM. Treatment with ASO‐SC@D‐Cb did not exhibit significant potency for GBM treatment, indicating that the potency of ASO@D‐Cb resulted from ASO sequences specific to the target mRNA. This also suggests that the therapeutic effect of ASO@D‐Cb is attributed to the ASO part rather than the D‐Cb part. Hematoxylin and eosin (H&E) staining of tumor tissue sections revealed damaged areas only after treatment with ASO@D‐Cb (Figure 7e). No significant tissue damage was observed in other major organs (Figure S19, Supporting Information). Apoptotic cell death induced by ASO@D‐Cb in the brain was also confirmed by transferase‐mediated nick end labeling (TUNEL) assay (Figure 7e). Accordingly, PLK1 mRNA and protein levels in tumor lysates were downregulated only by ASO@D‐Cb, as determined by qRT‐PCR and western blot analysis, respectively (Figure 7f; Figure S20, Supporting Information). These results suggest that the antitumor effect of ASO@D‐Cb was mediated by apoptotic cell death resulting from PLK1 downregulation.^[^ ^38^ ^]^ This therapeutic potency of ASO@D‐Cb overall alleviated the weight loss of mice by the GBM toxicity (Figure S21, Supporting Information).

While D‐Cb significantly enhances the systemic brain delivery of ASO for the treatment of GBM, there is still room for improvement in the brain distribution level of D‐Cb. The protein corona on D‐Cb, which appears to contain proteins contributing to BBB penetration, could be further optimized. One possible approach as a follow‐up study in the future could be the conjugation of a moiety that promotes a protein corona enriched with LRP ligands, LDL, and transferrin, thereby increasing the brain distribution level of D‐Cb.

## 3 Conclusion

In this study, we assembled six wireframe DNA nanostructures by combining two sugar backbones (D‐DNA and L‐DNA) with three shapes (Td, Tp, and Cb) to develop a brain delivery platform based on the protein corona‐assisted way. Ex vivo brain imaging and brain lysate analysis revealed that D‐Cb had the highest distribution potential. The brain distribution level of D‐Cb, even without brain‐targeting ligands, was comparable to that of other previous nanoparticles conjugated with brain‐targeting ligands. Proteomic analysis revealed that the protein corona, formed by serum proteins on the DNA nanostructures, was the key to the brain distribution potential of D‐Cb. This protein corona contains ligands for the main endocytic receptors that facilitate BBB penetration. As these endocytic receptors are also abundantly expressed in the glioblastoma multiforme (GBM) cells such as U87MG, an even higher brain distribution of D‐Cb as well as tumor accumulation was observed in GBM‐bearing mice. D‐Cb was subsequently employed as a carrier for brain delivery of ASO to downregulate PLK1 mRNA and treat GBM in mice. While free ASO failed to reach the brain, ASO loaded onto D‐Cb was efficiently delivered to the tumor region in the brain, where it downregulated the target gene and exerted an antitumor effect on GBM. We anticipate that D‐Cb can serve as a viable platform based on protein corona formation for systemic brain delivery of therapeutic oligonucleotides.

## 4 Experimental Section

### Statistical Analysis

The data were presented as the mean ± standard error of the mean (SEM). To assess the significance of observed differences, statistical analysis was conducted through one‐way analysis of variance (ANOVA) using Tukey's multiple comparison test in GraphPad Prism software. In this analysis, data with ^*^ *p* < 0.05, ^**^ *p* < 0.01, ^***^ *p* < 0.001, and ^****^ *p* < 0.0001 were considered statistically significant.

### Oligonucleotide Synthesis

Oligonucleotides were synthesized at a 1 µmol‐scale using the Mermaid‐4 DNA/RNA synthesizer (Bioautomation, MN, USA) with conventional phosphoramidite chemistry. After synthesis, the oligonucleotides were cleaved from the solid support (CPG) and deprotected in 33% aqueous ammonia at 55 °C for 17 h. Subsequent purification was achieved through denaturing polyacrylamide gel electrophoresis (PAGE), followed by ethanol precipitation. The purified strands were characterized via electronspray ionization mass spectrometry (ESI‐MS) analysis, conducted by Novatia Inc. (PA, USA).

### Preparation of Self‐Assembled DNA Nanostructures

The solution containing oligonucleotides (1 µM, Table S1, Supporting Information) in TM buffer (5 mM MgCl_2,_ 10 mM Tris‐HCl, pH 8.3) was heated to 95 °C for 10 min and slowly cooled down to 4 °C for 24 h. The self‐assembled structures were verified on native PAGE (10%) in 0.5 × TBE (tris‐borate‐EDTA) buffer. The bands were visualized using 3′‐FAM incorporated in the S1 strand. The gels were imaged using the iBrightFL1000 system (Thermo Fisher Scientific, MA, USA).

### Preparation of Orthotopic GBM Mice

All animal experiments were approved by the Institutional Animal Care and Use Committee at Gachon University (Approval number: LCDI‐2022‐0076) and at the Korea Institute of Science and Technology (Approval Number: IACUC‐2022‐047‐2). The U87MG‐fLuc‐GFP cell line was kindly provided by Dr. Kwang Il Kim from the Korea Institute of Radiological and Medical Sciences. Orthotopic GBM‐xenografted mice were prepared using U87MG‐fLuc‐GFP cells following a previously established protocol.^[^ ^39^ ^]^ Briefly, male athymic BALB/c nude mice (5 weeks old, 20–30 g) were placed on a stereotactic device (Harvard Apparatus, MA, USA) using ear bars under 1–1.5% isoflurane inhalation anesthesia. U87MG‐FLuc‐GFP cells (1 × 10^5^ cells) suspended in 2 µL of PBS were injected into the right striatum at a flow rate of 0.5 µL min^−1^ through a tiny hole on the skull at coordinates 2.0 mm laterally, 0.2 mm anteriorly, and 3.2 mm ventrally from the bregma. Brain tumor growth was monitored by bioluminescence intensity (BLI).

## Conflict of Interest

The authors have no conflicts of interest to disclose.

## Author Contributions

K.‐R.K. and J.H.K. contributed equally to this work. K.‐R.K. and H.B.D.T. prepared and characterized DNA nanostructures; K.‐R.K. and J.H.K. performed experiments to analyze cellular and in vivo properties of DNA nanostructures; J.H. K. prepared the orthotopic GBM mouse model; J.H.B. and J.E.L. analyzed the proteome of the protein corona; C.M. provided AFM images of DNA nanostructures; D.‐R.A. and Y.T.K. designed the study and wrote the manuscript.

## Supporting information

Supporting Information

## Data Availability

The data that support the findings of this study are available in the supplementary material of this article.
