# Supplementary material for: Systemic Brain Delivery of Oligonucleotide Therapeutics Enhanced by Protein Corona‐Assisted DNA Cubes
Source: Small Methods. 2024 Aug 2;9(6):2400902. doi: 10.1002/smtd.202400902 (PMC12182886; doi:10.1002/smtd.202400902)
Supplement: Supplementary file 1 — Supporting Information [file SMTD-9-2400902-s001.docx]

# Supporting Information

**Systemic brain delivery of oligonucleotide therapeutics enhanced by protein corona-assisted DNA cubes**

Kyoung-Ran Kim^a,†^, Ji Hee Kang^b,†^, Hien Bao Dieu Thai^a^, Ji Hyun Back^a^, Chengde Mao^c^, Ji Eun Lee^a^, Young Tag Ko^b,*^, and Dae-Ro Ahn^a,d,*^

*^a^Chemical and Biological Integrative Research Center, Korea Institute of Science and Technology (KIST), Hwarangno 14-gil 5, Seongbuk-gu, Seoul 02792, Korea*

*^b^College of Pharmacy, Gachon University, 191 Hambakmoe-ro, Incheon, 406-799, Republic of Korea*

*^c^Department of Chemistry, Purdue University, West Lafayette, Indiana 47907, USA*

*^d^Division of Biomedical Science and Technology, KIST School, University of Science and Technology (UST), Hwarangno 14-gil 5, Seongbuk-gu, Seoul 02792, Korea*

^†^These authors contributed equally to this work

^*^Corresponding E-mails: drahn@kist.re.kr (D.-R. Ahn); youngtakko@gachon.ac.kr (Y.T. Ko)

**Materials**

All quantitative RT-PCR (qRT-PCR) primers and phosphoramidites required for D-DNA synthesis were purchased from Bioneer (Daejeon, Korea). The phosphoramidites for L-DNA synthesis and the CPG for 3' modifications (3'-FAM and 3'-NH_2_) were purchased from Glen Research (Sterling, VA, USA). Streptavidin-coated magnetic beads (Dynabeads™ MyOne Streptavidin™ T1) and the SYBR green Master Mix used for qRT-PCR were obtained from Thermo Fisher Scientific (Waltham, MA, USA). The BALB/c mice used in the study, specifically male individuals aged 5 weeks, were provided by Orient Bio Inc. (Seongnam, Korea). Various additional buffers, organic solvents, and chemical reagents essential for the experimental procedures were purchased from Biosesang (Seongnam, Korea), Samchun Chemicals (Seoul, Korea), and Sigma Aldrich (Missouri, USA), respectively. RNeasy Mini kit was obtained from Qiagen (Hilden Germany) while the cDNA synthesis kit was purchased from Enzynomics (Daejeon, Korea). The antibodies employed in western blotting and flow cytometry were purchased from Cell Signaling Technology (Danvers, MA, USA), Santa Cruz Biotechnology (Dallas, TX, USA), Abcam (Cambridge, UK), R&D Systems (Minneapolis, MN, USA), Bethyl Laboratories Inc. (Montgomery, TX, USA), and Biolegend (San Diego, CA, USA).

**Preparation of Cy5.5-labeled D-Cb and L-Cb**

To prepare Cy5.5-labeled Cb structures for *in vivo* experiments, the 3’-Cy5.5-conjugated S1 strand was used for the self-assembly. For Cy5.5 conjugation, 3’-amine-modified S1 (3’NH_2_-S1) was reacted with 10 equiv. of NHS-ester Cy5.5 (Lumiprobe, MD, USA) in 0.1 M sodium bicarbonate buffer (pH 8.3 - 8.5) at 4°C overnight. Cy5.5-labeld S1 was purified using ethanol precipitation.

**Dynamic light scattering (DLS)**

The hydrodynamic sizes of DNA nanostructures (250 nM in TM buffer) were determined by dynamic light scattering (DLS) employing a Zetasizer instrument (Malvern Instruments, Worcestershire, UK).

**Atomic force microscopy (AFM)**

DNA nanostructures assembled within the concentration range of 50 - 100 nM were suitably diluted to 20 nM in 1X TAE-Mg buffer (composed of 50 mM Tris-acetate, 2 mM EDTA, 12.5 mM MgCl_2_). This diluted solution was then combined with an equal volume of 1X TAE-Mg containing 10 mM NiCl_2_. The mixture was deposited onto mica surfaces pre-treated with NiCl_2_ and subsequently incubated for 1 min at room temperature. Following this, the samples were imaged using non-contact mode on an atomic force microscopy (AFM) platform (ScanAsyst Multimode, Bruker, USA), employing ScanAsyst-Fluid+ tips (Bruker, USA) in a fluidic environment.

**Serum stability of DNA nanostructures**

DNA nanostructures (FAM-labeled, 1 µM) were incubated in 50% mouse serum for 0, 1, 4, 7, and 24 h at 37℃. At each time point, the enzymatic reaction was quenched by adding loading buffer (95% formamide in 0.5 M EDTA) followed by a heat treatment step at 95℃ for 10 min. Subsequently, the samples were subjected to agarose (1%) gel electrophoresis, run in 0.5X TBE buffer under 120 V for 1 hour. The bands were imaged using the iBright FL1000 imaging system.

**Pull-down of serum proteins with D-Cb and L-Cb**

Streptavidin-coated magnetic beads or biotinylated D-Cb and L-Cb (1 μM) immobilized onto the streptavidin-coated magnetic beads (20 μL) were washed thrice with 1X phosphate-buffered saline (PBS) (100 μL) and then incubated in mouse serum (50 μL, Sigma-Aldrich) in a binding buffer (50 μL, 2X PBS) at 37℃ for 1 h. The supernatant was removed. The beads were washed thrice with 1X PBS. The beads were resuspended in loading buffer (50 mM Tris-HCl, pH 6.8, 2% SDS, 6% v/v glycerol, 2 mM DTT, and 0.01% w/v bromophenol blue) and incubated at 95℃ for 10 min. The mixture was subsequently analyzed on 5 - 12% SDS-PAGE. After stained with Coomassie Blue, the gel was imaged with gel images using the iBrightFL1000 system.

**Proteomic analysis**

Proteins that were, respectively, pulled down from three independent binding experiments of bead, D-Cb, and L-Cb, were separated based on the molecular weight using 5-12% SDS-PAGE. After the gel was stained with Coomassie Blue, the stained gel for each lane of the samples that were pulled down from three types of beads was divided into eight slices and the proteins contained in each gel slice were subjected to tryptic digestion. The proteins were first reduced with 10 mM DTT in 25 mM NH_4_HCO_3_ for 1 h at 56℃ and alkylated with 55 mM iodoacetamide in 25 mM NH_4_HCO_3_ for 1 h at 25℃ in the dark followed by trypsin digestion overnight. Peptides were then extracted with 67% acetonitrile (ACN)/5% formic acid (FA) in water and dried in a miVAC vacuum concentrator (Genevac Ltd., Ipswich, UK). Then, they were resuspended with 20 μL of 0.4% acetic acid. For mass spectral analysis, 13.5 µL of each sample was injected into a reversed-phase Magic C18AQ column (15 cm × 75 µm) on an Eksigent MDLC system (Eksigent Technologies, CA, USA). The three replicates of each type of samples were analyzed using mass spectrometry (MS); thus, they represented the technical replicates of all processing steps including pulled down assay, in-gel digestion, and MS analysis. The operating flow rate was 350 nL/min, with the following gradient conditions: 0 min 100% buffer A (100% water with 0.1% FA) and 0% buffer B (100% ACN with 0.1% FA), 0-5 min 0-8% B, 5-85 min 8-30% B, 85-90 min 30-70% B, 90-100 min 70% B, 100-110 min 70-2% B, and 100-120 min 2% B. The nano HPLC system was coupled to an LTQ XL-Orbitrap mass spectrometer (Thermo Fisher Scientific, MA, USA). The spray voltage was set to 2.5 kV and the temperature of the heated capillary was set to 250°C. Survey full-scan mass spectrometry (MS) spectra (300–2000 m/z) were acquired with 1 micro scan at a resolution of 60,000, allowing preview mode for precursor selection and charge-state determination. Tandem mass (MS/MS) spectra for the ten most intense ions were acquired in the ion trap with the following options: isolation width, 2 m/z; normalized collision energy, 35%; dynamic exclusion duration, 30 s. Precursors with +1 charge and unassigned charge states were discarded during data-dependent acquisition. Each LC-MS/MS file was searched against the SwissProt mouse database (June 2022) with 17132 entries using Proteome Discoverer software (version 2.4, Thermo Fisher Scientific, Bremen, Germany). The search criteria were set to a mass tolerance of 15 ppm for MS data and 0.5 Da for MS/MS data with fixed modification of carbamidomethylation of cysteine (+57.021 Da) and variable modification of methionine oxidation (+15.995 Da). The false discovery rate (FDR) was set at 0.01 for the identification of peptides and proteins. All proteins were identified by two or more unique peptides. For label-free quantitative analysis obtained from three replicates of each type of samples, normalized abundance values of proteins in each sample were obtained from peak area normalized by total peptides using Minora algorithm-based label-free quantification in Proteome Discoverer 2.4. Statistical analysis of the normalized abundance values obtained from the label-free quantification was performed using Perseus software (1.6.14.0) [1]. Normalized abundance values were log-transformed, and then missing values were replaced using values computed from the normal distribution with a width of 0.3 and a downshift of 1.8. Proteins exhibiting statistical significance among samples pulled down from bead, D-Cb, and L-Cb were obtained by ANOVA comparison of the log_2_(normalized abundance) values obtained from the three replicates of each type of sample. *P*-value < 0.05 was considered statistically significant. For hierarchical clustering of proteins showing statistically significant changes (*P*-value < 0.05) among samples pulled down with bead, D-Cb, and L-Cb, the abundance values were first normalized using z-score and then clustering of both columns and rows was pursued based on Euclidean distance using the average linkage method using Perseus (1.6.14.0).

**Cellular uptake experiments**

U87MG cells (Korean Cell Line Bank, Seoul, Korea) were seeded at a density of 5 × 10^4^ cells per well in a 24-well plate. After 24 h, the cells were washed twice with PBS and were subsequently treated with Cy5.5-labeled ASO or ASO@D-Cb (50 nM) in 10% fetal bovine serum (FBS)-supplemented Dulbecco's Modified Eagle Medium (DMEM; Welgene, Gyeongsan, Korea) at 37℃ within a 5% CO_2_ environment. After 6 h, the cells were washed with three times, suspended in ice-cold PBS (500 μL), and analyzed by flow cytometry (Guava, Millipore, Massachusetts, USA). A minimum of 10,000 cells were examined in triplicate for each sample. For confocal microscopic imaging, cells (2.5 × 10^4^) were cultured on glass-bottomed dishes and treated with Cy5.5-labeled ASO or ASO@D-Cb (50 nM) for 6 h. Subsequently, the cells were stained with Hoechst 34580 (1 μg/mL, Thermo Fisher Scientific) for 5 min and washed with PBS twice. The cellular fluorescence signals were visualized using a confocal microscopy (LSM 800, Carl Zeiss, Jena, Germany).

To elucidate receptor-mediated cellular uptake mechanisms, bEnd.3 cells or U87MG cell were pretreated with 10 µg/mL of antibodies (anti-TfR antibody, anti-LRP1 antibody, anti-LRP2 antibody, or anti-LDLR antibody) for 1 h before treated with FAM-labeled D-Cb or L-Cb for 6 h. Then, the uptake efficiency was analyzed by flow cytometry.

***In vitro* BBB permeability assay**

To evaluate the *in vitro* BBB penetration of DNA nanostructures, we carried out the penetration study using an *in vitro* BBB monolayer model. Brain endothelial bEnd.3 cells were seeded at a density of 3.0 x 10^4^ cells on Transwell permeable inserts (0.4 μm, 6.4 mm in diameter; Falcon^®^, Corning, NY, USA) with 10% FBS-containing DMEM and incubated at 37 ℃ in a 5% CO_2_ atmosphere for a week. The integrity of the BBB model was confirmed by transendothelial electrical resistance (TEER) measurements using EVOM2 (World Precision Instruments, Sarasota, FL, USA). When the TEER value was above 250 Ω, the Transwell insert was washed with PBS, and 200 μL of complete medium containing FAM-labeled DNA nanostructures (1 μM) was added to the apical side of the culture insert. The basolateral compartment of the inset was filled with 500 μL complete medium. The complete medium with DNA nanostructures was collected from the basolateral side, and the basolateral part was refilled with fresh complete medium at pre-determined times (0.5, 1, 2, and 4 h). The fluorescence intensity of collected samples was measured with a fluorescence spectrometer (Multi-detector microplate reader Synergy H1 Hybrid, BioTek). After quantification of the DNA nanostructures in the samples, the apparent permeability (P_app_), indicating the *in vitro* BBB permeability, was calculated using the following equation:


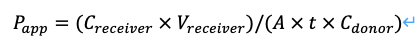


where C_donor_, C_receiver_, V_receiver_, A, and t are the concentration of DNA nanostructures in the donor (apical side), the concentration of DNA nanostructures in the receiver (basolater side), receiver volume, membrane area, and incubation time, respectively.

To investigate the receptor-mediated BBB transcytosis efficiency of D-Cb, a transpenetration study with TfR, LRP1, LRP2, LDLR antibodies was performed using an *in vitro* BBB monolayer model. On the day of the assay, the cells in the insert were pre-treated with each antibody in serum-containing medium (10 μg/mL) for 1 h at 37 ℃. After 1 h incubation, complete medium containing FAM-labeled D-Cb (1 μM) was added to the apical side. Sample collection from the basolateral side at pre-determined times and BBB permeability calculation were processed as described above.

***In vitro* blood-tumor barrier (BTB) penetration assay**

*In vitro* U87MG-cellular uptake analysis after BBB penetration (BTB penetration assay) was performed using an *in vitro* BBB co-culture model. Briefly, bEnd.3 cells were seeded at a density of 3.0 x 10^4^ cells into the Transwell permeable inserts (apical chambers) and incubated for 5 days. Then U87MG cells were seeded at a density of 3.0 x 10^4^ on the coverslip in the basolateral chambers. After 2 days, 200 μL of complete medium containing FAM-labeled DNA nanostructures (1 μM) was added to the apical side of the culture insert. The basolateral compartment of the inset was filled with 500 μL complete medium. After 4 h of incubation at 37°C, the U87MG cells in the basolateral chambers were washed four time with PBS, stained with DAPI for 5 min, and then fixed using 2% PFA solution. The coverslips were mounted with a mounting solution on the slide glass and then observed fluorescence intensity under a confocal microscopy (A1Plus, Nikon). The relative fluorescence intensity was determined by normalizing the intensity ratio FAM(Cb)/DAPI(nuclei).

***In vivo*** **imaging**

Cy5.5-labeled DNA nanostructures or ASO (2 μM, 200 μL) were intravenously administered to healthy BALB/c or GBM mice. *In vivo* fluorescence images were acquired using the IVIS Imaging Spectrum System, utilizing an emission wavelength of 700 nm and an excitation wavelength of 640 nm. The data were subsequently analyzed with the IVIS Living Imaging 3.0 software. For *ex vivo* imaging, at 2 h post injection, the mice were euthanized, and the organs were extracted and imaged *via* the IVIS system.

**Biodistribution of D-Cb and L-Cb**

At 2 h post injection of D-Cb or L-Cb, the mice (BALB/c or GBM) were transcranially perfused with 10 mM potassium PBS at a flow rate of 1 mL/min (1 mL) to remove the nanostructures in blood, and the brain was harvested. To estimate brain distribution levels (injected dose per gram of tissue: ID%/g) of D-Cb and L-Cb labeled with FAM or Cy5.5, the excised brain tissues homogenized under cryogenic conditions and were subsequently lysed in RIPA buffer. The lysate centrifuged (12,000 rpm, 10 minutes, 4°C), and fluorescence intensity of the supernatant from each sample was measured using a plate reader (Victor Nivo, Perkin Elmer, USA) with a specific filter set (Ex 640 nm Em 685 nm) for quantification of total amount of fluorescence-labeled structures. For quantification of intact oligonucleotides, the brain lysates were analyzed using 10% denaturing PAGE (7 M urea). Reference quantities of oligonucleotides (Cy5.5-labeled; 0.032, 0.64, 0.125, 0.25, and 0.5 pmol) were collaterally loaded onto the gel. The quantity of intact oligonucleotides present was estimated based on the intensity of the gel bands, which were subsequently quantified by the ImageJ software. Using the determined amount of intact oligonucleotides within each lysate, the amount of the intact D-Cb and L-Cb in the brain was calculated, accounting for the total lysate volume and the tissue weight.

**Brain section imaging**The brains of GBM mice were excised 2 h after intravenous injection of Cy5.5-labeled D- or L-Cb. The harvested brains were embedded with optimal cutting temperature (OCT) compound (Leica Biosystems, Germany) and frozen in deep freezer. The frozen tissue blocks were cut in 15 μm by Lab Core Incorporation (Seoul, Korea). The section slides were washed with PBS, mounted with DAPI containing-mounting solution (Abcam, UK) and imaged using confocal microscopy (LSM 800).

**ESI-MS detection of intact oligonucleotides in brain lysates**

Biotinylated D-Cb (2 µM, 200 µL) was administered intravenously to BALB/c mice. After 2 h, cerebral tissues were collected and subsequently homogenized under cryogenic conditions and lysed in RIPA buffer. The lysed tissue samples were then centrifuged at 12,000 g for 10 min at 4 °C, and the supernatant was collected as the brain tissue lysate. Streptavidin-coated magnetic beads (30 µL) were washed with the buffer (0.5 mM EDTA, 1 M NaCl, 5 mM Tris-HCl, pH 7.5) three times and incubated with the tissue lysate at 37℃ for 2 h. The beads washed with PBS and 50 mM NaOH. Biotinylated S5 strands were eluted by incubation the beads with D-biotin (1 mM) in distilled water at 95℃ for 20 min. D-biotin was removed by filtration of the eluted solution using a G25 filtration column. The filtrate was analyzed using electrospray ionization mass spectrometry (ESI-MS), which was conducted by Novatia Inc. (Pennsylvania, USA).

**Systemic brain delivery of ASO@D-Cb in orthotopic GBM mice.**

Orthotopic GBM-xenografted mice were intravenously administered with Cy5.5-labeled ASO@ D-Cb (200 μL, 400 pmol/mouse, 20 nmol/kg) through the tail vein. After 2 h, the GBM-bearing mice were transcranially perfused with 10 mM potassium PBS at a flow rate of 1 mL/min (50 mL) and then fixed by 4% (v/v) paraformaldehyde (PFA) solution at a flow rate of 2 mL/min (50 mL). The brains were collected, divided into 4 mm coronal slices, and then post-fixed with a 4% (v/v) PFA solution overnight at 4 °C. The brain coronal slices were sectioned with 40 μm thickness using a vibratome (Leica V1000S, Germany) and the sections were stained by DAPI (Life Technologies). The stained tumor tissue slides were observed by a laser scanning confocal microscopy (LSCM, A1Plus, Nikon, Tokyo, Japan). The fluorescence intensity in the images was quantitatively analyzed using Nikon NIS-E image analysis software.

**Biodistribution of ASO@D-Cb in orthotopic GBM mice**

Orthotopic GBM mice were intravenously administered with Cy5.5-labeled ASO@D-Cb (200 μL, 400 pmol/mouse, 20 nmol/kg) through the tail vein. Whole-body fluorescence images were obtained at the predefined time points within 24 h following intravenous administration using an IVIS optical imaging system with a long wavelength emission filter (675–730 nm) (Ami HT imaging system, Spectral Instruments Imaging, Tucson, AZ, USA).

***In vivo* efficacy of ASO@ D-Cb for treatment of GBM**

The orthotopic GBM mice were weighed and randomly divided into four groups (n = 4), and each group was intravenously injected with PBS (200 μL), ASO, ASO-SC@D-Cb, or ASO@D-Cb (400 pmol ASO/mouse) *via* the tail vein every two days for 10 days. The bioluminescence signal from the brain tumor, generated by intraperitoneal injection of 150 mg/kg D-luciferin, was imaged on every injection day before treatment. IVIS (Ami HT imaging system) was used to repeatedly acquire the images over 10 min at intervals of 2 min. The bioluminescence intensity was analyzed using Aura imaging software (Spectral Instruments Imaging). Mice body weights monitored every 2 days during ASO@D-Cb treatment. After the mice were sacrificed on day 24, the major organs were collected, washed, and fixed with 4% (v/v) PFA solution overnight at 4 °C. The fixed tissues were embedded in paraffin and sectioned, mounted on the glass slides. The sections were stained with hematoxylin and eosin (H&E) and subject to the transferase-mediated nick end labeling (TUNEL) assay.

**Western blot**

Cell lysates (10 μg) or brain lysates (50 μg) were separated by 12% sodium dodecyl sulfate polyacrylamide gel electrophoresis (SDS-PAGE). After the electrophoresis, the proteins in the SDS-PAGE gel were transferred onto a polyvinylidene fluoride (PVDF) membrane. The membrane was incubated with TBST (10mM Tris-HCl, pH 8.0, 150 mM NaCl, 0.05% Tween 20) containing 5% skim milk at room temperature for 1 h and then washed with TBST three times. The membrane was incubated with primary antibodies, specifically anti-PLK1 antibody (dilution 1:1000, Santa Cruz Biotechnology) and anti-GAPDH antibody (dilution 1:1000, Cell Signaling Technology) in TBST containing 5% BSA overnight at 4℃. After washing unbound antibodies with TBST three times, the membrane was incubated with a horseradish peroxidase (HRP)-conjugated secondary antibody (dilution 1:10,000, Santa Cruz Biotechnology) in TBST containing 5% skim milk at room temperature for 1 h. After a rigorous washing with TBST three times, protein bands were visualized utilizing the Super Signal™ West Pico Chemiluminescent substrate (Thermo Fisher Scientific). The resulting images were captured using the iBright FL1000 imaging system.

**Quantitative reverse transcription polymerase chain reaction (qRT-PCR)**

U87MG cells were seeded on a 12-well plate (2 × 10^5^ cells/well). Cells were treated with ASO, ASO@D-Cb, ASO-SC@D-Cb, PBS, D-Cb, and ASO/Lipofectamine RNAiMax in 10% FBS-supplemented DMEM. After 24 h, the cellular RNA was extracted using RNeasy Mini kit (Qiagen). The RNA concentration was quantified by Nanodrop system (Thermo Fisher Scientific). The cDNA of the cellular RNA (2 μg) was prepared by random hexamer-primed reverse transcription. The cDNA was mixed with 2X SYBR Green Master Mix (Thermo Fisher Scientific) and PLK1 or GAPDH primers (primer sequences were described in Table S7). PCR of the cDNA was performed using the StepOnePlus real-time PCR system (Applied Biosystems). The relative amount of PLK1 transcripts was normalized based on the amount of GAPDH transcripts, calculated with the 2-∆∆Ct method. For the estimation of PLK1 mRNA levels in the brain lysates, total RNA was extracted from homogenized brain tissue and analyzed using the procedure described above.

**Figure S1.** 10% non-denaturing PAGE showing assembly of DNA nanostructures. M denotes an 100-bp size marker.


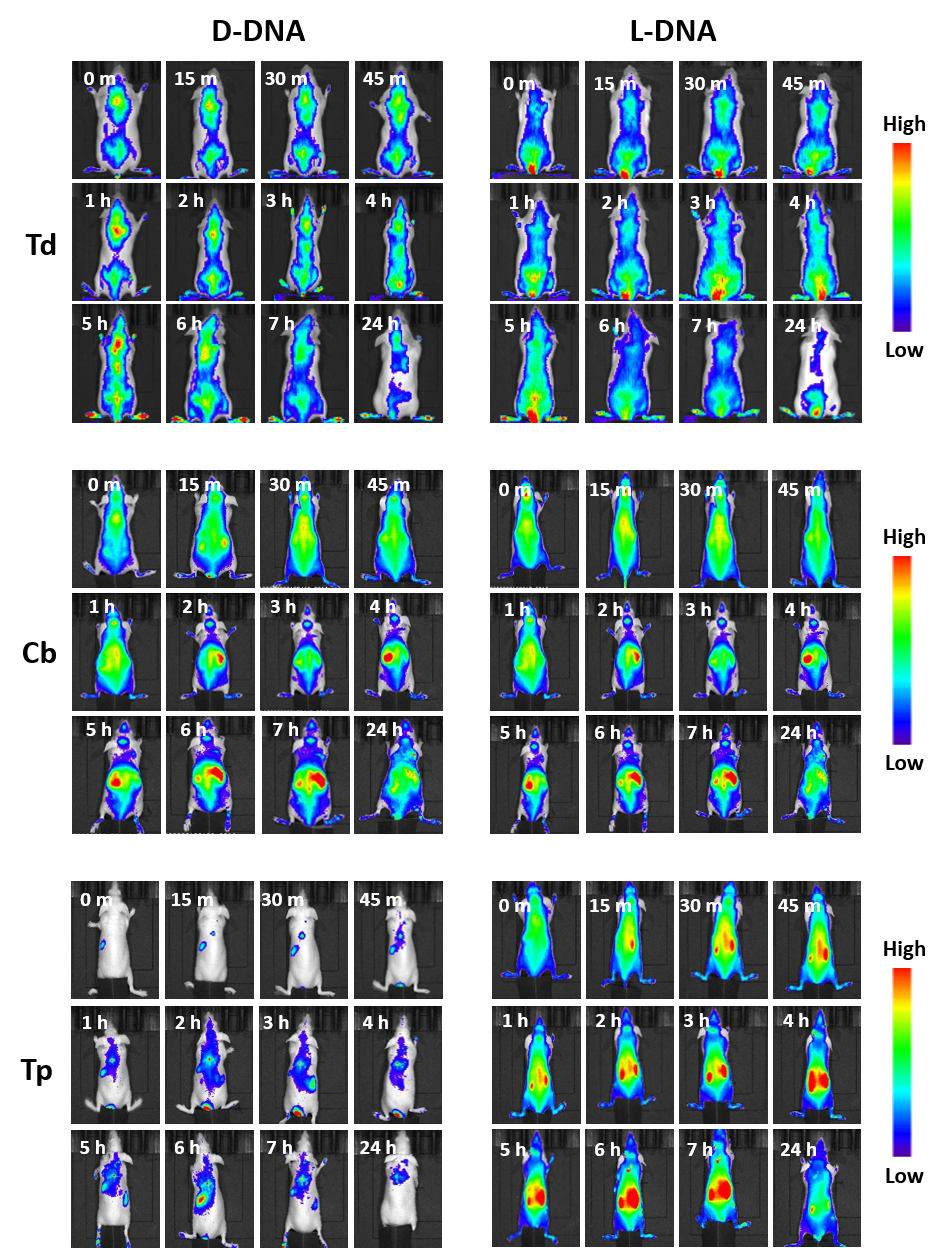


**Figure S2.** Time dependent *in vivo* biodistribution of DNA nanostructures in BALB/c mice after intravenous injection of Cy5.5-labeled structures (2 µM, 200 µL).


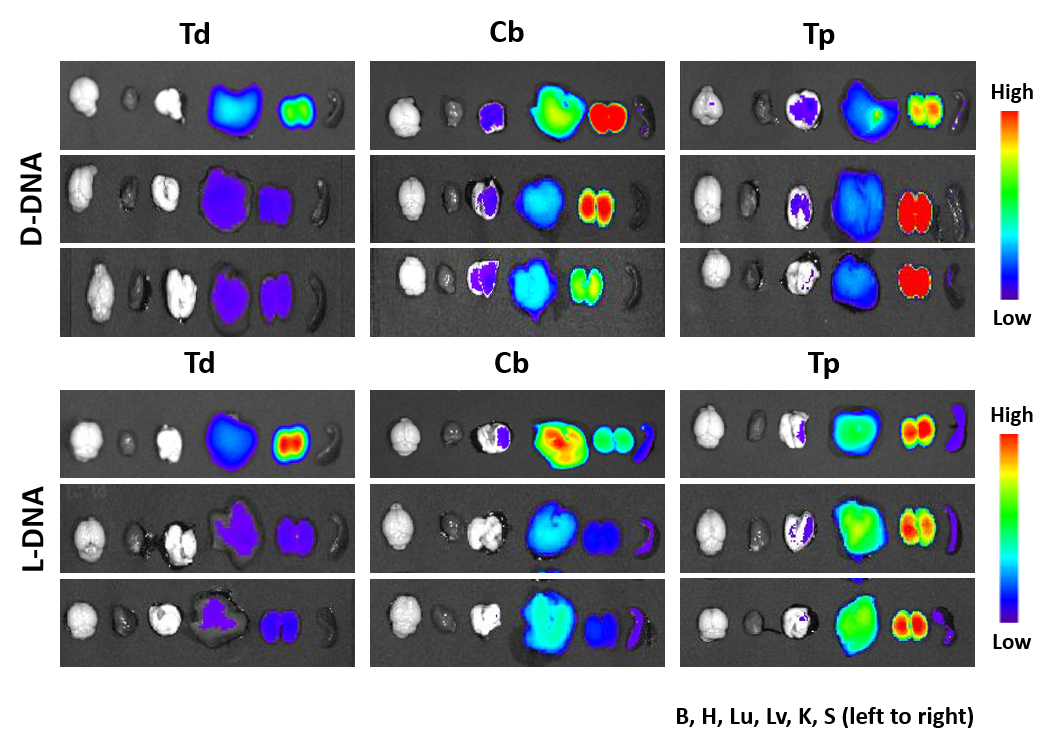


**Figure S3.** *Ex vivo* images of major organs showing the distribution of Cy5.5-labeled DNA nanostructures at 2 h post injection (2 µM, 200 µL) in BALB/c mice.

**Figure S4.** PAGE analysis of the intact form of S1 oligonucleotides labeled with (a) FAM and (b) Cy5.5 in brain lysates at 2 h post injection (n = 3). The amounts estimated by quantification of band intensity were shown in Fig 3b. M indicates size markers.

**Figure S5**. Representative ESI-MS spectra for detection of intact biotinylated S5 oligonucleotides in brain lysates (n = 2).

**Figure S6**. (a) Fluorescence images of FAM-labeled DNA nanostructures and a single-stranded D-DNA (SS, D-Cb-S1) after crossing the *in vitro* BBB model (n = 4) (b) TEER measurement before and after transcytosis (n = 4, mean ± SEM) (c) Apparent permeability coefficient (*P*_app_) of DNA nanostructures and SS measured using the *in vitro* BBB model (*n* = 4, mean ± SEM, *****P* < 0.0001 *vs*. D-Cb).


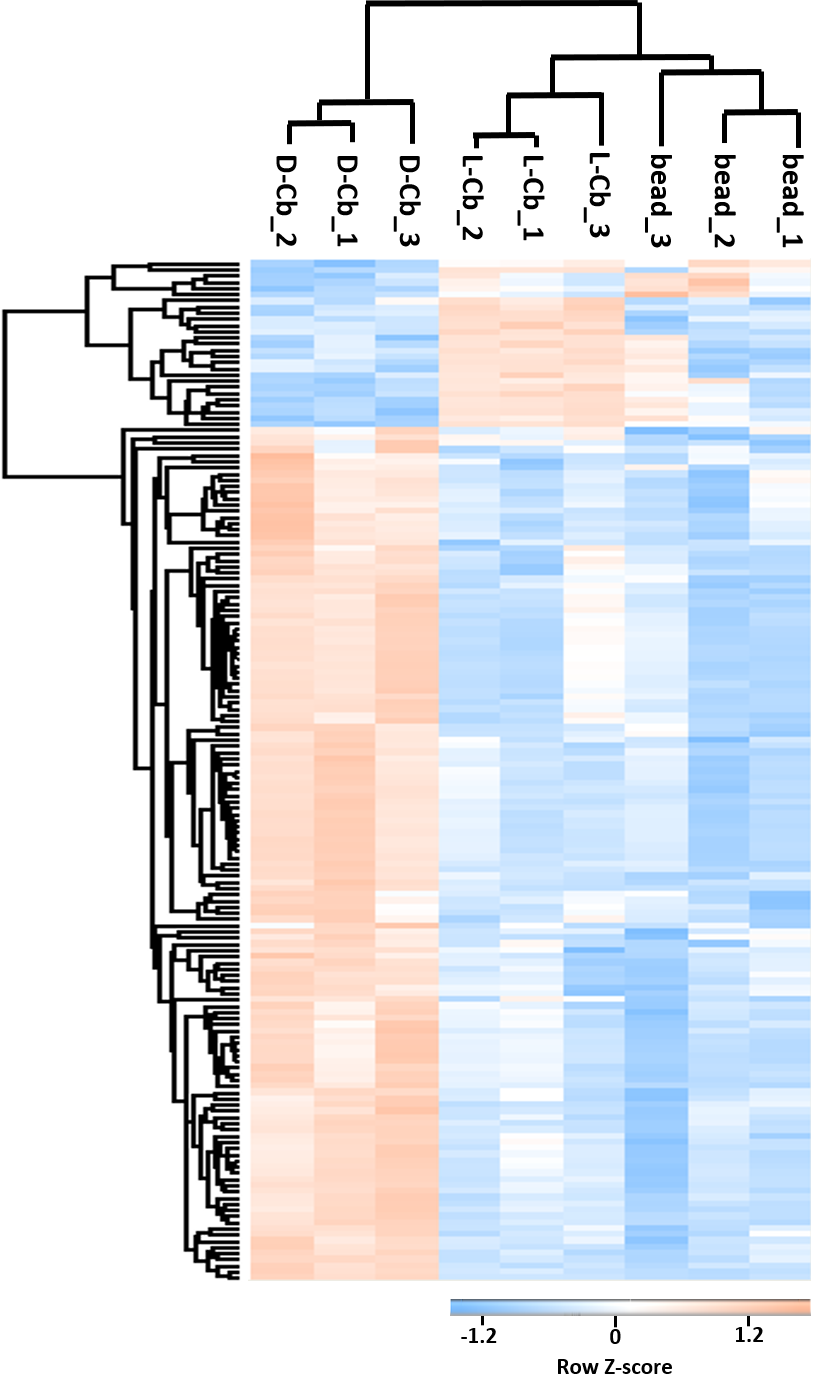


**Figure S7.** Heat map exhibiting hierarchical clustering of 165 proteins with statistically significant changes (*P*-value < 0.05) from three types of samples pulled down with bead, D-Cb, and L-Cb. The rows represent each protein and the columns show three technical replicates of samples pulled down with bead, D-Cb, and L-Cb. Hierarchical clustering of the 165 proteins was performed on log-transformed normalized abundance values after z-score normalization of the data in Perseus software (1.6.14.0).

**
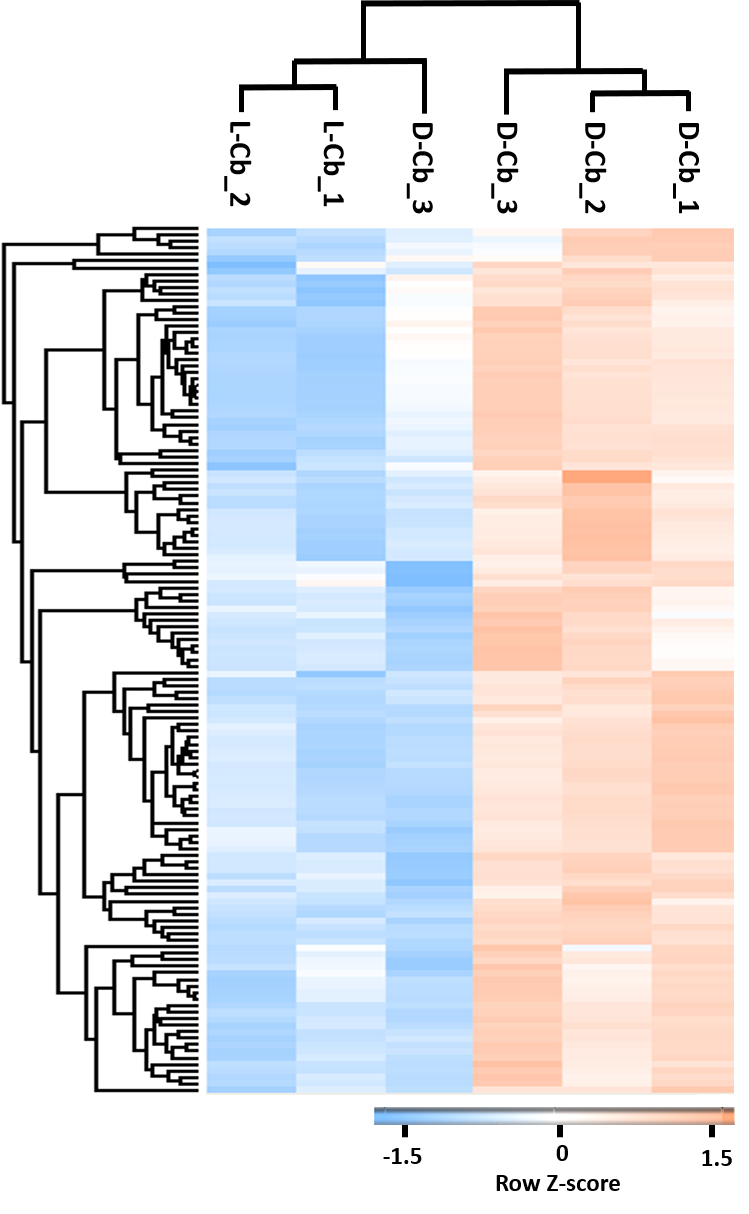
**

**Figure S8.** Heat map exhibiting hierarchical clustering of 133 proteins with statistically significant changes (*P*-value < 0.05) between two types of samples pulled down with D-Cb and L-Cb. The rows represent each protein and the columns show three LC-MS/MS runs of samples pulled down with bead, D-Cb and L-Cb. Hierarchical clustering of the 133 proteins was performed in Perseus software (1.6.14.0) on log-transformed normalized abundance values after z-score normalization of the data.

**Figure S9.** (a) Fluorescence images of FAM-labeled D-Cb (initial dose: 1 µM, 100 µL) after crossing a BBB monolayer in the presence or absence of antibodies (TfR Ab, LRP1 Ab, LRP2 Ab or LDLR Ab). (b) Flow cytometric analysis of bEnd.3 cells after treatment with L-Cb in 10% FBS-containing media in the presence or absence of anti-TfR, anti-LRP1, anti-LRP2 or anti-LDLR antibodies (n = 3, mean ± SEM, ns; no statistically significant *vs*. No Ab groups). Ab denotes antibody. (c) TEER before and after treatment with antibodies (n = 4, mean ± SEM).

**Figure S10.** PAGE analysis of the intact form of S1 oligonucleotides (a; Cy5.5-labeled D-Cb and b; Cy5.5-labeled L-Cb) in brain lysates (GBM mouse) at 2 h post injection (n=3). Standard curves of band intensity to quantify the intact form of the oligonucleotides. The estimated amounts were used in Fig 5c.

**
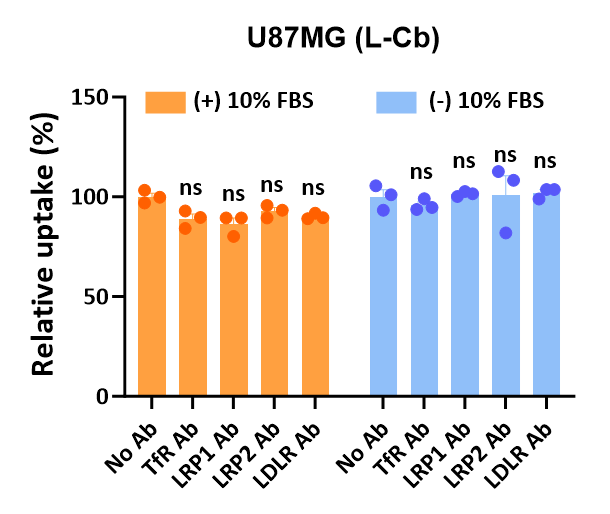
**

**Figure S11**. Flow cytometric analysis of U87MG cells after treatment with L-Cb in 10% FBS-containing media in the presence or absence of anti-TfR, anti-LRP1, anti-LRP2 or anti-LDLR antibodies (n = 3, mean ± SEM, ns; no statistically significant *vs*. No Ab groups. Ab denotes antibody.


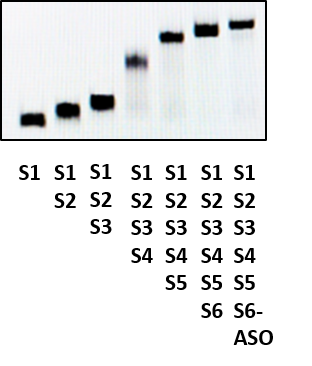


**Figure S12.** Native PAGE (6%) to verify step-wise self-assembly of ASO-loaded D-Cb.


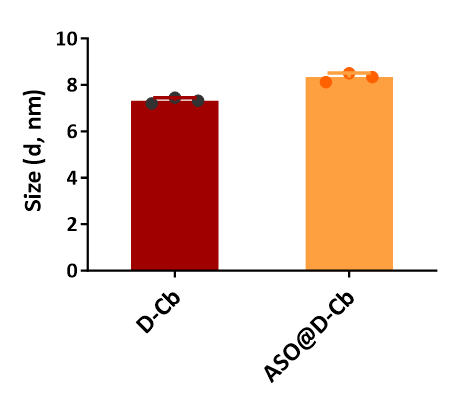


**Figure S13.** DLS of D-Cb and ASO@D-Cb showing their hydrodynamic sizes (mean ± SEM, n = 3)

**Figure S14**. Western blotting image of PLK1 levels in U87MG cells (n = 3). LF denotes lipofectamine RNAiMax (used as a positive control).


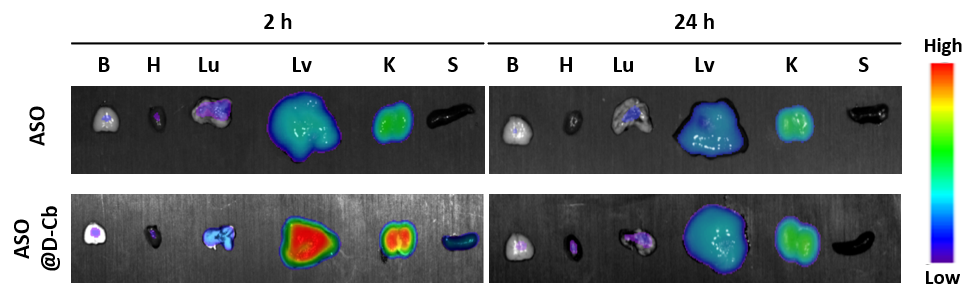


**Figure S15**. *Ex vivo* images of major organs excised from mice at 2 h and 24 h post administration of Cy5.5 labeled ASO and ASO@D-Cb in GBM mice. (brain: B, heart: H, lung: Lu, liver: Lv, kidney: K, spleen: S)

**Figure S16**. PAGE analysis of the intact form of (a) Cy5.5-labeled ASO or (b) ASO-linked S6 oligonucleotides in brain lysates at 2 h post injection (GBM mice, n = 3). Standard curves of band intensity to quantify the intact form of the oligonucleotides. The estimated amounts were shown in Fig 6g. M indicates size markers


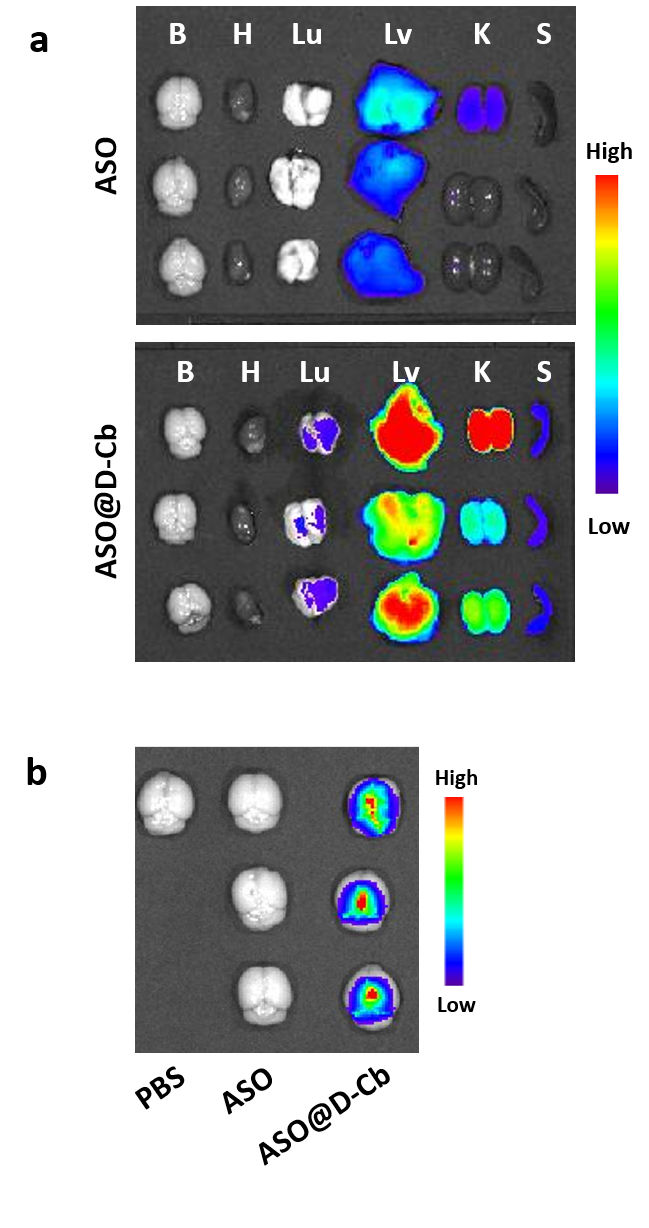


**Figure S17**. *Ex vivo* images of (a) major organs or (b) brain excised from mice at 2 h post administration of Cy5.5-labeled ASO and ASO@D-Cb in healthy mice (n = 3, brain: B, heart: H, lung: Lu, liver: Lv, kidney: K, spleen: S).

**Figure S18.** PAGE analysis of the intact form of (a) Cy5.5-labeled ASO or (b) ASO-linked S6 oligonucleotides in brain lysates at 2 h post injection (healthy mice, n = 3). Standard curves of band intensity to quantify the intact form of the oligonucleotides. The estimated amounts were shown in Fig 6g. M indicates size markers.


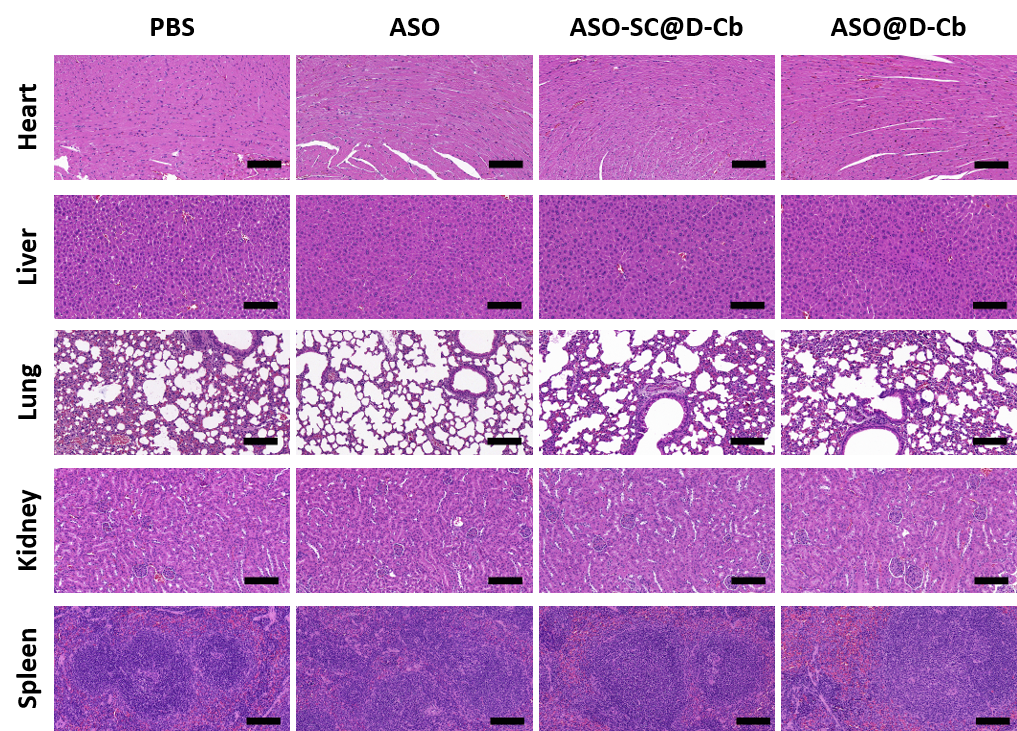


**Figure S19.** Systemic toxicity test for PBS, ASO, ASO-SC@D-Cb and ASO@D-Cb. Histological images of H&E-stained tissues (heart, liver, lung, kidney and spleen). Magnification: 200x, scale bar: 100 µm.

**Figure S20.** Western blot images of PLK1 protein in brains from GBM mice after treatment of PBS, ASO, ASO-SC@D-Cb or ASO@D-Cb (n = 4).


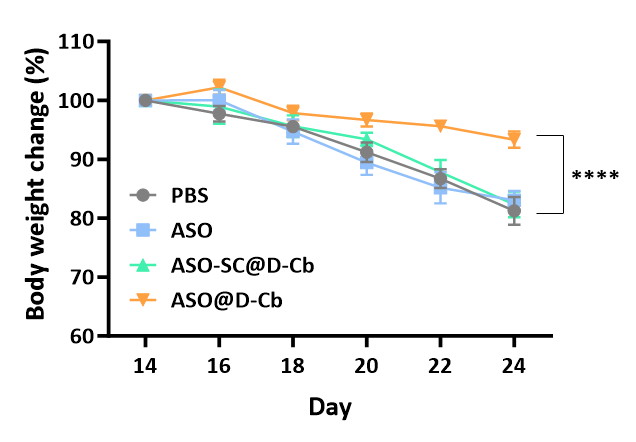


**Figure S21.** Mice body weights monitored every 2 days during ASO@D-Cb treatment. The data represent the mean ± SEM (n = 4); *****P* < 0.0001 vs. PBS, vs. ASO, *vs*. ASO-SC@D-Cb

**Table S1.** Oligonucleotide sequences used in this study.

|  | | **Sequence (5’ to 3’)** |
| --- | --- | --- |
| **Td** | S1 | GGG ATC CCG ATT CGA GAC AGC ATT TCT CCC ACA C |
|  | S2 | CGT GGT AGG TTT TGC TGT CTC GTT AGC GCC GGC C |
|  | S3 | TCG GGA TCC CTT CAC GGG CAA CTT GGC CGG CGC T |
|  | S4 | ACC TAC CAC GTT GTT GCC CGT GTT GTG TGG GAG A |
| **Tp** | S1 | TGC TGT CTC GTT CGT GGT AGG TTT GCA GAA GGT C |
|  | S2 | CGA GAC AGC ATT CAC GGG CAA CTT TCT CCC ACA CTT GGG AAA GGT C |
|  | S3 | GTG TGG GAG ATT CCG AGG GAT CTT GCG GAT TGT A |
|  | S4 | GGC CGG CGC TTT ACC TAC CAC GTT GAC CTT TCC CTT TAC AAT CCG C |
|  | S5 | AGC GCC GGC CTT GAT CCC TCG GTT GTT GCC CGT GTT GAC CTT CTG C |
| **Cb** | S1 | GTG TGG GAG ATT AGT CAT TAA GTT TAC AAT CCG CTT GTA ATC GTA G |
|  | S2 | GTT GCC CGT GTT CTA CGA TTA CTT GGT CGG GAA ATT CGT GGT AGG T |
|  | S3 | GCG GAT TGT ATT TAG GGG ACA TTT CGA GAC AGC ATT TTT CCC GAC C |
|  | S4 | GCA GAA GGT CTT CCG AGG GAT CTT ACC TAC CAC GTT TGC TGT CTC G |
|  | S5 | AGC GCC GGC CTT TCT CCC ACA CTT CAC GGG CAA CTT GAT CCC TCG G |
|  | S6 | CTT AAT GAC TTT GGC CGG CGC TTT GAC CTT CTG CTT ATG TCC CCT A |

**Table S2.** ESI-MS characterization of oligonucleotides used for assembly of structures.

| **D-DNA** | | | Estimated  **[M-H]^-^** | Observed | **L-DNA** | | | Estimated  **[M-H]^-^** | Observed |
| --- | --- | --- | --- | --- | --- | --- | --- | --- | --- |
| **Td** | S1 | 3’FAM | 10946.3 | 10949.3 | **Td** | S1 | 3’FAM | 10946.3 | 10945.9 |
|  | S2 | 3’NH_2_ | 10642.8 | 10643.5 |  | S2 | 3’NH_2_ | 10642.8 | 10643.4 |
|  | S3 | 3’NH_2_ | 10590.7 | 10590.0 |  | S3 | 3’NH_2_ | 10590.7 | 10590.7 |
|  | S4 | 3’NH_2_ | 10659.8 | 10660.0 |  | S4 | 3’NH_2_ | 10659.8 | 10660.0 |
| **Tp** | S1 | 3’FAM | 11125.4 | 11128.4 | **Tp** | S1 | 3’FAM | 11125.4 | 11128.4 |
|  | S2 | 3’NH_2_ | 14283.2 | 14283.6 |  | S2 | 3’NH_2_ | 14283.2 | 14283.8 |
|  | S3 | 3’NH_2_ | 10803.9 | 10804.5 |  | S3 | 3’NH_2_ | 10803.9 | 10804.5 |
|  | S4 | 3’NH_2_ | 14103.0 | 14103.8 |  | S4 | 3’NH_2_ | 14103.0 | 14103.4 |
|  | S5 | 3’NH_2_ | 14222.1 | 14222.3 |  | S5 | 3’NH_2_ | 14222.1 | 14222.3 |
| **Cb** | S1 | 3’FAM | 14847.9 | 14847.3 | **Cb** | S1 | 3’FAM | 14847.9 | 14830.6 |
|  | S2 | 3’NH_2_ | 14428.2 | 14428.3 |  | S2 | 3’NH_2_ | 14428.2 | 14428.4 |
|  | S3 | 3’NH_2_ | 14350.2 | 14350.4 |  | S3 | 3’NH_2_ | 14350.2 | 14350.0 |
|  | S4 | 3’NH_2_ | 14287.1 | 14287 |  | S4 | 3’NH_2_ | 14287.1 | 14287 |
|  | S5 | 3’NH_2_ | 14138.0 | 14137.9 |  | S5 | 3’NH_2_ | 14138.0 | 14137.8 |
|  | S6 | 3’NH_2_ | 14179.1 | 14179.4 |  | S6 | 3’NH_2_ | 14179.1 | 14179.3 |

**Table S3**. Identified proteins from three types samples from pulled down with bead, D-Cb, and L-Cb. ^a^PSM represents peptide spectrum match, which corresponds to tandem MS/MS spectrum matched to identification of a protein. ^b^AAs and cMW represent, respectively, amino acids and molecular weight. ^ǂ^'O' means 'identified' in the corresponding pulled down samples whereas 'X' means 'not identified' in the samples.

| **Accession No.** | **Protein description** | **Gene symbol** | **Coverage [%]** | **# PSMs^a^** | **# Unique Peptides** | **# AAs^b^** | **MW^c^ [kDa]** | **calc. pI** | **Bead** | **D-Cb** | **L-Cb** |
| --- | --- | --- | --- | --- | --- | --- | --- | --- | --- | --- | --- |
| P63101 | 14-3-3 protein zeta/delta | YWHAZ | 20 | 79 | 4 | 245 | 27.8 | 4.79 | O | O | O |
| P68134 | Actin, alpha skeletal muscle | ACTA1 | 55 | 306 | 7 | 377 | 42 | 5.39 | O | O | O |
| P60710 | Actin, cytoplasmic 1 | ACTB | 61 | 417 | 6 | 375 | 41.7 | 5.48 | O | O | O |
| Q60994 | Adiponectin | ADIPOQ | 26 | 69 | 6 | 247 | 26.8 | 5.57 | O | O | O |
| O89020 | Afamin | AFM | 45 | 216 | 24 | 608 | 69.3 | 5.78 | O | O | O |
| P07724 | Albumin | ALB | 90 | 13037 | 78 | 608 | 68.6 | 6.07 | O | O | O |
| Q60590 | Alpha-1-acid glycoprotein 1 | ORM1 | 23 | 58 | 5 | 207 | 23.9 | 5.85 | O | O | O |
| P22599 | Alpha-1-antitrypsin 1-2 | SERPINA1B | 56 | 1071 | 7 | 413 | 45.9 | 5.54 | O | O | O |
| Q00896 | Alpha-1-antitrypsin 1-3 | SERPINA1C | 52 | 1463 | 5 | 412 | 45.8 | 5.44 | O | O | O |
| Q00897 | Alpha-1-antitrypsin 1-4 | SERPINA1D | 49 | 835 | 4 | 413 | 46 | 5.44 | O | O | O |
| Q00898 | Alpha-1-antitrypsin 1-5 | SERPINA1E | 29 | 537 | 3 | 413 | 45.9 | 5.73 | O | O | O |
| Q61247 | Alpha-2-antiplasmin | SERPINF2 | 25 | 187 | 9 | 491 | 54.9 | 6.3 | O | O | O |
| P29699 | Alpha-2-HS-glycoprotein | AHSG | 46 | 575 | 9 | 345 | 37.3 | 6.51 | O | O | O |
| P00687 | Alpha-amylase 1 | AMY1 | 18 | 102 | 7 | 511 | 57.6 | 6.96 | O | O | O |
| P10107 | Annexin A1 | ANXA1 | 16 | 61 | 7 | 346 | 38.7 | 7.37 | O | O | O |
| P07356 | Annexin A2 | ANXA2 | 33 | 48 | 10 | 339 | 38.7 | 7.69 | O | O | O |
| P32261 | Antithrombin-III | SERPINC1 | 47 | 315 | 15 | 465 | 52 | 6.46 | O | O | O |
| Q00623 | Apolipoprotein A-I | APOA1 | 52 | 1024 | 16 | 264 | 30.6 | 5.73 | O | O | O |
| P06728 | Apolipoprotein A-IV | APOA4 | 63 | 351 | 19 | 395 | 45 | 5.47 | O | O | O |
| P08226 | Apolipoprotein E | APOE | 34 | 53 | 9 | 311 | 35.8 | 5.68 | O | O | O |
| Q01339 | Beta-2-glycoprotein 1 | APOH | 44 | 194 | 15 | 345 | 38.6 | 8.22 | O | O | O |
| P08607 | C4b-binding protein | C4BPA | 25 | 74 | 9 | 469 | 51.5 | 7.15 | O | O | O |
| P23953 | Carboxylesterase 1C | CES1C | 38 | 295 | 15 | 554 | 61 | 5.06 | O | O | O |
| Q9DBB9 | Carboxypeptidase N subunit 2 | CPN2 | 22 | 40 | 8 | 547 | 60.4 | 5.88 | O | O | O |
| Q9QWK4 | CD5 antigen-like | CD5L | 29 | 75 | 9 | 352 | 38.8 | 5.16 | O | O | O |
| Q61147 | Ceruloplasmin | CP | 53 | 1046 | 48 | 1061 | 121.1 | 5.85 | O | O | O |
| Q06890 | Clusterin | CLU | 16 | 148 | 7 | 448 | 51.6 | 5.67 | O | O | O |
| O88947 | Coagulation factor X | F10 | 22 | 25 | 6 | 481 | 54 | 5.66 | O | O | O |
| P14106 | Complement C1q subcomponent subunit B | C1QB | 27 | 87 | 5 | 253 | 26.7 | 8.15 | O | O | O |
| Q8CG16 | Complement C1r-A subcomponent | C1RA | 7 | 22 | 4 | 707 | 80 | 5.66 | O | O | O |
| P01027 | Complement C3 | C3 | 71 | 2134 | 91 | 1663 | 186.4 | 6.73 | O | O | O |
| P01029 | Complement C4-B | C4B | 39 | 264 | 45 | 1738 | 192.8 | 7.53 | O | O | O |
| P06684 | Complement C5 | C5 | 29 | 265 | 37 | 1680 | 188.8 | 6.81 | O | O | O |
| P04186 | Complement factor B | CFB | 31 | 197 | 23 | 761 | 85 | 7.37 | O | O | O |
| P06909 | Complement factor H | CFH | 44 | 388 | 41 | 1234 | 139 | 6.99 | O | O | O |
| Q61129 | Complement factor I | CFI | 23 | 56 | 9 | 603 | 67.2 | 7.46 | O | O | O |
| Q06770 | Corticosteroid-binding globulin | SERPINA6 | 16 | 44 | 6 | 397 | 44.7 | 5.24 | O | O | O |
| P10126 | Elongation factor 1-alpha 1 | EEF1A1 | 21 | 55 | 8 | 462 | 50.1 | 9.01 | O | O | O |
| P20029 | Endoplasmic reticulum chaperone BiP | HSPA5 | 9 | 33 | 3 | 655 | 72.4 | 5.16 | O | O | O |
| Q01279 | Epidermal growth factor receptor | EGFR | 11 | 56 | 10 | 1210 | 134.8 | 6.86 | O | O | O |
| Q9QXC1 | Fetuin-B | FETUB | 38 | 30 | 8 | 388 | 42.7 | 6.61 | O | O | O |
| P11276 | Fibronectin | FN1 | 26 | 324 | 44 | 2477 | 272.4 | 5.59 | O | O | O |
| P05064 | Fructose-bisphosphate aldolase A | ALDOA | 24 | 27 | 7 | 364 | 39.3 | 8.09 | O | O | O |
| P13020 | Gelsolin | GSN | 38 | 298 | 23 | 780 | 85.9 | 6.18 | O | O | O |
| P01898 | H-2 class I histocompatibility antigen, Q10 alpha chain | H2-Q10 | 46 | 141 | 11 | 325 | 37.2 | 5.25 | O | O | O |
| Q61646 | Haptoglobin | HP | 59 | 495 | 18 | 347 | 38.7 | 6.29 | O | O | O |
| P63017 | Heat shock cognate 71 kDa protein | HSPA8 | 10 | 32 | 4 | 646 | 70.8 | 5.52 | O | O | O |
| P01942 | Hemoglobin subunit alpha | HBA | 84 | 741 | 9 | 142 | 15.1 | 8.22 | O | O | O |
| P02088 | Hemoglobin subunit beta-1 | HBB-B1 | 88 | 743 | 6 | 147 | 15.8 | 7.65 | O | O | O |
| Q91X72 | Hemopexin | HPX | 66 | 1889 | 32 | 460 | 51.3 | 7.8 | O | O | O |
| P49182 | Heparin cofactor 2 | SERPIND1 | 23 | 60 | 8 | 478 | 54.5 | 7.34 | O | O | O |
| Q9ESB3 | Histidine-rich glycoprotein | HRG | 28 | 313 | 14 | 525 | 59.1 | 7.66 | O | O | O |
| Q64475 | Histone H2B type 1-B | H2BC3 | 37 | 13 | 4 | 126 | 13.9 | 10.32 | O | O | O |
| P62806 | Histone H4 | H4F16 | 55 | 94 | 8 | 103 | 11.4 | 11.36 | O | O | O |
| P01878 | Ig alpha chain C region |  | 51 | 354 | 12 | 344 | 36.9 | 5.06 | O | O | O |
| P01869 | Ig gamma-1 chain C region, membrane-bound form | IGHG1 | 44 | 330 | 12 | 393 | 43.4 | 6.44 | O | O | O |
| P01864 | Ig gamma-2A chain C region secreted form |  | 25 | 513 | 4 | 335 | 36.6 | 8.22 | O | O | O |
| P01865 | Ig gamma-2A chain C region, membrane-bound form | IGH-1A | 43 | 492 | 7 | 398 | 43.9 | 6.29 | O | O | O |
| P03987 | Ig gamma-3 chain C region |  | 60 | 153 | 13 | 398 | 43.9 | 7.14 | O | O | O |
| P18531 | Ig heavy chain V region 3-6 | IGHV3-6 | 31 | 33 | 2 | 116 | 13.1 | 8.78 | O | O | O |
| P18527 | Ig heavy chain V region 914 |  | 47 | 47 | 2 | 97 | 10.7 | 9.17 | O | O | O |
| P01746 | Ig heavy chain V region 93G7 |  | 17 | 25 | 2 | 140 | 15.5 | 8.4 | O | O | O |
| P06330 | Ig heavy chain V region AC38 205.12 |  | 72 | 146 | 3 | 118 | 12.9 | 7.11 | O | O | O |
| P01791 | Ig heavy chain V region HPCM6 |  | 20 | 22 | 2 | 123 | 13.9 | 6.06 | O | O | O |
| P18524 | Ig heavy chain V region RF |  | 44 | 38 | 3 | 117 | 12.9 | 9.29 | O | O | O |
| P01807 | Ig heavy chain V region X44 |  | 48 | 82 | 2 | 119 | 13.2 | 7.94 | O | O | O |
| P01632 | Ig kappa chain V-I region S107A | IGKV7-33 | 14 | 17 | 2 | 114 | 12.7 | 8.98 | O | O | O |
| P01631 | Ig kappa chain V-II region 26-10 |  | 43 | 41 | 4 | 113 | 12.3 | 8.88 | O | O | O |
| P01630 | Ig kappa chain V-II region 7S34.1 |  | 21 | 21 | 2 | 113 | 12.5 | 8.65 | O | O | O |
| P01638 | Ig kappa chain V-V region L6 (Fragment) |  | 36 | 81 | 3 | 115 | 13 | 7.81 | O | O | O |
| P01642 | Ig kappa chain V-V region L7 (Fragment) | GM10881 | 23 | 12 | 2 | 115 | 12.6 | 5.94 | O | O | O |
| P01636 | Ig kappa chain V-V region MOPC 149 |  | 35 | 18 | 3 | 108 | 12 | 7.28 | O | O | O |
| P01843 | Ig lambda-1 chain C region |  | 81 | 212 | 5 | 105 | 11.6 | 6.27 | O | O | O |
| P01723 | Ig lambda-1 chain V region |  | 34 | 32 | 2 | 117 | 12.2 | 5.21 | O | O | O |
| P01844 | Ig lambda-2 chain C region | IGLC2 | 86 | 76 | 5 | 104 | 11.2 | 6.27 | O | O | O |
| P01728 | Ig lambda-2 chain V region |  | 34 | 22 | 2 | 117 | 12.2 | 5.74 | O | O | O |
| P01867 | Immunoglobulin heavy constant gamma 2B | IGHG2B | 45 | 430 | 10 | 404 | 44.2 | 6.52 | O | O | O |
| P01872 | Immunoglobulin heavy constant mu | IGHM | 45 | 454 | 18 | 454 | 49.9 | 7.01 | O | O | O |
| P01635 | Immunoglobulin kappa chain variable 12-41 (Fragment) | IGKV12-41 | 41 | 60 | 4 | 115 | 12.6 | 8.31 | O | O | O |
| P01633 | Immunoglobulin kappa chain variable 6-17 | IGKV6-17 | 17 | 35 | 3 | 149 | 16.4 | 6.92 | O | O | O |
| P01837 | Immunoglobulin kappa constant | IGKC | 93 | 797 | 11 | 107 | 11.9 | 5.9 | O | O | O |
| Q9DBD0 | Inhibitor of carbonic anhydrase | ICA | 56 | 220 | 27 | 700 | 76.7 | 7.25 | O | O | O |
| A6X935 | Inter alpha-trypsin inhibitor, heavy chain 4 | ITIH4 | 46 | 381 | 33 | 942 | 104.6 | 6.4 | O | O | O |
| Q61702 | Inter-alpha-trypsin inhibitor heavy chain H1 | ITIH1 | 36 | 136 | 22 | 907 | 101 | 6.96 | O | O | O |
| Q61703 | Inter-alpha-trypsin inhibitor heavy chain H2 | ITIH2 | 29 | 124 | 20 | 946 | 105.9 | 7.27 | O | O | O |
| Q61704 | Inter-alpha-trypsin inhibitor heavy chain H3 | ITIH3 | 26 | 104 | 17 | 889 | 99.3 | 6.05 | O | O | O |
| O08677 | Kininogen-1 | KNG1 | 25 | 324 | 13 | 661 | 73.1 | 6.54 | O | O | O |
| P28665 | Murinoglobulin-1 | MUG1 | 55 | 1350 | 39 | 1476 | 165.2 | 6.42 | O | O | O |
| P28666 | Murinoglobulin-2 | MUG2 | 47 | 802 | 29 | 1451 | 162.3 | 6.74 | O | O | O |
| O70362 | Phosphatidylinositol-glycan-specific phospholipase D | GPLD1 | 18 | 53 | 13 | 837 | 93.2 | 7.12 | O | O | O |
| P09411 | Phosphoglycerate kinase 1 | PGK1 | 10 | 16 | 3 | 417 | 44.5 | 7.9 | O | O | O |
| Q9DBJ1 | Phosphoglycerate mutase 1 | PGAM1 | 14 | 16 | 3 | 254 | 28.8 | 7.18 | O | O | O |
| P26262 | Plasma kallikrein | KLKB1 | 26 | 93 | 13 | 638 | 71.3 | 8.02 | O | O | O |
| P97290 | Plasma protease C1 inhibitor | SERPING1 | 41 | 129 | 16 | 504 | 55.5 | 6.29 | O | O | O |
| P20918 | Plasminogen | PLG | 72 | 543 | 47 | 812 | 90.7 | 6.6 | O | O | O |
| Q61838 | Pregnancy zone protein | PZP | 61 | 2568 | 71 | 1495 | 165.7 | 6.68 | O | O | O |
| P11680 | Properdin | CFP | 14 | 68 | 4 | 464 | 50.3 | 7.84 | O | O | O |
| Q07456 | Protein AMBP | AMBP | 19 | 57 | 6 | 349 | 39 | 6.32 | O | O | O |
| Q9D2Q8 | Protein S100-A14 | S100A14 | 21 | 12 | 2 | 104 | 11.6 | 5.5 | O | O | O |
| P19221 | Prothrombin | F2 | 31 | 170 | 16 | 618 | 70.2 | 6.43 | O | O | O |
| Q00724 | Retinol-binding protein 4 | RBP4 | 34 | 23 | 5 | 201 | 23.2 | 5.99 | O | O | O |
| P07759 | Serine protease inhibitor A3K | SERPINA3K | 61 | 1041 | 16 | 418 | 46.9 | 5.16 | O | O | O |
| Q921I1 | Serotransferrin | TF | 73 | 5294 | 65 | 697 | 76.7 | 7.18 | O | O | O |
| P12246 | Serum amyloid P-component | APCS | 27 | 104 | 6 | 224 | 26.2 | 6.35 | O | O | O |
| P52430 | Serum paraoxonase/arylesterase 1 | PON1 | 34 | 109 | 8 | 355 | 39.5 | 5.22 | O | O | O |
| Q8BND5 | Sulfhydryl oxidase 1 | QSOX1 | 18 | 60 | 11 | 748 | 82.7 | 7.17 | O | O | O |
| P35441 | Thrombospondin-1 | THBS1 | 26 | 176 | 23 | 1170 | 129.6 | 4.96 | O | O | O |
| P07309 | Transthyretin | TTR | 67 | 449 | 8 | 147 | 15.8 | 6.16 | O | O | O |
| P17751 | Triosephosphate isomerase | TPI1 | 30 | 17 | 6 | 249 | 26.7 | 7.3 | O | O | O |
| P68368 | Tubulin alpha-4A chain | TUBA4A | 11 | 18 | 4 | 448 | 49.9 | 5.06 | O | O | O |
| P20152 | Vimentin | VIM | 26 | 190 | 9 | 466 | 53.7 | 5.12 | O | O | O |
| P21614 | Vitamin D-binding protein | GC | 74 | 474 | 26 | 476 | 53.6 | 5.5 | O | O | O |
| P29788 | Vitronectin | VTN | 20 | 98 | 5 | 478 | 54.8 | 5.88 | O | O | O |
| Q64726 | Zinc-alpha-2-glycoprotein | AZGP1 | 35 | 69 | 8 | 307 | 35.3 | 6.18 | O | O | O |
| P07361 | Alpha-1-acid glycoprotein 2 | ORM2 | 15 | 33 | 2 | 207 | 23.8 | 5.45 | X | O | O |
| P11859 | Angiotensinogen | AGT | 22 | 28 | 8 | 477 | 52 | 5.44 | X | O | O |
| E9Q414 | Apolipoprotein B-100 | APOB | 9 | 103 | 32 | 4505 | 509.1 | 6.81 | X | O | O |
| P01887 | Beta-2-microglobulin | B2M | 16 | 15 | 2 | 119 | 13.8 | 8.44 | X | O | O |
| Q9JJN5 | Carboxypeptidase N catalytic chain | CPN1 | 22 | 37 | 7 | 457 | 51.8 | 8.28 | X | O | O |
| O88783 | Coagulation factor V | F5 | 2 | 26 | 4 | 2183 | 247.1 | 6.05 | X | O | O |
| Q07968 | Coagulation factor XIII B chain | F13B | 4 | 15 | 3 | 669 | 76.1 | 6.92 | X | O | O |
| P06683 | Complement component C9 | C9 | 8 | 14 | 3 | 548 | 62 | 5.78 | X | O | O |
| P01749 | Ig heavy chain V region 3 | IGHV1-61 | 28 | 66 | 2 | 117 | 13 | 7.87 | X | O | O |
| P01801 | Ig heavy chain V-III region J606 |  | 43 | 196 | 3 | 115 | 12.8 | 7.12 | X | O | O |
| P84750 | Ig kappa chain V region Mem5 (Fragment) |  | 16 | 3 | 2 | 121 | 13.2 | 8.81 | X | O | O |
| P01660 | Ig kappa chain V-III region PC 3741/TEPC 111 |  | 49 | 14 | 2 | 111 | 12.1 | 5.38 | X | O | O |
| P08071 | Lactotransferrin | LTF | 3 | 11 | 2 | 707 | 77.8 | 8.53 | X | O | O |
| P42703 | Leukemia inhibitory factor receptor | LIFR | 8 | 16 | 6 | 1092 | 122.5 | 6.04 | X | O | O |
| P26041 | Moesin | MSN | 5 | 4 | 2 | 577 | 67.7 | 6.6 | X | O | O |
| Q8VCS0 | N-acetylmuramoyl-L-alanine amidase | PGLYRP2 | 6 | 19 | 3 | 530 | 57.7 | 6.98 | X | O | O |
| P97298 | Pigment epithelium-derived factor | SERPINF1 | 27 | 17 | 8 | 417 | 46.2 | 6.98 | X | O | O |
| Q61233 | Plastin-2 | LCP1 | 3 | 16 | 2 | 627 | 70.1 | 5.33 | X | O | O |
| P31532 | Serum amyloid A-4 protein | SAA4 | 42 | 23 | 6 | 130 | 15.1 | 9.26 | X | O | O |
| Q62351 | Transferrin receptor protein 1 | TFRC | 13 | 19 | 10 | 763 | 85.7 | 6.57 | X | O | O |
| O70456 | 14-3-3 protein sigma | SFN | 17 | 19 | 4 | 248 | 27.7 | 4.78 | O | X | O |
| P17182 | Alpha-enolase | ENO1 | 6 | 3 | 2 | 434 | 47.1 | 6.8 | O | X | O |
| E9Q557 | Desmoplakin | DSP | 8 | 78 | 18 | 2883 | 332.7 | 6.8 | O | X | O |
| Q8VCM7 | Fibrinogen gamma chain | FGG | 7 | 12 | 2 | 436 | 49.4 | 5.86 | O | X | O |
| P16858 | Glyceraldehyde-3-phosphate dehydrogenase | GAPDH | 29 | 20 | 6 | 333 | 35.8 | 8.25 | O | X | O |
| Q61696 | Heat shock 70 kDa protein 1A | HSPA1A | 9 | 21 | 4 | 641 | 70 | 5.72 | O | X | O |
| P07901 | Heat shock protein HSP 90-alpha | HSP90AA1 | 4 | 9 | 2 | 733 | 84.7 | 5.01 | O | X | O |
| P11499 | Heat shock protein HSP 90-beta | HSP90AB1 | 8 | 14 | 4 | 724 | 83.2 | 5.03 | O | X | O |
| Q02257 | Junction plakoglobin | JUP | 10 | 21 | 6 | 745 | 81.7 | 6.14 | O | X | O |
| P11247 | Myeloperoxidase | MPO | 5 | 12 | 3 | 718 | 81.1 | 9.55 | O | X | O |
| Q8VDD5 | Myosin-9 | MYH9 | 2 | 6 | 3 | 1960 | 226.2 | 5.66 | O | X | O |
| P17742 | Peptidyl-prolyl cis-trans isomerase A | PPIA | 32 | 15 | 4 | 164 | 18 | 7.9 | O | X | O |
| P97350 | Plakophilin-1 | PKP1 | 6 | 24 | 4 | 728 | 80.8 | 8.91 | O | X | O |
| P48678 | Prelamin-A/C | LMNA | 6 | 14 | 3 | 665 | 74.2 | 6.98 | O | X | O |
| P09103 | Protein disulfide-isomerase | P4HB | 7 | 15 | 3 | 509 | 57 | 4.88 | O | X | O |
| P68372 | Tubulin beta-4B chain | TUBB4B | 21 | 43 | 2 | 445 | 49.8 | 4.89 | O | X | O |
| Q60930 | Voltage-dependent anion-selective channel protein 2 | VDAC2 | 11 | 3 | 2 | 295 | 31.7 | 7.49 | O | X | O |
| Q9R269 | Periplakin | PPL | 1 | 2 | 2 | 1755 | 203.9 | 5.54 | X | X | O |
| P98086 | Complement C1q subcomponent subunit A | C1QA | 16 | 29 | 3 | 245 | 26 | 9.11 | O | O | X |
| Q02105 | Complement C1q subcomponent subunit C | C1QC | 16 | 40 | 3 | 246 | 26 | 8.54 | O | O | X |
| Q8K182 | Complement component C8 alpha chain | C8A | 7 | 9 | 3 | 587 | 66 | 6.54 | O | O | X |
| Q8BH35 | Complement component C8 beta chain | C8B | 16 | 23 | 6 | 589 | 66.2 | 7.77 | O | O | X |
| P62897 | Cytochrome c, somatic | CYCS | 24 | 9 | 2 | 105 | 11.6 | 9.58 | O | O | X |
| Q08879 | Fibulin-1 | FBLN1 | 5 | 7 | 2 | 705 | 78 | 5.16 | O | O | X |
| Q9R098 | Hepatocyte growth factor activator | HGFAC | 6 | 53 | 3 | 653 | 70.5 | 7.03 | O | O | X |
| P06151 | L-lactate dehydrogenase A chain | LDHA | 16 | 38 | 4 | 332 | 36.5 | 7.74 | O | O | X |
| O70570 | Polymeric immunoglobulin receptor | PIGR | 5 | 12 | 2 | 771 | 84.9 | 5.4 | O | O | X |
| Q8R121 | Protein Z-dependent protease inhibitor | SERPINA10 | 17 | 27 | 6 | 448 | 51.8 | 5.67 | O | O | X |
| Q91WP6 | Serine protease inhibitor A3N | SERPINA3N | 20 | 251 | 5 | 418 | 46.7 | 5.82 | O | O | X |
| P61939 | Thyroxine-binding globulin | SERPINA7 | 11 | 28 | 4 | 418 | 47 | 6.54 | O | O | X |
| Q9QZ25 | Vascular non-inflammatory molecule 3 | VNN3 | 7 | 6 | 3 | 500 | 56.3 | 6.29 | O | O | X |
| Q6GQT1 | Alpha-2-macroglobulin-P | A2M | 3 | 36 | 2 | 1474 | 164.2 | 6.61 | X | O | X |
| Q9Z1R3 | Apolipoprotein M | APOM | 7 | 5 | 2 | 190 | 21.3 | 6.52 | X | O | X |
| P00920 | Carbonic anhydrase 2 | CA2 | 18 | 16 | 4 | 260 | 29 | 7.01 | X | O | X |
| Q9JHH6 | Carboxypeptidase B2 | CPB2 | 13 | 20 | 4 | 422 | 48.8 | 7.97 | X | O | X |
| P10605 | Cathepsin B | CTSB | 9 | 3 | 2 | 339 | 37.3 | 5.91 | X | O | X |
| Q03311 | Cholinesterase | BCHE | 4 | 2 | 2 | 603 | 68.4 | 7.25 | X | O | X |
| Q80YC5 | Coagulation factor XII | F12 | 16 | 29 | 6 | 597 | 65.7 | 6.84 | X | O | X |
| Q8CG14 | Complement C1s-1 subcomponent | C1S1 | 14 | 20 | 7 | 688 | 76.8 | 5.08 | X | O | X |
| P21180 | Complement C2 | C2 | 8 | 25 | 5 | 760 | 84.7 | 7.56 | X | O | X |
| Q8VCG4 | Complement component C8 gamma chain | C8G | 34 | 26 | 5 | 202 | 22.5 | 9.25 | X | O | X |
| P14847 | C-reactive protein | CRP | 17 | 28 | 3 | 225 | 25.3 | 6.2 | X | O | X |
| Q8BPB5 | EGF-containing fibulin-like extracellular matrix protein 1 | EFEMP1 | 6 | 6 | 2 | 493 | 54.9 | 5.14 | X | O | X |
| Q8K1B8 | Fermitin family homolog 3 | FERMT3 | 6 | 12 | 3 | 665 | 75.6 | 7.05 | X | O | X |
| Q8BTM8 | Filamin-A | FLNA | 1 | 11 | 2 | 2647 | 281 | 6.04 | X | O | X |
| Q923D2 | Flavin reductase (NADPH) | BLVRB | 13 | 5 | 2 | 206 | 22.2 | 7.01 | X | O | X |
| Q91Y97 | Fructose-bisphosphate aldolase B | ALDOB | 9 | 14 | 2 | 364 | 39.5 | 8.27 | X | O | X |
| P46412 | Glutathione peroxidase 3 | GPX3 | 29 | 21 | 5 | 226 | 25.4 | 8.22 | X | O | X |
| P14426 | H-2 class I histocompatibility antigen, D-K alpha chain | H2-D1 | 11 | 10 | 3 | 362 | 40.6 | 5.47 | X | O | X |
| P02089 | Hemoglobin subunit beta-2 | HBB-B2 | 62 | 343 | 3 | 147 | 15.9 | 8.05 | X | O | X |
| P06336 | Ig epsilon chain C region |  | 15 | 49 | 5 | 421 | 47.3 | 7.44 | X | O | X |
| P01670 | Ig kappa chain V-III region PC 6684 |  | 60 | 44 | 2 | 111 | 12 | 8 | X | O | X |
| P70389 | Insulin-like growth factor-binding protein complex acid labile subunit | IGFALS | 22 | 36 | 11 | 603 | 66.9 | 6.6 | X | O | X |
| P09581 | Macrophage colony-stimulating factor 1 receptor | CSF1R | 2 | 8 | 2 | 977 | 109.1 | 6.21 | X | O | X |
| P16301 | Phosphatidylcholine-sterol acyltransferase | LCAT | 5 | 2 | 2 | 438 | 49.7 | 6.43 | X | O | X |
| Q9Z126 | Platelet factor 4 | PF4 | 29 | 15 | 3 | 105 | 11.2 | 9.29 | X | O | X |
| O08742 | Platelet glycoprotein V | GP5 | 8 | 16 | 3 | 567 | 63.4 | 8.97 | X | O | X |
| Q60963 | Platelet-activating factor acetylhydrolase | PLA2G7 | 6 | 23 | 2 | 440 | 49.2 | 7.12 | X | O | X |
| P70274 | Selenoprotein P | SELENOP | 12 | 21 | 3 | 380 | 42.7 | 7.09 | X | O | X |
| Q03734 | Serine protease inhibitor A3M | SERPINA3M | 36 | 423 | 5 | 418 | 47 | 6.1 | X | O | X |
| P26039 | Talin-1 | TLN1 | 2 | 5 | 4 | 2541 | 269.7 | 6.18 | X | O | X |
| O88968 | Transcobalamin-2 | TCN2 | 16 | 3 | 3 | 430 | 47.6 | 6.33 | X | O | X |
| P82198 | Transforming growth factor-beta-induced protein ig-h3 | TGFBI | 3 | 2 | 2 | 683 | 74.6 | 7.06 | X | O | X |
| Q7TPR4 | Alpha-actinin-1 | ACTN1 | 6 | 16 | 2 | 892 | 103 | 5.38 | O | X | X |
| Q9JI91 | Alpha-actinin-2 | ACTN2 | 14 | 13 | 9 | 894 | 103.8 | 5.45 | O | X | X |
| Q9WUA3 | ATP-dependent 6-phosphofructokinase, platelet type | PFKP | 2 | 4 | 2 | 784 | 85.4 | 7.11 | O | X | X |
| P21550 | Beta-enolase | ENO3 | 5 | 3 | 2 | 434 | 47 | 7.18 | O | X | X |
| P07310 | Creatine kinase M-type | CKM | 11 | 3 | 2 | 381 | 43 | 7.06 | O | X | X |
| Q9WUB3 | Glycogen phosphorylase, muscle form | PYGM | 9 | 10 | 6 | 842 | 97.2 | 7.11 | O | X | X |
| P97457 | Myosin regulatory light chain 11 | MYL11 | 17 | 2 | 2 | 169 | 18.9 | 4.92 | O | X | X |
| Q5SX40 | Myosin-1 | MYH1 | 22 | 111 | 2 | 1942 | 223.2 | 5.76 | O | X | X |
| P13541 | Myosin-3 | MYH3 | 12 | 61 | 2 | 1940 | 223.7 | 5.81 | O | X | X |
| Q91Z83 | Myosin-7 | MYH7 | 10 | 29 | 2 | 1935 | 222.7 | 5.76 | O | X | X |
| P13542 | Myosin-8 | MYH8 | 20 | 97 | 2 | 1937 | 222.6 | 5.83 | O | X | X |
| A2ASS6 | Titin | TTN | 0 | 10 | 6 | 35213 | 3904.1 | 6.2 | O | X | X |
| P21107 | Tropomyosin alpha-3 chain | TPM3 | 26 | 53 | 2 | 285 | 33 | 4.72 | O | X | X |

**Table S4**. Proteins showing statistically significant changes (P-value <0.05) from label-free quantitative analyses of three types of samples pulled down with bead, D-Cb, and L-Cb by one-way analysis of variance (ANOVA). ^a^'-' represents that the abundance ratios cannot be calculated because of absence of the peptide peaks.

| **Accession No.** | **Protein description** | **Gene symbol** | **Normalized abundance**  **_bead** | | | **Normalized abundance**  **_ D-Cb** | | | **Normalized abundance**  **_L-Cb** | | | **P-value (one-way ANOVA)** | **Abundance ratio** | | |
| --- | --- | --- | --- | --- | --- | --- | --- | --- | --- | --- | --- | --- | --- | --- | --- |
|  |  |  | **1** | **2** | **3** | **1** | **2** | **3** | **1** | **2** | **3** |  | **D-Cb/L-Cb^a^** | **D-Cb/Bead^a^** | **L-Cb/Bead^a^** |
| P19221 | Prothrombin | F2 | 2.64E+07 | 2.18E+07 | 2.29E+07 | 1.27E+08 | 1.63E+08 | 1.52E+08 | 2.80E+07 | 2.81E+07 | 2.76E+07 | 6.59E-07 | 5.29 | 6.23 | 1.18 |
| E9Q557 | Desmoplakin | DSP | 6.10E+06 | 7.98E+06 | 1.14E+07 | 8.18E+04 | 2.48E+05 | 2.01E+05 | 1.18E+08 | 1.30E+08 | 1.95E+08 | 3.11E-06 | 0.00 | 0.02 | 17.37 |
| P06684 | Complement C5 | C5 | 1.07E+07 | 8.69E+06 | 9.72E+06 | 1.26E+08 | 2.34E+08 | 1.63E+08 | 1.81E+07 | 1.50E+07 | 1.18E+07 | 9.60E-06 | 11.65 | 17.97 | 1.54 |
| P52430 | Serum paraoxonase/arylesterase 1 | PON1 | 1.35E+07 | 1.60E+07 | 1.49E+07 | 1.85E+08 | 1.12E+08 | 1.87E+08 | 2.57E+07 | 1.80E+07 | 2.24E+07 | 1.59E-05 | 7.33 | 10.93 | 1.49 |
| Q9R269 | Periplakin | PPL | 1.82E+05 | Not detected | Not detected | Not detected | Not detected | Not detected | 8.20E+06 | 1.59E+07 | 2.12E+07 | 2.71E-05 | – | – | 82.95 |
| Q9R098 | Hepatocyte growth factor activator | HGFAC | 8.74E+05 | 1.03E+06 | 6.60E+05 | 5.67E+06 | 9.34E+06 | 7.83E+06 | 7.40E+05 | 1.00E+06 | 1.01E+06 | 2.87E-05 | 8.31 | 8.91 | 1.07 |
| Q61646 | Haptoglobin | HP | 1.02E+09 | 1.13E+09 | 9.61E+08 | 4.33E+09 | 3.45E+09 | 5.26E+09 | 1.24E+09 | 1.03E+09 | 1.30E+09 | 4.15E-05 | 3.66 | 4.20 | 1.15 |
| P98086 | Complement C1q subcomponent subunit A | C1QA | 5.50E+06 | 4.95E+06 | 5.74E+06 | 8.33E+07 | 5.19E+07 | 4.37E+07 | 5.51E+06 | 9.13E+06 | 5.25E+06 | 5.92E-05 | 8.99 | 11.05 | 1.23 |
| Q61247 | Alpha-2-antiplasmin | SERPINF2 | 4.97E+07 | 3.09E+07 | 2.60E+07 | 1.59E+08 | 1.60E+08 | 1.49E+08 | 3.99E+07 | 3.31E+07 | 3.46E+07 | 1.42E-04 | 4.35 | 4.39 | 1.01 |
| Q9Z1R3 | Apolipoprotein M | APOM | 1.66E+06 | 1.87E+06 | 3.15E+06 | 2.20E+07 | 1.55E+07 | 1.23E+07 | 2.67E+06 | 2.44E+06 | 2.69E+06 | 1.45E-04 | 6.38 | 7.44 | 1.17 |
| P26041 | Moesin | MSN | Not detected | Not detected | Not detected | 3.24E+05 | 5.27E+04 | 3.85E+05 | 1.23E+07 | 1.58E+07 | 2.14E+07 | 1.97E-04 | 0.02 | – | – |
| P35441 | Thrombospondin-1 | THBS1 | 1.06E+07 | 1.26E+07 | 1.15E+07 | 1.15E+08 | 2.05E+08 | 3.40E+08 | 2.59E+07 | 2.87E+07 | 1.51E+07 | 2.11E-04 | 9.45 | 18.95 | 2.00 |
| Q02105 | Complement C1q subcomponent subunit C | C1QC | 5.77E+06 | 4.10E+06 | 9.06E+06 | 8.11E+07 | 6.05E+07 | 4.38E+07 | 7.88E+06 | 1.11E+07 | 7.07E+06 | 2.46E-04 | 7.11 | 9.79 | 1.38 |
| O88783 | Coagulation factor V | F5 | 5.66E+05 | 5.09E+05 | 4.01E+05 | 1.03E+07 | 4.73E+06 | 1.60E+07 | 1.32E+06 | 8.04E+05 | 8.70E+05 | 2.58E-04 | 10.34 | 20.96 | 2.03 |
| P14106 | Complement C1q subcomponent subunit B | C1QB | 1.66E+07 | 1.84E+07 | 1.23E+07 | 2.18E+08 | 9.06E+07 | 1.14E+08 | 1.84E+07 | 2.33E+07 | 1.64E+07 | 2.61E-04 | 7.28 | 8.94 | 1.23 |
| P01029 | Complement C4-B | C4B | 4.70E+07 | 7.13E+07 | 2.93E+07 | 5.97E+08 | 4.88E+08 | 9.63E+08 | 1.26E+08 | 8.03E+07 | 7.66E+07 | 2.73E-04 | 7.25 | 13.88 | 1.91 |
| P01872 | Immunoglobulin heavy constant mu | IGHM | 2.14E+08 | 2.50E+08 | 1.55E+08 | 2.40E+09 | 1.49E+09 | 3.65E+09 | 4.06E+08 | 2.26E+08 | 3.83E+08 | 3.03E-04 | 7.42 | 12.17 | 1.64 |
| P01878 | Ig alpha chain C region |  | 3.87E+08 | 3.01E+08 | 2.11E+08 | 1.25E+09 | 1.74E+09 | 1.75E+09 | 3.93E+08 | 2.77E+08 | 2.28E+08 | 3.19E-04 | 5.29 | 5.28 | 1.00 |
| P21180 | Complement C2 | C2 | 7.54E+05 | 8.99E+05 | 2.92E+05 | 1.13E+07 | 1.01E+07 | 1.44E+07 | 1.87E+06 | 8.76E+05 | 1.24E+06 | 3.29E-04 | 8.98 | 18.40 | 2.05 |
| Q61233 | Plastin-2 | LCP1 | 5.26E+05 | 3.28E+05 | 1.96E+05 | 6.14E+05 | 5.73E+05 | 4.66E+05 | 6.48E+06 | 3.47E+06 | 3.27E+06 | 3.30E-04 | 0.13 | 1.58 | 12.59 |
| P01898 | H-2 class I histocompatibility antigen, Q10 alpha chain | H2-Q10 | 3.56E+07 | 5.22E+07 | 1.79E+07 | 4.02E+08 | 2.53E+08 | 4.63E+08 | 5.16E+07 | 4.34E+07 | 3.59E+07 | 4.34E-04 | 8.54 | 10.58 | 1.24 |
| P14847 | C-reactive protein | CRP | 6.92E+05 | 5.00E+05 | 1.10E+06 | 2.15E+07 | 1.10E+07 | 9.75E+06 | 1.19E+06 | 2.44E+06 | 9.75E+05 | 4.41E-04 | 9.17 | 18.43 | 2.01 |
| P14426 | H-2 class I histocompatibility antigen, D-K alpha chain | H2-D1 | 7.21E+05 | 9.16E+05 | 3.23E+05 | 8.76E+06 | 6.23E+06 | 1.08E+07 | 1.18E+06 | 7.56E+05 | 1.44E+06 | 4.65E-04 | 7.65 | 13.16 | 1.72 |
| Q61696 | Heat shock 70 kDa protein 1A | HSPA1A | 3.00E+06 | 3.75E+06 | 5.92E+06 | 4.72E+06 | 2.19E+06 | 5.72E+06 | 3.36E+07 | 6.05E+07 | 8.20E+07 | 4.76E-04 | 0.07 | 1.00 | 13.90 |
| P08226 | Apolipoprotein E | APOE | 1.85E+07 | 2.11E+07 | 1.48E+07 | 5.22E+07 | 4.19E+07 | 6.42E+07 | 2.07E+07 | 1.62E+07 | 1.93E+07 | 4.81E-04 | 2.82 | 2.91 | 1.03 |
| P01791 | Ig heavy chain V region HPCM6 |  | 1.42E+06 | 1.40E+06 | 2.44E+06 | 1.87E+07 | 1.74E+07 | 3.02E+07 | 1.59E+06 | 1.90E+06 | 4.64E+06 | 5.89E-04 | 8.14 | 12.59 | 1.55 |
| P12246 | Serum amyloid P-component | APCS | 7.48E+07 | 5.26E+07 | 9.80E+07 | 8.15E+08 | 4.68E+08 | 3.85E+08 | 7.53E+07 | 1.21E+08 | 7.22E+07 | 5.95E-04 | 6.21 | 7.40 | 1.19 |
| P01631 | Ig kappa chain V-II region 26-10 |  | 1.65E+08 | 1.25E+08 | 2.37E+08 | 1.20E+09 | 9.16E+08 | 6.80E+08 | 1.73E+08 | 2.74E+08 | 2.07E+08 | 6.83E-04 | 4.28 | 5.32 | 1.24 |
| P62806 | Histone H4 | H4F16 | 2.26E+07 | 3.32E+07 | 1.08E+08 | 1.08E+07 | 5.92E+06 | 2.74E+06 | 2.84E+08 | 2.67E+08 | 3.77E+08 | 6.92E-04 | 0.02 | 0.12 | 5.68 |
| P01632 | Ig kappa chain V-I region S107A | IGKV7-33 | 3.67E+07 | 3.01E+07 | 5.79E+07 | 2.96E+08 | 2.04E+08 | 1.68E+08 | 3.87E+07 | 6.18E+07 | 4.47E+07 | 7.03E-04 | 4.60 | 5.36 | 1.16 |
| P01844 | Ig lambda-2 chain C region | IGLC2 | 4.49E+06 | 2.15E+06 | 6.61E+06 | 6.58E+07 | 4.69E+07 | 4.02E+07 | 5.59E+06 | 1.10E+07 | 6.37E+06 | 7.41E-04 | 6.67 | 11.54 | 1.73 |
| P01843 | Ig lambda-1 chain C region |  | 1.62E+07 | 1.10E+07 | 2.53E+07 | 2.14E+08 | 1.53E+08 | 9.50E+07 | 1.79E+07 | 3.08E+07 | 1.97E+07 | 7.77E-04 | 6.76 | 8.82 | 1.30 |
| O70362 | Phosphatidylinositol-glycan-specific phospholipase D | GPLD1 | 1.14E+07 | 9.88E+06 | 7.27E+06 | 4.88E+07 | 1.22E+08 | 1.24E+08 | 2.28E+07 | 2.13E+07 | 1.34E+07 | 8.37E-04 | 5.12 | 10.31 | 2.01 |
| A6X935 | Inter alpha-trypsin inhibitor, heavy chain 4 | ITIH4 | 3.11E+08 | 2.13E+08 | 1.33E+08 | 7.99E+08 | 1.30E+09 | 9.49E+08 | 2.10E+08 | 2.52E+08 | 2.22E+08 | 9.29E-04 | 4.46 | 4.64 | 1.04 |
| P28666 | Murinoglobulin-2 | MUG2 | 1.89E+07 | 2.25E+07 | 1.39E+07 | 1.89E+08 | 5.36E+08 | 1.01E+09 | 6.42E+07 | 5.60E+07 | 3.30E+07 | 9.30E-04 | 11.33 | 31.38 | 2.77 |
| P01630 | Ig kappa chain V-II region 7S34.1 |  | 1.31E+07 | 9.57E+06 | 2.26E+07 | 1.55E+08 | 1.18E+08 | 7.31E+07 | 1.52E+07 | 2.51E+07 | 1.65E+07 | 9.47E-04 | 6.11 | 7.66 | 1.25 |
| P01638 | Ig kappa chain V-V region L6 (Fragment) |  | 1.61E+07 | 1.33E+07 | 3.18E+07 | 2.40E+08 | 1.40E+08 | 1.09E+08 | 2.34E+07 | 3.65E+07 | 2.36E+07 | 1.12E-03 | 5.86 | 8.00 | 1.36 |
| Q923D2 | Flavin reductase (NADPH) | BLVRB | 6.19E+05 | 2.47E+05 | 2.79E+05 | 3.82E+06 | 2.80E+06 | 2.15E+06 | 5.05E+05 | 7.57E+05 | 4.14E+05 | 1.13E-03 | 5.23 | 7.66 | 1.46 |
| P01635 | Immunoglobulin kappa chain variable 12-41 (Fragment) | IGKV12-41 | 8.63E+07 | 7.01E+07 | 1.46E+08 | 7.71E+08 | 5.26E+08 | 4.20E+08 | 9.16E+07 | 1.64E+08 | 1.20E+08 | 1.18E-03 | 4.57 | 5.69 | 1.24 |
| P01728 | Ig lambda-2 chain V region |  | 3.43E+06 | 3.42E+06 | 7.00E+06 | 3.59E+07 | 2.29E+07 | 1.83E+07 | 5.11E+06 | 5.97E+06 | 6.76E+06 | 1.20E-03 | 4.33 | 5.57 | 1.29 |
| P84750 | Ig kappa chain V region Mem5 (Fragment) |  | 7.60E+06 | 5.04E+06 | 1.32E+07 | 8.51E+07 | 6.07E+07 | 4.46E+07 | 7.69E+06 | 1.41E+07 | 1.14E+07 | 1.20E-03 | 5.74 | 7.36 | 1.28 |
| Q60994 | Adiponectin | ADIPOQ | 1.43E+07 | 2.63E+07 | 8.52E+06 | 1.34E+08 | 7.88E+07 | 1.32E+08 | 1.58E+07 | 2.25E+07 | 1.61E+07 | 1.23E-03 | 6.32 | 7.01 | 1.11 |
| Q07968 | Coagulation factor XIII B chain | F13B | 8.44E+05 | 1.28E+06 | 2.97E+05 | 1.79E+07 | 9.09E+06 | 2.23E+07 | 2.29E+06 | 1.01E+06 | 1.49E+06 | 1.27E-03 | 10.28 | 20.39 | 1.98 |
| Q60590 | Alpha-1-acid glycoprotein 1 | ORM1 | 1.66E+07 | 1.48E+07 | 2.92E+07 | 1.62E+08 | 1.61E+08 | 2.89E+08 | 2.22E+07 | 1.69E+07 | 5.14E+07 | 1.31E-03 | 6.76 | 10.08 | 1.49 |
| P09103 | Protein disulfide-isomerase | P4HB | 4.73E+05 | 1.61E+06 | 1.93E+06 | 3.07E+05 | 4.67E+05 | 7.23E+05 | 1.10E+07 | 7.18E+06 | 7.93E+06 | 1.32E-03 | 0.06 | 0.37 | 6.51 |
| P08607 | C4b-binding protein | C4BPA | 1.67E+07 | 2.38E+07 | 1.01E+07 | 1.42E+08 | 8.89E+07 | 2.30E+08 | 4.15E+07 | 2.69E+07 | 2.94E+07 | 1.34E-03 | 4.71 | 9.09 | 1.93 |
| Q61702 | Inter-alpha-trypsin inhibitor heavy chain H1 | ITIH1 | 1.13E+07 | 1.59E+07 | 4.90E+06 | 1.21E+08 | 2.11E+08 | 1.91E+08 | 3.45E+07 | 2.87E+07 | 1.12E+07 | 1.36E-03 | 7.04 | 16.36 | 2.32 |
| Q07456 | Protein AMBP | AMBP | 1.78E+07 | 1.47E+07 | 8.54E+06 | 8.29E+07 | 1.98E+08 | 2.28E+08 | 3.59E+07 | 2.90E+07 | 1.60E+07 | 1.42E-03 | 6.30 | 12.40 | 1.97 |
| Q9JJN5 | Carboxypeptidase N catalytic chain | CPN1 | 2.22E+06 | 3.00E+06 | 7.65E+06 | 5.05E+07 | 4.69E+07 | 6.89E+07 | 4.72E+06 | 5.74E+06 | 1.33E+07 | 1.48E-03 | 7.00 | 12.92 | 1.85 |
| Q80YC5 | Coagulation factor XII | F12 | 4.67E+06 | 2.43E+06 | 1.14E+06 | 2.94E+07 | 2.88E+07 | 4.49E+07 | 3.13E+06 | 3.06E+06 | 6.77E+06 | 1.49E-03 | 7.95 | 12.50 | 1.57 |
| P06909 | Complement factor H | CFH | 6.63E+07 | 9.01E+07 | 4.68E+07 | 5.13E+08 | 1.24E+09 | 2.31E+09 | 2.20E+08 | 1.79E+08 | 1.02E+08 | 1.54E-03 | 8.11 | 19.96 | 2.46 |
| P01837 | Immunoglobulin kappa constant | IGKC | 6.82E+08 | 7.05E+08 | 1.44E+09 | 4.76E+09 | 3.84E+09 | 3.28E+09 | 9.31E+08 | 1.28E+09 | 7.98E+08 | 1.56E-03 | 3.95 | 4.21 | 1.07 |
| P01636 | Ig kappa chain V-V region MOPC 149 |  | 3.97E+07 | 2.83E+07 | 6.64E+07 | 4.10E+08 | 2.67E+08 | 1.96E+08 | 4.32E+07 | 7.80E+07 | 5.27E+07 | 1.62E-03 | 5.02 | 6.50 | 1.29 |
| O08742 | Platelet glycoprotein V | GP5 | 2.35E+05 | 6.35E+05 | 1.77E+05 | 5.36E+06 | 3.19E+06 | 7.86E+06 | 1.40E+06 | 6.22E+05 | 8.22E+05 | 1.64E-03 | 5.77 | 15.68 | 2.72 |
| P48678 | Prelamin-A/C | LMNA | 3.67E+07 | 9.70E+07 | 1.01E+07 | 2.28E+05 | 6.54E+05 | 8.63E+05 | 1.37E+07 | 1.16E+07 | 2.93E+07 | 1.67E-03 | 0.03 | 0.01 | 0.38 |
| Q9DBD0 | Inhibitor of carbonic anhydrase | ICA | 2.62E+07 | 4.07E+07 | 1.09E+07 | 5.07E+08 | 2.68E+08 | 7.64E+08 | 9.43E+07 | 3.47E+07 | 4.63E+07 | 1.69E-03 | 8.78 | 19.79 | 2.25 |
| Q921I1 | Serotransferrin | TF | 8.14E+09 | 1.13E+10 | 4.70E+09 | 9.07E+10 | 5.01E+10 | 1.25E+11 | 2.52E+10 | 1.03E+10 | 1.39E+10 | 1.71E-03 | 5.37 | 11.00 | 2.05 |
| P29788 | Vitronectin | VTN | 2.36E+07 | 1.32E+07 | 9.25E+06 | 9.30E+07 | 1.29E+08 | 9.23E+07 | 2.48E+07 | 2.32E+07 | 1.07E+07 | 1.76E-03 | 5.34 | 6.81 | 1.27 |
| Q61704 | Inter-alpha-trypsin inhibitor heavy chain H3 | ITIH3 | 2.63E+07 | 2.82E+07 | 1.56E+07 | 1.33E+08 | 3.77E+08 | 5.38E+08 | 6.27E+07 | 6.19E+07 | 3.65E+07 | 1.86E-03 | 6.51 | 14.96 | 2.30 |
| P01642 | Ig kappa chain V-V region L7 (Fragment) | GM10881 | 6.05E+06 | 3.57E+06 | 9.95E+06 | 7.44E+07 | 4.68E+07 | 3.87E+07 | 6.50E+06 | 1.43E+07 | 7.59E+06 | 1.88E-03 | 5.63 | 8.17 | 1.45 |
| P28665 | Murinoglobulin-1 | MUG1 | 3.57E+08 | 3.87E+08 | 2.69E+08 | 1.99E+09 | 5.40E+09 | 1.00E+10 | 9.73E+08 | 8.51E+08 | 5.60E+08 | 1.92E-03 | 7.30 | 17.20 | 2.36 |
| P97350 | Plakophilin-1 | PKP1 | 2.25E+06 | 2.63E+06 | Not detected | Not detected | Not detected | Not detected | 3.88E+07 | 3.99E+07 | 5.59E+07 | 1.93E-03 | – | – | 18.40 |
| Q8VCG4 | Complement component C8 gamma chain | C8G | 3.41E+06 | 2.26E+06 | 6.16E+06 | 3.83E+07 | 2.30E+07 | 1.74E+07 | 4.84E+06 | 5.57E+06 | 3.37E+06 | 1.97E-03 | 5.71 | 6.65 | 1.16 |
| Q8CG16 | Complement C1r-A subcomponent | C1RA | 4.02E+06 | 3.72E+06 | 1.61E+06 | 1.32E+07 | 2.69E+07 | 2.01E+07 | 3.93E+06 | 4.45E+06 | 5.82E+06 | 2.07E-03 | 4.24 | 6.43 | 1.52 |
| Q61703 | Inter-alpha-trypsin inhibitor heavy chain H2 | ITIH2 | 2.50E+07 | 2.44E+07 | 1.46E+07 | 1.25E+08 | 3.87E+08 | 2.98E+08 | 6.08E+07 | 5.31E+07 | 2.28E+07 | 2.20E-03 | 5.92 | 12.67 | 2.14 |
| Q61147 | Ceruloplasmin | CP | 1.60E+08 | 1.99E+08 | 1.16E+08 | 9.74E+08 | 2.80E+09 | 5.03E+09 | 5.00E+08 | 4.20E+08 | 2.50E+08 | 2.33E-03 | 7.53 | 18.50 | 2.46 |
| Q61838 | Pregnancy zone protein | PZP | 4.07E+09 | 5.65E+09 | 2.56E+09 | 1.66E+10 | 2.62E+10 | 5.23E+10 | 6.75E+09 | 6.26E+09 | 4.81E+09 | 2.58E-03 | 5.34 | 7.75 | 1.45 |
| P01807 | Ig heavy chain V region X44 |  | 5.15E+07 | 5.04E+07 | 1.11E+08 | 3.72E+08 | 4.88E+08 | 8.60E+08 | 5.65E+07 | 6.45E+07 | 1.45E+08 | 2.64E-03 | 6.46 | 8.08 | 1.25 |
| P00920 | Carbonic anhydrase 2 | CA2 | 3.20E+06 | 1.92E+06 | 3.89E+06 | 4.37E+07 | 2.44E+07 | 1.74E+07 | 4.06E+06 | 7.33E+06 | 2.18E+06 | 2.92E-03 | 6.30 | 9.49 | 1.51 |
| P01746 | Ig heavy chain V region 93G7 |  | 9.21E+06 | 1.31E+07 | 2.16E+07 | 9.31E+07 | 1.22E+08 | 1.99E+08 | 1.05E+07 | 1.25E+07 | 3.63E+07 | 2.95E-03 | 6.97 | 9.42 | 1.35 |
| Q8BND5 | Sulfhydryl oxidase 1 | QSOX1 | 1.58E+07 | 1.11E+07 | 5.35E+06 | 6.89E+07 | 7.06E+07 | 6.06E+07 | 1.95E+07 | 1.11E+07 | 7.00E+06 | 3.04E-03 | 5.32 | 6.21 | 1.17 |
| P23953 | Carboxylesterase 1C | CES1C | 4.60E+08 | 4.57E+08 | 2.13E+08 | 3.25E+09 | 1.85E+09 | 5.03E+09 | 1.08E+09 | 4.46E+08 | 6.40E+08 | 3.19E-03 | 4.67 | 8.97 | 1.92 |
| P01723 | Ig lambda-1 chain V region |  | 8.50E+06 | 6.32E+06 | 1.57E+07 | 8.56E+07 | 5.14E+07 | 4.50E+07 | 1.11E+07 | 2.02E+07 | 9.29E+06 | 3.23E-03 | 4.48 | 5.96 | 1.33 |
| Q62351 | Transferrin receptor protein 1 | TFRC | 1.38E+06 | 4.87E+05 | 8.72E+05 | 7.60E+06 | 2.68E+07 | 6.94E+06 | 9.34E+05 | 1.92E+06 | 1.57E+06 | 3.23E-03 | 9.36 | 15.12 | 1.62 |
| Q91Y97 | Fructose-bisphosphate aldolase B | ALDOB | 2.39E+05 | 3.77E+05 | 1.14E+05 | 1.90E+06 | 1.13E+06 | 2.20E+06 | 4.41E+05 | 2.45E+05 | 1.85E+05 | 3.63E-03 | 5.99 | 7.15 | 1.19 |
| P20918 | Plasminogen | PLG | 1.79E+08 | 7.59E+07 | 1.23E+08 | 7.21E+08 | 2.05E+09 | 5.68E+08 | 8.59E+07 | 1.66E+08 | 1.36E+08 | 3.68E-03 | 8.60 | 8.85 | 1.03 |
| O70456 | 14-3-3 protein sigma | SFN | 2.10E+07 | 6.09E+07 | 1.03E+08 | 1.84E+07 | 1.19E+07 | 1.27E+07 | 1.37E+08 | 1.24E+08 | 1.57E+08 | 3.74E-03 | 0.10 | 0.23 | 2.26 |
| Q60930 | Voltage-dependent anion-selective channel protein 2 | VDAC2 | Not detected | 8.89E+05 | 3.02E+05 | Not detected | Not detected | Not detected | 4.48E+06 | 8.39E+06 | 1.36E+07 | 3.78E-03 | – | – | 14.83 |
| P01801 | Ig heavy chain V-III region J606 |  | 2.33E+07 | 2.21E+07 | 4.38E+07 | 1.72E+08 | 1.96E+08 | 3.12E+08 | 2.37E+07 | 2.75E+07 | 8.10E+07 | 3.91E-03 | 5.15 | 7.64 | 1.48 |
| P68372 | Tubulin beta-4B chain | TUBB4B | 3.95E+06 | 9.94E+06 | 2.11E+07 | 2.90E+06 | 2.90E+06 | 4.57E+06 | 3.60E+07 | 3.16E+07 | 5.98E+07 | 3.97E-03 | 0.08 | 0.30 | 3.64 |
| P01633 | Immunoglobulin kappa chain variable 6-17 | IGKV6-17 | 1.26E+08 | 8.51E+07 | 2.14E+08 | 1.26E+09 | 6.54E+08 | 4.96E+08 | 1.57E+08 | 2.23E+08 | 1.82E+08 | 3.97E-03 | 4.28 | 5.68 | 1.33 |
| O88947 | Coagulation factor X | F10 | 4.43E+06 | 4.94E+06 | 8.05E+06 | 3.92E+07 | 4.43E+07 | 9.25E+07 | 6.71E+06 | 7.13E+06 | 2.26E+07 | 3.99E-03 | 4.83 | 10.10 | 2.09 |
| P32261 | Antithrombin-III | SERPINC1 | 9.22E+07 | 7.55E+07 | 5.82E+07 | 2.39E+08 | 3.42E+08 | 1.64E+08 | 7.88E+07 | 9.30E+07 | 5.54E+07 | 4.11E-03 | 3.28 | 3.29 | 1.01 |
| P01660 | Ig kappa chain V-III region PC 3741/TEPC 111 |  | 1.24E+07 | 8.10E+06 | 2.15E+07 | 1.11E+08 | 7.34E+07 | 5.79E+07 | 1.58E+07 | 2.86E+07 | 1.28E+07 | 4.12E-03 | 4.24 | 5.78 | 1.36 |
| Q9ESB3 | Histidine-rich glycoprotein | HRG | 2.08E+08 | 1.88E+08 | 1.06E+08 | 4.68E+08 | 3.20E+08 | 5.55E+08 | 1.52E+08 | 1.39E+08 | 1.44E+08 | 4.21E-03 | 3.09 | 2.68 | 0.87 |
| P46412 | Glutathione peroxidase 3 | GPX3 | 2.62E+06 | 3.51E+06 | 8.41E+06 | 2.45E+07 | 2.37E+07 | 1.42E+07 | 4.04E+06 | 4.07E+06 | 4.74E+06 | 4.27E-03 | 4.86 | 4.29 | 0.88 |
| P26262 | Plasma kallikrein | KLKB1 | 5.26E+06 | 3.13E+06 | 9.94E+06 | 3.55E+07 | 5.52E+07 | 8.25E+07 | 5.25E+06 | 4.61E+06 | 1.48E+07 | 4.29E-03 | 7.02 | 9.45 | 1.35 |
| Q8R121 | Protein Z-dependent protease inhibitor | SERPINA10 | 5.24E+06 | 1.42E+06 | 1.05E+06 | 1.39E+07 | 1.74E+07 | 2.32E+07 | 2.50E+06 | 1.55E+06 | 2.85E+06 | 4.42E-03 | 7.89 | 7.06 | 0.90 |
| P04186 | Complement factor B | CFB | 7.29E+07 | 3.13E+07 | 3.68E+07 | 2.22E+08 | 5.10E+08 | 1.55E+08 | 3.45E+07 | 5.28E+07 | 4.68E+07 | 4.42E-03 | 6.61 | 6.29 | 0.95 |
| P01027 | Complement C3 | C3 | 4.41E+09 | 3.59E+09 | 1.99E+09 | 1.61E+10 | 1.30E+10 | 1.19E+10 | 4.43E+09 | 4.47E+09 | 2.03E+09 | 4.56E-03 | 3.75 | 4.10 | 1.09 |
| Q03734 | Serine protease inhibitor A3M | SERPINA3M | 1.04E+07 | 6.79E+06 | 5.91E+06 | 2.78E+07 | 4.57E+07 | 2.47E+07 | 9.52E+06 | 3.43E+06 | 7.33E+06 | 4.60E-03 | 4.84 | 4.26 | 0.88 |
| P01867 | Immunoglobulin heavy constant gamma 2B | IGHG2B | 6.98E+08 | 7.66E+08 | 1.92E+09 | 6.98E+09 | 8.99E+09 | 1.46E+10 | 8.66E+08 | 9.39E+08 | 2.96E+09 | 4.77E-03 | 6.41 | 9.03 | 1.41 |
| P20152 | Vimentin | VIM | 6.79E+07 | 2.14E+08 | 1.50E+08 | 2.74E+07 | 1.60E+07 | 2.32E+07 | 7.30E+07 | 9.07E+07 | 5.15E+07 | 4.78E-03 | 0.31 | 0.15 | 0.50 |
| P01869 | Ig gamma-1 chain C region, membrane-bound form | IGHG1 | 5.97E+08 | 5.28E+08 | 1.33E+09 | 4.79E+09 | 5.89E+09 | 9.48E+09 | 6.59E+08 | 7.58E+08 | 2.23E+09 | 4.79E-03 | 5.53 | 8.20 | 1.48 |
| P18527 | Ig heavy chain V region 914 |  | 4.98E+06 | 3.35E+06 | 9.83E+06 | 4.50E+07 | 4.06E+07 | 7.71E+07 | 4.31E+06 | 5.77E+06 | 1.67E+07 | 5.04E-03 | 6.07 | 8.96 | 1.48 |
| P01865 | Ig gamma-2A chain C region, membrane-bound form | IGH-1A | 1.13E+09 | 1.18E+09 | 2.69E+09 | 9.19E+09 | 1.11E+10 | 1.77E+10 | 1.19E+09 | 1.33E+09 | 4.07E+09 | 5.06E-03 | 5.77 | 7.61 | 1.32 |
| P01864 | Ig gamma-2A chain C region secreted form |  | 4.72E+08 | 5.26E+08 | 1.22E+09 | 4.33E+09 | 5.17E+09 | 7.16E+09 | 4.67E+08 | 5.96E+08 | 1.79E+09 | 5.22E-03 | 5.84 | 7.51 | 1.29 |
| P18531 | Ig heavy chain V region 3-6 | IGHV3-6 | 3.28E+06 | 2.85E+06 | 8.60E+06 | 3.98E+07 | 4.11E+07 | 8.46E+07 | 4.04E+06 | 4.59E+06 | 1.60E+07 | 5.40E-03 | 6.71 | 11.23 | 1.67 |
| P49182 | Heparin cofactor 2 | SERPIND1 | 1.59E+07 | 9.24E+06 | 3.56E+06 | 1.03E+08 | 6.64E+07 | 9.42E+07 | 2.69E+07 | 1.33E+07 | 8.21E+06 | 5.45E-03 | 5.45 | 9.21 | 1.69 |
| Q6GQT1 | Alpha-2-macroglobulin-P | A2M | 2.04E+05 | 3.20E+05 | 4.96E+04 | 1.91E+06 | 5.48E+06 | 9.95E+06 | 9.30E+05 | 6.32E+05 | 3.03E+05 | 5.56E-03 | 9.30 | 30.25 | 3.25 |
| Q64726 | Zinc-alpha-2-glycoprotein | AZGP1 | 1.11E+07 | 6.86E+06 | 2.02E+07 | 8.94E+07 | 9.67E+07 | 1.62E+08 | 2.50E+07 | 1.11E+07 | 4.49E+07 | 5.82E-03 | 4.30 | 9.14 | 2.12 |
| Q8VCS0 | N-acetylmuramoyl-L-alanine amidase | PGLYRP2 | 1.52E+06 | 1.93E+06 | 4.77E+05 | 1.79E+07 | 9.50E+06 | 2.24E+07 | 5.70E+06 | 1.79E+06 | 1.85E+06 | 5.90E-03 | 5.34 | 12.67 | 2.37 |
| P06683 | Complement component C9 | C9 | 3.31E+05 | 5.62E+05 | 2.47E+05 | 5.03E+06 | 5.24E+06 | 7.82E+06 | 3.13E+06 | 4.14E+05 | 1.73E+06 | 6.07E-03 | 3.43 | 15.87 | 4.63 |
| P70274 | Selenoprotein P | SELENOP | 3.02E+05 | 2.24E+05 | 4.81E+05 | 2.65E+06 | 3.98E+06 | 9.22E+06 | 4.34E+05 | 3.49E+05 | 1.71E+06 | 6.20E-03 | 6.37 | 15.71 | 2.47 |
| Q06890 | Clusterin | CLU | 3.48E+07 | 4.15E+07 | 1.99E+07 | 8.39E+07 | 7.20E+07 | 1.41E+08 | 3.45E+07 | 3.19E+07 | 3.09E+07 | 6.25E-03 | 3.06 | 3.09 | 1.01 |
| P17182 | Alpha-enolase | ENO1 | 1.78E+06 | 2.21E+05 | 4.80E+06 | 2.49E+05 | Not detected | Not detected | 3.48E+07 | 1.08E+07 | 1.03E+07 | 6.78E-03 | 0.01 | 0.11 | 8.23 |
| P10126 | Elongation factor 1-alpha 1 | EEF1A1 | 2.36E+07 | 5.98E+07 | 1.60E+08 | 1.48E+07 | 7.21E+06 | 1.01E+07 | 9.09E+07 | 1.15E+08 | 1.57E+08 | 6.82E-03 | 0.09 | 0.13 | 1.49 |
| Q8CG14 | Complement C1s-1 subcomponent | C1S1 | 3.91E+06 | 1.02E+06 | 1.78E+06 | 8.86E+06 | 3.30E+07 | 1.56E+07 | 2.87E+06 | 3.29E+06 | 2.16E+06 | 6.83E-03 | 6.90 | 8.55 | 1.24 |
| P07361 | Alpha-1-acid glycoprotein 2 | ORM2 | 1.39E+06 | 1.32E+06 | 7.32E+06 | 2.39E+07 | 2.68E+07 | 3.00E+07 | 3.47E+06 | 2.30E+06 | 5.93E+06 | 6.97E-03 | 6.90 | 8.05 | 1.17 |
| P13020 | Gelsolin | GSN | 1.03E+08 | 4.51E+07 | 8.13E+07 | 3.08E+08 | 9.56E+08 | 3.03E+08 | 4.52E+07 | 9.41E+07 | 9.19E+07 | 7.27E-03 | 6.77 | 6.81 | 1.01 |
| O08677 | Kininogen-1 | KNG1 | 3.06E+08 | 1.87E+08 | 1.32E+08 | 5.33E+08 | 7.16E+08 | 5.38E+08 | 2.46E+08 | 2.30E+08 | 1.47E+08 | 7.48E-03 | 2.87 | 2.86 | 1.00 |
| Q61129 | Complement factor I | CFI | 9.47E+06 | 9.91E+06 | 1.64E+07 | 4.94E+07 | 1.13E+08 | 7.01E+07 | 7.60E+06 | 1.60E+07 | 2.94E+07 | 7.58E-03 | 4.39 | 6.50 | 1.48 |
| O88968 | Transcobalamin-2 | TCN2 | Not detected | Not detected | Not detected | 6.15E+05 | 8.90E+05 | 9.99E+05 | 3.17E+05 | Not detected | Not detected | 7.76E-03 | 2.63 | – | – |
| P02089 | Hemoglobin subunit beta-2 | HBB-B2 | 1.86E+06 | 2.78E+06 | 4.16E+06 | 1.22E+07 | 1.07E+07 | 6.70E+06 | 4.08E+06 | 3.38E+06 | 4.92E+06 | 7.80E-03 | 2.39 | 3.36 | 1.41 |
| P97290 | Plasma protease C1 inhibitor | SERPING1 | 7.84E+07 | 2.34E+07 | 3.17E+07 | 1.51E+08 | 4.19E+08 | 1.92E+08 | 3.77E+07 | 5.20E+07 | 5.19E+07 | 7.93E-03 | 5.38 | 5.71 | 1.06 |
| P01670 | Ig kappa chain V-III region PC 6684 |  | 1.24E+07 | 3.56E+06 | 1.14E+07 | 9.20E+07 | 5.35E+07 | 4.97E+07 | 9.29E+06 | 2.39E+07 | 1.77E+07 | 8.13E-03 | 3.84 | 7.13 | 1.86 |
| Q02257 | Junction plakoglobin | JUP | 6.50E+06 | 1.01E+07 | 1.10E+05 | 1.28E+05 | 4.02E+04 | 1.30E+05 | 3.00E+07 | 3.27E+07 | 3.47E+07 | 8.33E-03 | 0.00 | 0.02 | 5.84 |
| Q01339 | Beta-2-glycoprotein 1 | APOH | 7.95E+07 | 9.25E+07 | 1.53E+08 | 3.66E+08 | 6.06E+08 | 4.07E+08 | 4.89E+07 | 7.75E+07 | 1.78E+08 | 8.38E-03 | 4.53 | 4.24 | 0.94 |
| P11859 | Angiotensinogen | AGT | 3.35E+06 | 2.71E+06 | 8.28E+06 | 3.24E+07 | 4.16E+07 | 4.19E+07 | 2.54E+06 | 3.87E+06 | 1.38E+07 | 8.47E-03 | 5.74 | 8.08 | 1.41 |
| P21614 | Vitamin D-binding protein | GC | 1.50E+08 | 1.39E+08 | 3.96E+08 | 1.28E+09 | 1.85E+09 | 2.69E+09 | 1.41E+08 | 2.01E+08 | 6.94E+08 | 9.66E-03 | 5.61 | 8.49 | 1.51 |
| P03987 | Ig gamma-3 chain C region |  | 1.37E+08 | 1.21E+08 | 3.66E+08 | 1.15E+09 | 1.46E+09 | 2.15E+09 | 1.34E+08 | 1.60E+08 | 5.95E+08 | 1.03E-02 | 5.35 | 7.63 | 1.43 |
| P97298 | Pigment epithelium-derived factor | SERPINF1 | 3.38E+06 | 2.45E+06 | 6.49E+06 | 2.99E+07 | 3.71E+07 | 6.04E+07 | 3.72E+06 | 4.41E+06 | 2.07E+07 | 1.04E-02 | 4.41 | 10.34 | 2.34 |
| P18524 | Ig heavy chain V region RF |  | 3.19E+07 | 2.88E+07 | 6.94E+07 | 1.98E+08 | 2.66E+08 | 3.86E+08 | 3.11E+07 | 3.64E+07 | 1.23E+08 | 1.05E-02 | 4.45 | 6.54 | 1.47 |
| Q8VCM7 | Fibrinogen gamma chain | FGG | Not detected | Not detected | 3.25E+06 | 4.67E+05 | Not detected | Not detected | 7.57E+06 | 7.34E+06 | 1.15E+07 | 1.07E-02 | 0.05 | 0.14 | 2.72 |
| P11276 | Fibronectin | FN1 | 4.63E+07 | 5.56E+07 | 1.40E+07 | 2.38E+08 | 1.04E+09 | 1.02E+09 | 8.26E+07 | 1.20E+08 | 2.16E+07 | 1.09E-02 | 10.30 | 19.88 | 1.93 |
| P07356 | Annexin A2 | ANXA2 | 1.94E+07 | 4.34E+07 | 1.37E+08 | 2.11E+07 | 1.72E+07 | 1.36E+07 | 1.45E+08 | 1.40E+08 | 1.79E+08 | 1.09E-02 | 0.11 | 0.26 | 2.31 |
| Q9Z126 | Platelet factor 4 | PF4 | 1.86E+07 | 2.58E+07 | 3.36E+07 | 1.41E+08 | 1.37E+08 | 5.30E+07 | 2.80E+07 | 2.65E+07 | 4.54E+07 | 1.20E-02 | 3.31 | 4.24 | 1.28 |
| Q00724 | Retinol-binding protein 4 | RBP4 | 6.20E+06 | 8.30E+06 | 2.34E+07 | 6.25E+07 | 4.03E+07 | 3.70E+07 | 1.10E+07 | 1.10E+07 | 1.46E+07 | 1.31E-02 | 3.81 | 3.68 | 0.97 |
| Q8BTM8 | Filamin-A | FLNA | Not detected | 5.66E+04 | 3.06E+04 | 3.11E+05 | 7.09E+05 | 1.66E+06 | Not detected | 1.66E+05 | Not detected | 1.34E-02 | 5.37 | 20.46 | 3.81 |
| P42703 | Leukemia inhibitory factor receptor | LIFR | 1.65E+06 | 1.12E+06 | 6.78E+05 | 3.39E+06 | 8.66E+06 | 1.41E+07 | 2.76E+06 | 2.15E+06 | 1.08E+06 | 1.44E-02 | 4.36 | 7.57 | 1.74 |
| P11247 | Myeloperoxidase | MPO | 8.11E+05 | 3.64E+05 | 1.69E+07 | 2.65E+06 | Not detected | Not detected | 1.34E+08 | 4.80E+07 | 5.98E+07 | 1.47E-02 | 0.03 | 0.44 | 13.33 |
| P11680 | Properdin | CFP | 2.41E+06 | 2.05E+06 | 4.34E+06 | 1.73E+07 | 3.26E+07 | 2.74E+07 | 1.92E+06 | 3.68E+06 | 1.46E+07 | 1.63E-02 | 3.82 | 8.77 | 2.29 |
| P06330 | Ig heavy chain V region AC38 205.12 |  | 2.29E+08 | 2.74E+08 | 6.13E+08 | 1.21E+09 | 2.28E+09 | 3.54E+09 | 2.79E+08 | 2.46E+08 | 9.08E+08 | 1.71E-02 | 4.90 | 6.30 | 1.28 |
| P70389 | Insulin-like growth factor-binding protein complex acid labile subunit | IGFALS | 6.86E+06 | 1.20E+06 | 2.59E+06 | 1.38E+07 | 5.29E+07 | 1.92E+07 | 1.77E+06 | 4.58E+06 | 4.15E+06 | 1.72E-02 | 8.19 | 8.07 | 0.99 |
| P06336 | Ig epsilon chain C region |  | 3.35E+06 | 5.36E+05 | 1.24E+06 | 8.24E+06 | 2.82E+07 | 8.26E+06 | 1.30E+06 | 2.53E+06 | 2.52E+06 | 1.73E-02 | 7.04 | 8.73 | 1.24 |
| Q91X72 | Hemopexin | HPX | 5.83E+09 | 4.44E+09 | 2.22E+09 | 1.52E+10 | 1.03E+10 | 2.17E+10 | 7.70E+09 | 5.26E+09 | 3.59E+09 | 1.74E-02 | 2.85 | 3.78 | 1.32 |
| Q03311 | Cholinesterase | BCHE | 2.84E+05 | Not detected | Not detected | 6.73E+05 | 2.42E+06 | 7.68E+05 | 9.57E+04 | 2.07E+05 | 2.29E+05 | 1.96E-02 | 7.27 | 4.54 | 0.62 |
| Q9QWK4 | CD5 antigen-like | CD5L | 1.96E+07 | 1.76E+07 | 4.54E+07 | 1.42E+08 | 2.07E+08 | 1.27E+08 | 1.15E+07 | 2.54E+07 | 7.32E+07 | 2.08E-02 | 4.32 | 5.76 | 1.33 |
| P07309 | Transthyretin | TTR | 6.17E+08 | 6.98E+08 | 8.07E+08 | 1.68E+09 | 1.46E+09 | 1.11E+09 | 7.75E+08 | 6.31E+08 | 1.13E+09 | 2.16E-02 | 1.67 | 2.00 | 1.20 |
| Q06770 | Corticosteroid-binding globulin | SERPINA6 | 2.13E+07 | 1.19E+07 | 6.55E+06 | 6.35E+07 | 8.05E+07 | 4.41E+07 | 1.87E+07 | 1.82E+07 | 4.72E+06 | 2.24E-02 | 4.52 | 4.73 | 1.04 |
| P26039 | Talin-1 | TLN1 | 5.44E+04 | Not detected | Not detected | 2.32E+05 | 2.32E+06 | 3.62E+06 | 1.31E+05 | 6.84E+04 | Not detected | 2.39E-02 | 20.60 | 37.86 | 1.84 |
| Q9D2Q8 | Protein S100-A14 | S100A14 | 4.64E+05 | Not detected | 1.60E+07 | 9.17E+05 | Not detected | 1.54E+05 | 2.54E+07 | 1.91E+07 | 2.21E+07 | 2.40E-02 | 0.02 | 0.07 | 2.71 |
| O89020 | Afamin | AFM | 9.84E+07 | 2.24E+07 | 3.15E+07 | 1.23E+08 | 2.30E+08 | 1.57E+08 | 3.68E+07 | 4.16E+07 | 5.77E+07 | 2.53E-02 | 3.75 | 3.35 | 0.89 |
| Q9DBB9 | Carboxypeptidase N subunit 2 | CPN2 | 1.41E+07 | 2.31E+06 | 5.55E+06 | 2.23E+07 | 5.21E+07 | 2.28E+07 | 4.52E+06 | 5.86E+06 | 7.92E+06 | 2.61E-02 | 5.31 | 4.42 | 0.83 |
| P10107 | Annexin A1 | ANXA1 | 3.37E+07 | 2.20E+07 | 9.56E+08 | 6.51E+07 | 1.02E+07 | 2.65E+06 | 1.06E+09 | 1.20E+09 | 1.33E+09 | 2.74E-02 | 0.02 | 0.08 | 3.54 |
| P01749 | Ig heavy chain V region 3 | IGHV1-61 | 1.88E+06 | 2.21E+06 | 7.53E+06 | 1.84E+07 | 3.56E+07 | 5.13E+07 | 3.23E+06 | 2.44E+06 | 1.78E+07 | 2.93E-02 | 4.48 | 9.06 | 2.02 |
| E9Q414 | Apolipoprotein B-100 | APOB | 1.03E+07 | 2.23E+07 | 1.66E+07 | 3.48E+07 | 1.07E+08 | 2.01E+08 | 6.19E+07 | 5.79E+07 | 3.26E+07 | 2.94E-02 | 2.25 | 6.95 | 3.09 |
| P01887 | Beta-2-microglobulin | B2M | 6.30E+06 | 1.35E+07 | 1.46E+07 | 4.89E+07 | 4.95E+07 | 2.04E+07 | 1.08E+07 | 1.24E+07 | 2.00E+07 | 3.12E-02 | 2.75 | 3.45 | 1.25 |
| P09581 | Macrophage colony-stimulating factor 1 receptor | CSF1R | 1.75E+05 | Not detected | Not detected | 8.09E+05 | 4.87E+06 | 6.92E+05 | 3.90E+04 | Not detected | 1.35E+05 | 3.15E-02 | 24.40 | 12.14 | 0.50 |
| P63017 | Heat shock cognate 71 kDa protein | HSPA8 | 3.59E+06 | 2.97E+07 | 1.43E+07 | 2.47E+06 | 3.48E+06 | 4.26E+06 | 1.82E+07 | 2.30E+07 | 2.06E+07 | 3.38E-02 | 0.17 | 0.21 | 1.30 |
| Q60963 | Platelet-activating factor acetylhydrolase | PLA2G7 | 1.34E+06 | 2.52E+05 | 4.30E+05 | 6.45E+06 | 5.38E+06 | 3.07E+06 | 2.18E+06 | 6.60E+05 | Not detected | 3.39E-02 | 3.50 | 7.35 | 2.10 |
| Q9JHH6 | Carboxypeptidase B2 | CPB2 | 1.61E+06 | 2.14E+06 | 7.63E+05 | 1.47E+07 | 1.17E+07 | 1.38E+07 | 3.53E+06 | 2.65E+06 | 2.29E+05 | 3.44E-02 | 6.26 | 8.91 | 1.42 |
| Q08879 | Fibulin-1 | FBLN1 | 1.86E+05 | Not detected | 9.35E+04 | 2.88E+05 | 1.81E+06 | 4.16E+05 | 9.09E+04 | 1.25E+05 | 1.24E+05 | 3.75E-02 | 7.40 | 5.99 | 0.81 |
| P21107 | Tropomyosin alpha-3 chain | TPM3 | 2.70E+07 | 1.41E+08 | 4.34E+08 | 1.09E+07 | 9.20E+06 | 1.37E+07 | 2.57E+07 | 3.72E+07 | 1.51E+07 | 3.78E-02 | 0.43 | 0.06 | 0.13 |
| P07901 | Heat shock protein HSP 90-alpha | HSP90AA1 | Not detected | 6.27E+04 | 4.41E+06 | Not detected | Not detected | Not detected | 5.74E+06 | 1.05E+07 | 1.28E+07 | 3.83E-02 | – | – | 4.33 |
| P82198 | Transforming growth factor-beta-induced protein ig-h3 | TGFBI | 1.69E+06 | 1.02E+06 | 3.30E+05 | 4.75E+06 | 4.86E+06 | 2.71E+06 | 1.34E+06 | 9.82E+05 | Not detected | 3.86E-02 | 3.54 | 4.06 | 1.14 |
| P07724 | Albumin | ALB | 1.41E+11 | 1.08E+11 | 5.09E+10 | 3.57E+11 | 3.90E+11 | 2.29E+11 | 1.52E+11 | 1.42E+11 | 5.27E+10 | 3.90E-02 | 2.81 | 3.25 | 1.15 |
| P20029 | Endoplasmic reticulum chaperone BiP | HSPA5 | 8.63E+06 | 5.04E+07 | 2.23E+07 | 3.59E+06 | 3.23E+06 | 5.52E+06 | 8.69E+06 | 1.57E+07 | 5.68E+06 | 4.22E-02 | 0.41 | 0.15 | 0.37 |
| P11499 | Heat shock protein HSP 90-beta | HSP90AB1 | 6.78E+06 | 2.02E+07 | 1.72E+07 | 2.09E+06 | 2.53E+06 | 5.02E+06 | 6.79E+06 | 1.44E+07 | 4.41E+06 | 4.25E-02 | 0.38 | 0.22 | 0.58 |
| P16301 | Phosphatidylcholine-sterol acyltransferase | LCAT | 9.58E+05 | 9.08E+05 | 1.65E+05 | 3.05E+06 | 1.54E+06 | 1.50E+06 | Not detected | Not detected | Not detected | 4.31E-02 | – | 3.00 | – |
| P31532 | Serum amyloid A-4 protein | SAA4 | 1.50E+07 | 2.04E+07 | 3.90E+07 | 8.50E+07 | 8.42E+07 | 3.77E+07 | 2.68E+07 | 2.88E+07 | 3.54E+07 | 4.42E-02 | 2.27 | 2.79 | 1.22 |
| Q01279 | Epidermal growth factor receptor | EGFR | 1.15E+07 | 2.53E+06 | 5.55E+06 | 1.70E+07 | 5.73E+07 | 1.70E+07 | 5.36E+06 | 7.04E+06 | 9.53E+06 | 4.61E-02 | 4.16 | 4.67 | 1.12 |
| P10605 | Cathepsin B | CTSB | Not detected | 7.93E+05 | Not detected | 3.56E+06 | 8.72E+05 | 5.75E+06 | 9.86E+05 | 3.34E+05 | 2.96E+05 | 4.68E-02 | 6.30 | 4.28 | 0.68 |
| P17742 | Peptidyl-prolyl cis-trans isomerase A | PPIA | Not detected | Not detected | 6.30E+07 | 4.52E+06 | Not detected | Not detected | 9.42E+07 | 8.65E+07 | 1.22E+08 | 4.70E-02 | 0.04 | 0.07 | 1.60 |
| P29699 | Alpha-2-HS-glycoprotein | AHSG | 1.29E+08 | 1.50E+08 | 1.68E+08 | 2.76E+08 | 5.02E+08 | 4.75E+08 | 1.26E+08 | 1.17E+08 | 3.51E+08 | 4.91E-02 | 2.11 | 2.80 | 1.33 |

**Table S5**. Proteins showing statistically significant changes (P-value < 0.05) in the samples pulled down with D-Cb in comparison to those pulled down with L-Cb by Student's t-test. ^a^'-' represents that the abundance ratios cannot be calculated because of absence of the peptide peaks in the samples pulled down with L-Cb. ^b^'O' represents the identified protein list as ligands of LRP1 and LRP2 (*Frontiers in Physiology* **2012**, 3, 269).

| **Accession No.** | **Protein description** | **Gene symbol** | ***P*-value (Student's *t*-test)** | **Abundance ratio (D-Cb/L-Cb)^a^** | **Ligands of LRP1 and LRP2^b^** |
| --- | --- | --- | --- | --- | --- |
| P16301 | Phosphatidylcholine-sterol acyltransferase | LCAT | 5.58E-03 | – |  |
| P09581 | Macrophage colony-stimulating factor 1 receptor | CSF1R | 2.72E-02 | 24.40 |  |
| P06684 | Complement C5 | C5 | 1.86E-04 | 11.65 |  |
| P28666 | Murinoglobulin-2 | MUG2 | 6.56E-03 | 11.33 |  |
| O88783 | Coagulation factor V | F5 | 2.20E-03 | 10.34 |  |
| P11276 | Fibronectin | FN1 | 1.49E-02 | 10.30 |  |
| Q07968 | Coagulation factor XIII B chain | F13B | 1.48E-03 | 10.28 |  |
| P35441 | Thrombospondin-1 | THBS1 | 2.09E-03 | 9.45 |  |
| Q62351 | Transferrin receptor protein 1 | TFRC | 6.51E-03 | 9.36 |  |
| Q6GQT1 | Alpha-2-macroglobulin-P (Alpha-2-macroglobulin) | A2M | 1.10E-02 | 9.30 |  |
| P14847 | C-reactive protein | CRP | 1.93E-03 | 9.17 |  |
| P98086 | Complement C1q subcomponent subunit A | C1QA | 5.58E-04 | 8.99 |  |
| P21180 | Complement C2 | C2 | 3.86E-04 | 8.98 |  |
| Q9DBD0 | Inhibitor of carbonic anhydrase | ICA | 3.46E-03 | 8.78 |  |
| P20918 | Plasminogen | PLG | 5.04E-03 | 8.60 | O |
| P01898 | H-2 class I histocompatibility antigen, Q10 alpha chain | H2-Q10 | 2.72E-04 | 8.54 |  |
| Q9R098 | Hepatocyte growth factor activator | HGFAC | 1.46E-04 | 8.31 |  |
| P70389 | Insulin-like growth factor-binding protein complex acid labile subunit | IGFALS | 8.12E-03 | 8.19 |  |
| P01791 | Ig heavy chain V region HPCM6 |  | 2.14E-03 | 8.14 |  |
| P06909 | Complement factor H | CFH | 8.10E-03 | 8.11 |  |
| Q80YC5 | Coagulation factor XII | F12 | 1.03E-03 | 7.95 |  |
| Q8R121 | Protein Z-dependent protease inhibitor | SERPINA10 | 4.66E-04 | 7.89 |  |
| P14426 | H-2 class I histocompatibility antigen, D-K alpha chain | H2-D1 | 6.00E-04 | 7.65 |  |
| Q61147 | Ceruloplasmin | CP | 1.19E-02 | 7.53 |  |
| P01872 | Immunoglobulin heavy constant mu | IGHM | 1.74E-03 | 7.42 |  |
| Q08879 | Fibulin-1 | FBLN1 | 2.10E-02 | 7.40 |  |
| P52430 | Serum paraoxonase/arylesterase 1 | PON1 | 2.81E-04 | 7.33 |  |
| P28665 | Murinoglobulin-1 | MUG1 | 1.10E-02 | 7.30 |  |
| P14106 | Complement C1q subcomponent subunit B | C1QB | 1.21E-03 | 7.28 |  |
| Q03311 | Cholinesterase | BCHE | 9.38E-03 | 7.27 |  |
| P01029 | Complement C4-B | C4B | 7.78E-04 | 7.25 |  |
| Q02105 | Complement C1q subcomponent subunit C | C1QC | 4.81E-04 | 7.11 |  |
| Q61702 | Inter-alpha-trypsin inhibitor heavy chain H1 | ITIH1 | 3.20E-03 | 7.04 |  |
| P06336 | Ig epsilon chain C region |  | 8.79E-03 | 7.04 |  |
| P26262 | Plasma kallikrein | KLKB1 | 5.00E-03 | 7.02 |  |
| Q9JJN5 | Carboxypeptidase N catalytic chain | CPN1 | 1.91E-03 | 7.00 |  |
| P01746 | Ig heavy chain V region 93G7 |  | 5.04E-03 | 6.97 |  |
| P07361 | Alpha-1-acid glycoprotein 2 | ORM2 | 1.03E-03 | 6.90 |  |
| Q8CG14 | Complement C1s-1 subcomponent | C1S1 | 5.40E-03 | 6.90 |  |
| P13020 | Gelsolin | GSN | 7.95E-03 | 6.77 | O |
| Q60590 | Alpha-1-acid glycoprotein 1 | ORM1 | 3.38E-03 | 6.76 |  |
| P01843 | Ig lambda-1 chain C region |  | 1.42E-03 | 6.76 |  |
| P18531 | Ig heavy chain V region 3-6 | IGHV3-6 | 7.65E-03 | 6.71 |  |
| P01844 | Ig lambda-2 chain C region | IGLC2 | 8.08E-04 | 6.67 |  |
| P04186 | Complement factor B | CFB | 4.49E-03 | 6.61 |  |
| Q61704 | Inter-alpha-trypsin inhibitor heavy chain H3 | ITIH3 | 9.33E-03 | 6.51 |  |
| P01807 | Ig heavy chain V region X44 |  | 3.92E-03 | 6.46 |  |
| P01867 | Immunoglobulin heavy constant gamma 2B | IGHG2B | 5.93E-03 | 6.41 |  |
| Q9Z1R3 | Apolipoprotein M | APOM | 2.26E-04 | 6.38 | O |
| P70274 | Selenoprotein P | SELENOP | 1.64E-02 | 6.37 | O |
| Q60994 | Adiponectin | ADIPOQ | 4.69E-04 | 6.32 |  |
| Q07456 | Protein AMBP | AMBP | 5.27E-03 | 6.30 |  |
| P00920 | Carbonic anhydrase 2 | CA2 | 6.46E-03 | 6.30 |  |
| P10605 | Cathepsin B | CTSB | 3.19E-02 | 6.30 | O |
| Q9JHH6 | Carboxypeptidase B2 | CPB2 | 2.75E-02 | 6.26 |  |
| P12246 | Serum amyloid P-component | APCS | 1.48E-03 | 6.21 |  |
| P01630 | Ig kappa chain V-II region 7S34.1 |  | 1.34E-03 | 6.11 |  |
| P18527 | Ig heavy chain V region 914 |  | 6.62E-03 | 6.07 |  |
| Q91Y97 | Fructose-bisphosphate aldolase B | ALDOB | 2.55E-03 | 5.99 |  |
| Q61703 | Inter-alpha-trypsin inhibitor heavy chain H2 | ITIH2 | 9.25E-03 | 5.92 |  |
| P01638 | Ig kappa chain V-V region L6 (Fragment) |  | 1.62E-03 | 5.86 |  |
| P01864 | Ig gamma-2A chain C region secreted form |  | 5.93E-03 | 5.84 |  |
| O08742 | Platelet glycoprotein V | GP5 | 3.92E-03 | 5.77 |  |
| P01865 | Ig gamma-2A chain C region, membrane-bound form | IGH-1A | 6.41E-03 | 5.77 |  |
| P11859 | Angiotensinogen | AGT | 8.73E-03 | 5.74 |  |
| P84750 | Ig kappa chain V region Mem5 (Fragment) |  | 1.23E-03 | 5.74 |  |
| Q8VCG4 | Complement component C8 gamma chain | C8G | 1.69E-03 | 5.71 |  |
| P01642 | Ig kappa chain V-V region L7 (Fragment) | GM10881 | 2.42E-03 | 5.63 |  |
| P21614 | Vitamin D-binding protein | GC | 1.09E-02 | 5.61 | O |
| P01869 | Ig gamma-1 chain C region, membrane-bound form | IGHG1 | 6.82E-03 | 5.53 |  |
| P49182 | Heparin cofactor 2 | SERPIND1 | 4.13E-03 | 5.45 |  |
| P97290 | Plasma protease C1 inhibitor | SERPING1 | 4.09E-03 | 5.38 |  |
| Q921I1 | Serotransferrin | TF | 5.41E-03 | 5.37 |  |
| P03987 | Ig gamma-3 chain C region |  | 1.01E-02 | 5.35 |  |
| P29788 | Vitronectin | VTN | 2.03E-03 | 5.34 |  |
| Q8VCS0 | N-acetylmuramoyl-L-alanine amidase | PGLYRP2 | 9.14E-03 | 5.34 |  |
| Q61838 | Pregnancy zone protein | PZP | 5.39E-03 | 5.34 |  |
| Q8BND5 | Sulfhydryl oxidase 1 | QSOX1 | 2.14E-03 | 5.32 |  |
| Q9DBB9 | Carboxypeptidase N subunit 2 | CPN2 | 3.77E-03 | 5.31 |  |
| P19221 | Prothrombin | F2 | 1.25E-05 | 5.29 |  |
| P01878 | Ig alpha chain C region |  | 4.99E-04 | 5.29 |  |
| Q923D2 | Flavin reductase (NADPH) | BLVRB | 1.20E-03 | 5.23 |  |
| P01801 | Ig heavy chain V-III region J606 |  | 7.23E-03 | 5.15 |  |
| O70362 | Phosphatidylinositol-glycan-specific phospholipase D | GPLD1 | 5.41E-03 | 5.12 |  |
| P01636 | Ig kappa chain V-V region MOPC 149 |  | 2.20E-03 | 5.02 |  |
| P06330 | Ig heavy chain V region AC38 205.12 |  | 1.59E-02 | 4.90 |  |
| P46412 | Glutathione peroxidase 3 | GPX3 | 5.25E-04 | 4.86 |  |
| Q03734 | Serine protease inhibitor A3M | SERPINA3M | 5.35E-03 | 4.84 |  |
| O88947 | Coagulation factor X | F10 | 1.25E-02 | 4.83 |  |
| P08607 | C4b-binding protein | C4BPA | 4.03E-03 | 4.71 |  |
| P23953 | Carboxylesterase 1C | CES1C | 8.38E-03 | 4.67 |  |
| P01632 | Ig kappa chain V-I region S107A | IGKV7-33 | 1.09E-03 | 4.60 |  |
| P01635 | Immunoglobulin kappa chain variable 12-41 (Fragment) | IGKV12-41 | 1.72E-03 | 4.57 |  |
| Q01339 | Beta-2-glycoprotein 1 | APOH | 8.03E-03 | 4.53 | O |
| Q06770 | Corticosteroid-binding globulin | SERPINA6 | 1.38E-02 | 4.52 |  |
| P01723 | Ig lambda-1 chain V region |  | 3.82E-03 | 4.48 |  |
| P01749 | Ig heavy chain V region 3 | IGHV1-61 | 2.86E-02 | 4.48 |  |
| A6X935 | Inter alpha-trypsin inhibitor, heavy chain 4 | ITIH4 | 3.21E-04 | 4.46 |  |
| P18524 | Ig heavy chain V region RF |  | 1.27E-02 | 4.45 |  |
| P97298 | Pigment epithelium-derived factor | SERPINF1 | 1.98E-02 | 4.41 |  |
| Q61129 | Complement factor I | CFI | 1.36E-02 | 4.39 |  |
| P42703 | Leukemia inhibitory factor receptor | LIFR | 2.54E-02 | 4.36 |  |
| Q61247 | Alpha-2-antiplasmin | SERPINF2 | 8.72E-06 | 4.35 |  |
| P01728 | Ig lambda-2 chain V region |  | 1.29E-03 | 4.33 |  |
| Q9QWK4 | CD5 antigen-like | CD5L | 1.82E-02 | 4.32 |  |
| Q64726 | Zinc-alpha-2-glycoprotein | AZGP1 | 1.22E-02 | 4.30 |  |
| P01633 | Immunoglobulin kappa chain variable 6-17 | IGKV6-17 | 4.61E-03 | 4.28 |  |
| P01631 | Ig kappa chain V-II region 26-10 |  | 1.23E-03 | 4.28 |  |
| Q8CG16 | Complement C1r-A subcomponent | C1RA | 1.98E-03 | 4.24 |  |
| P01660 | Ig kappa chain V-III region PC 3741/TEPC 111 |  | 4.43E-03 | 4.24 |  |
| Q01279 | Epidermal growth factor receptor | EGFR | 2.15E-02 | 4.16 |  |
| P01837 | Immunoglobulin kappa constant | IGKC | 7.19E-04 | 3.95 |  |
| P01670 | Ig kappa chain V-III region PC 6684 |  | 7.70E-03 | 3.84 |  |
| P11680 | Properdin | CFP | 2.82E-02 | 3.82 |  |
| Q00724 | Retinol-binding protein 4 | RBP4 | 1.09E-03 | 3.81 | O |
| P01027 | Complement C3 | C3 | 3.81E-03 | 3.75 |  |
| O89020 | Afamin | AFM | 2.24E-03 | 3.75 |  |
| Q61646 | Haptoglobin | HP | 4.01E-04 | 3.66 |  |
| P82198 | Transforming growth factor-beta-induced protein ig-h3 | TGFBI | 3.41E-03 | 3.54 |  |
| Q60963 | Platelet-activating factor acetylhydrolase | PLA2G7 | 1.76E-02 | 3.50 |  |
| P06683 | Complement component C9 | C9 | 3.56E-02 | 3.43 |  |
| Q9Z126 | Platelet factor 4 | PF4 | 1.77E-02 | 3.31 |  |
| P32261 | Antithrombin-III | SERPINC1 | 5.64E-03 | 3.28 |  |
| Q9ESB3 | Histidine-rich glycoprotein | HRG | 1.29E-03 | 3.09 |  |
| Q06890 | Clusterin (Apolipoprotein J) | CLU | 3.25E-03 | 3.06 | O |
| O08677 | Kininogen-1 | KNG1 | 2.39E-03 | 2.87 |  |
| Q91X72 | Hemopexin | HPX | 1.35E-02 | 2.85 |  |
| P08226 | Apolipoprotein E | APOE | 9.95E-04 | 2.82 | O |
| P07724 | Albumin | ALB | 2.15E-02 | 2.81 | O |
| P01887 | Beta-2-microglobulin | B2M | 2.45E-02 | 2.75 | O |
| P02089 | Hemoglobin subunit beta-2 | HBB-B2 | 7.91E-03 | 2.39 | O |
| P31532 | Serum amyloid A-4 protein | SAA4 | 2.68E-02 | 2.27 |  |
| P07309 | Transthyretin | TTR | 3.23E-02 | 1.67 | O |

**Table S6.** Sequence for preparation of ASO-loaded D-Cb. The asterisk denotes phosphorothioate modification.

| S6-T5-ASO (PLK1) | CTT AAT GAC TTT GGC CGG CGC TTT GAC CTT CTG CTT ATG TCC CCT A TTT TTC* A*TT AAG CAG CT*C* G |
| --- | --- |
| S6-T5-ASO (SC) | CTT AAT GAC TTT GGC CGG CGC TTT GAC CTT CTG CTT ATG TCC CCT A TTT TTC* A*GGGCTGACAG*C* G |
| ASO (Ctrl) | C* A*TT AAG CAG CT*C* G |

**Table S7.** Primer sequence for qRT-PCR to check mRNA level of PLK1 in U87MG cells or brain of GBM mouse model. F denotes forward primer, R denotes reverse primer.

| PLK1 | **F** | GCACAGTGTCAATGCCTCCAAG |
| --- | --- | --- |
|  | **R** | GCCGTACTTGTCCGAATAGTCC |
| GAPDH | F | GTCTCCTCTGACTTCAACAGCG |
|  | R | ACCACCCTGTTGCTGTAGCCAA |

1. Tyanova S, Temu T, Sinitcyn P., Carlson A, Hein MY, Geiger T, Mann M, Cox J. The Perseus computational platform for comprehensive analysis of (prote)omics data. *Nat Methods* 2016;**13**:731-740.
